# Enzymkatalysierte späte Modifizierungen: Besser spät als nie

**DOI:** 10.1002/ange.202014931

**Published:** 2021-03-08

**Authors:** Elvira Romero, Bethan S. Jones, Bethany N. Hogg, Arnau Rué Casamajo, Martin A. Hayes, Sabine L. Flitsch, Nicholas J. Turner, Christian Schnepel

**Affiliations:** ^1^ School of Chemistry The University of Manchester Manchester Institute of Biotechnology 131 Princess Street Manchester M1 7DN Vereinigtes Königreich; ^2^ Compound Synthesis and Management Discovery Sciences, BioPharmaceuticals R&D AstraZeneca Götheborg Schweden

**Keywords:** Diversifizierung, Enzym-Engineering, Oxyfunktionalisierung, Selektivität, Späte Funktionalisierung

## Abstract

Die Enzymkatalyse gewinnt zunehmend an Bedeutung in der Synthesechemie. Die durch Bioinformatik und Enzym‐Engineering stetig wachsende Zahl von Biokatalysatoren eröffnet eine große Vielfalt selektiver Reaktionen. Insbesondere für späte Funktionalisierungsreaktionen ist die Biokatalyse ein geeignetes Werkzeug, das oftmals der konventionellen De‐novo‐Synthese überlegen ist. Enzyme haben sich als nützlich erwiesen, um funktionelle Gruppen direkt in komplexe Molekülgerüste einzuführen sowie für die rasche Diversifizierung von Substanzbibliotheken. Biokatalytische Oxyfunktionalisierungen, Halogenierungen, Methylierungen, Reduktionen und Amidierungen sind von besonderem Interesse, da diese Strukturmotive häufig in Pharmazeutika vertreten sind. Dieser Aufsatz gibt einen Überblick über die Stärken und Schwächen der enzymkatalysierten späten Modifizierungen durch native und optimierte Enzyme in der Synthesechemie. Ebenso werden wichtige Beispiele in der Wirkstoffentwicklung hervorgehoben.

## Graphical abstract

Späte Modifizierungen sind unerlässlich für die Diversifizierung von Verbindungen in der Synthese. Da Enzyme effiziente Werkzeuge für milde, Katalysator‐kontrollierte Funktionalisierungen bieten, sind neue biokatalytische Methoden für eine große Fülle orthogonaler Reaktionen entstanden. In diesem Aufsatz wird das breite Spektrum der späten Biotransformationen besprochen, und verschiedene Enzymklassen werden anhand aktueller Beispiele diskutiert.

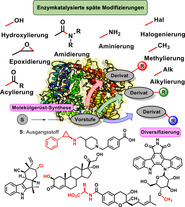

## 1 Einleitung

### 1.1 Frühe vs. späte Modifizierung multifunktioneller Substanzen

Die Funktionalisierung von C‐H‐Bindungen ist fundamental für die Synthesechemie; jedoch handelt es sich dabei um eine der schwierigsten Reaktionen. Seit langer Zeit ist die Modifizierung nicht‐aktivierter Kohlenstoffverbindungen in Alkanen oder Arenen unersetzlich, um Kohlenstoff‐basierte Ausgangsstoffe zu erhalten.^[1]^ Beispielsweise wird die radikalische Halogenierung von Aliphaten traditionell zur Funktionalisierung von C‐H‐Bindungen genutzt. Im Gegensatz dazu stellt die selektive Modifizierung deutlich komplexerer Moleküle eine besondere Herausforderung dar, da Orthogonalität und Kompatibilität zu bereits vorhandenden Funktionalitäten essenzielle Voraussetzungen sind, um Diversifizierung zu erzielen.

Die späte Modifizierung im finalen Schritt innerhalb einer mehrschrittigen Syntheseroute ermöglicht die gezielte Diversifizierung, wodurch C‐H‐ und C‐C‐Bindungen in Anwesenheit anderer funktioneller Gruppen selektiv adressiert werden (Schema 1). Neben zahlreichen Anwendungsgebieten profitieren inbesondere die Wirkstoffentwicklung und Naturstoffderivatisierung von Errungenschaften der späten Funktionalisierung: Typischerweise werden Substanzbibliotheken aus leicht zugänglichen Bausteinen aufgebaut und selektiv verändert. Dieses Vorgehen ebnet den Weg zum Erfolg in der pharmazeutischen Entwicklung. Trotz offensichtlicher Vorteile dieser Methode sind Kompatibilität und die Vermeidung von Kreuzkreaktivitäten wichtige Kriterien, die erfüllt werden müssen.

**Scheme 1:**
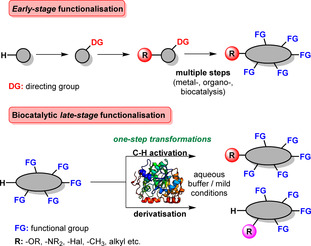
Die frühe Funktionalisierung beruht auf der schrittweisen Derivatisierung eines nicht‐funktionalisierten Ausgangsstoffs. Biokatalytische Konzepte zur späten Funktionalisierung vielfältiger Molekülgerüste erlauben ortsspezifische Transformationen in Anwesenheit anderer funktioneller Gruppen.

Zweifelsfrei befindet sich die Metallkatalyse an der Spitze der späten Funktionalisierungen, wobei C‐O‐, C‐N‐ und C‐C‐Bindungsbildung sowie Halogenierung besonders begehrte Transformationen sind.^[2]^ Darüber hinaus haben sich auch die Organokatalyse, Photo‐ und Elektrochemie als nützlich in diesem Feld erwiesen.[^3^, ^4^, ^5^] Es ist erfreulich, dass sich seit einigen Jahren die Biokatalyse als eine neue Methode für späte Funktionalisierungen herausgestellt hat. Trotz einer immens hohen Anzahl hochfunktionalisierter Moleküle innerhalb einer Zelle ermöglichen Enzyme die Synthese komplexer Metabolite in wässrigen Systemen, wobei Schutzgruppen oder dirigierende Gruppen nicht erforderlich sind. Somit repräsentieren Enzyme ideale Katalysatoren für späte Funktionalisierungen.

Heutzutage nutzen die Grund‐ sowie die Feinchemikalienindustrie Enzymkatalysatoren in Produktionsprozessen, um Selektivität und Nachhaltigkeit zu erzielen. Die Bedeutung biokatalytischer Transformationen für die Industrie wurde kürzlich von Wu et al. ausführlich in einem Aufsatz dargestellt.^[6]^

Im Jahr 2018 wurde ein Aufsatz über die Perspektiven, wie Biokatalyse und Chemokatalyse sich in der Retrosynthese gegenseitig ergänzen können, veröffentlicht.^[7]^ Enorme Anstrengungen in der Forschung sowie eine schier endlose Zahl neuer Enzyme tragen dazu bei, das große Potenzial der späten Modifizierungen kontinuierlich auszubauen. Sowohl unter dem Gesichtspunkt der Wirkstoffentwicklung als auch für Hochdurchsatz‐Experimente ist dies von zunehmendem Interesse, da in diesen Bereichen die Verfügbarkeit orthogonaler und robuster Methoden besonders erwünscht ist.^[8]^ Somit haben uns der Einzug später Biotransformationen und deren steigende Relevanz in der Synthese dazu motiviert, die vielfältigen jüngsten Errungenschaften in diesem Feld darzustellen. Dieser Aufsatz präsentiert eine straffe, aktuelle Übersicht, die ein besseres Verständnis über das Spektrum und die Grenzen der späten Biokatalyse vermitteln soll.

### 1.2 Wege zur Erzeugung von Biokatalysatordiversität

**Figure 1:**
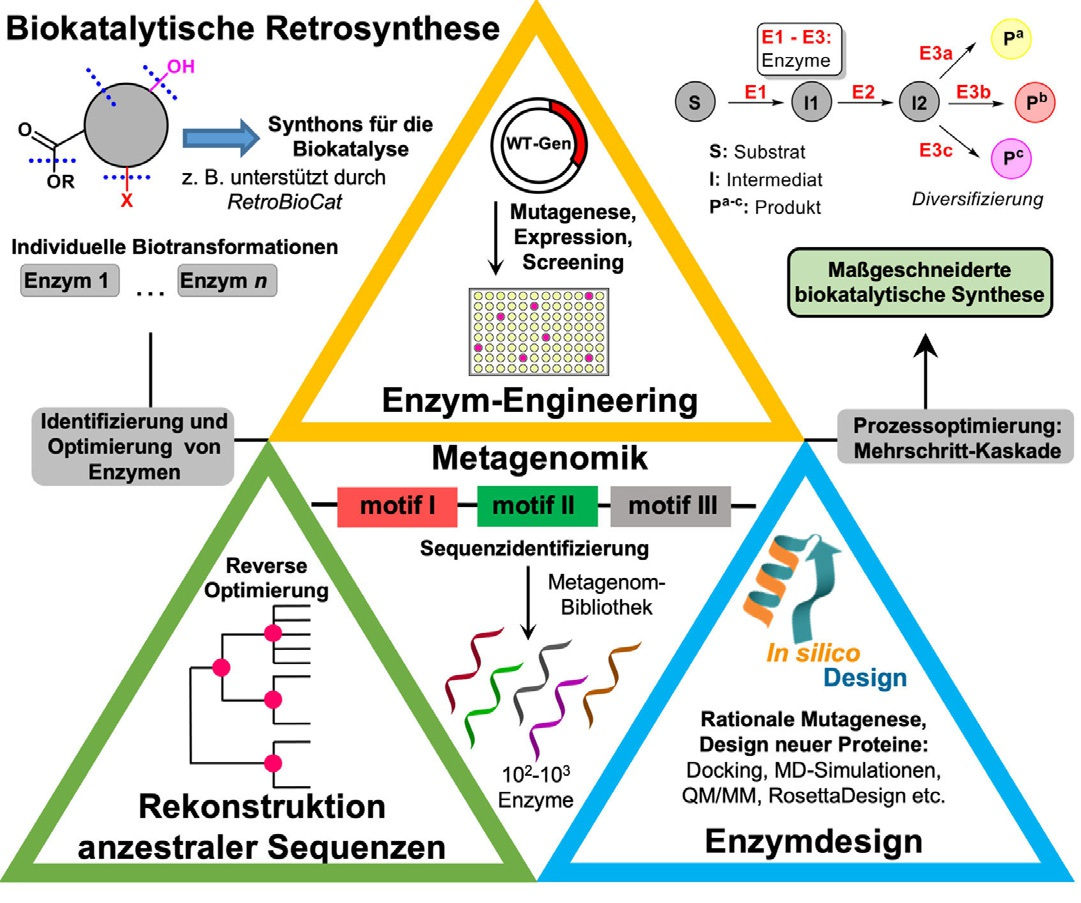
Moderne Route in Richtung eines maßgeschneiderten Bioprozesses von der Retrosynthese bis hin zur abschließenden Diversifizierung. Die Pyramide zeigt aktuelle Ansätze, die häufig kombiniert werden, um diverse Biokatalysatoren zu erzeugen. Die finale enzymatische Modifizierung nimmt eine besondere Stellung ein, da sie Zugang zu einer Vielzahl von Derivaten eröffnet, was von der Wahl des Biokatalysators abhängig ist.

In den vergangenen Jahrzehnten ist die Nutzung enzymatischer Prozesse deutlich zukunftsfähiger geworden.[^9^, ^10^, ^11^] Wie in der konventionellen Synthese, sind das Vorhandensein eines oder sogar zahlreicher (Bio‐)Katalysatoren sowie optimierte Reaktionsbedingungen kritische Punkte, wenn enzymkatalysierte Schritte, z. B. für die Modifizierung von Leitwirkstoffen, ausgewählt werden. Die biokatalytische Retrosynthese kann dabei angewendet werden, um plausible Transformationen zu identifizieren, wobei dieses Verfahren durch Computer‐gestützte Syntheseplanung unterstützt werden kann.^[12]^ Daraufhin sind oftmals die Identifizierung oder das Design und Engineering notwendig, bis eine optimierte biokatalytische Route hervorgeht (Abbildung 1).

Heutzutage ist die Metagenomik sehr zweckmäßig, um neue Biokatalysatoren aus Umweltproben zu erschließen. Das Enzym‐Engineering erlaubt die Optimierung eines Katalysators auf eine gewünschte Reaktion, sodass wichtige Prozesskriterien erfüllt sind.^[13]^ Zweifellos sind die gerichtete Evolution sowie ortsgerichtete und Sättigungsmutagenese sehr leistungsstarke Methoden, um Proteine zu verändern und maßzuschneidern.[^14^, ^15^] Seit kurzer Zeit gewinnt auch die Rekonstruktion anzestraler Sequenzen an Bedeutung. Diese ermöglicht die Generierung artifizieller Vorfahren der heute bekannten Varianten, die oftmals eine höhere Widerstandsfähigkeit und ein verbreitertes Substratspekrum aufweisen.[^16^, ^17^] Weitere Information über die Strategien zur Diversifizierung von Biokatalysatoren können in Supporting Section 1 nachgelesen werden (siehe Hintergrundinformationen).

### 1.3 Hintergrund zur Aktivierung von C‐H‐Bindungen

Im Allgemeinen wird C‐H‐Reaktivität durch die Bindungsstärke vorgegeben, was wiederum die Selektivität der Aktivierungsreaktion bestimmt. Trotz umfangreicher Untersuchungen zur C‐H‐Funktionalisierung lassen sich die Abhängigkeit zwischen Bindungsenergie und Reaktivität sowie das Ergebnis einer Reaktion in manchen Fällen schwer vorhersagen.^[18]^

Es ist nicht überraschend, dass die Merkmale einer C‐H‐Bindung von ihrer Umgebung beeinflusst werden, sodass elektronische sowie sterische Effekte vorgeben, auf welche Weise eine Bindung adressiert wird.^[19]^ In Tabelle S1 sind verschiedene Bindungsstärken und deren Einfluss auf die Reaktivität zusammengefasst. Dies liefert einen Einblick, wie die chemische Umgebung die Dissoziationsenergie beeinflusst. Insgesamt sind gerichtete sowie Katalysator‐kontrollierte Funktionalisierungen typische Ansätze zur Aktivierung von C‐H‐Bindungen (Schema 2).[^20^, ^21^, ^22^] In einem gerichteten Ansatz werden bereits vorhandene funktionelle Gruppen genutzt, um beispielsweise ein Metallion zu komplexieren, während im optimalen Fall einer Katalysator‐kontrollierten Reaktion lediglich der (Bio‐)Katalysator zwischen den Reaktionszentren diskriminiert und damit eine ortsspezifische Modifizierung erlaubt.

**Scheme 2:**
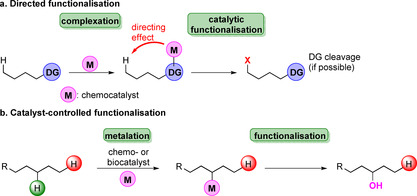
Ansätze zur Funktionalisierung von Kohlenwasserstoffen. a) Gerichtete und b) Katalysator‐kontrollierte Aktivierung werden heutzutage häufig angewendet, um C‐H‐Bindungen zu adressieren. In (b) ist beispielhaft eine regioselektive Hydroxylierung gezeigt, in welcher der Katalysator zwischen verschiedenen C‐H‐Positionen diskriminiert.

## 2 Oxyfunktionalisierung: Vielfältige Wege zum Aufbau von C‐O‐Bindungen

**Figure 2:**
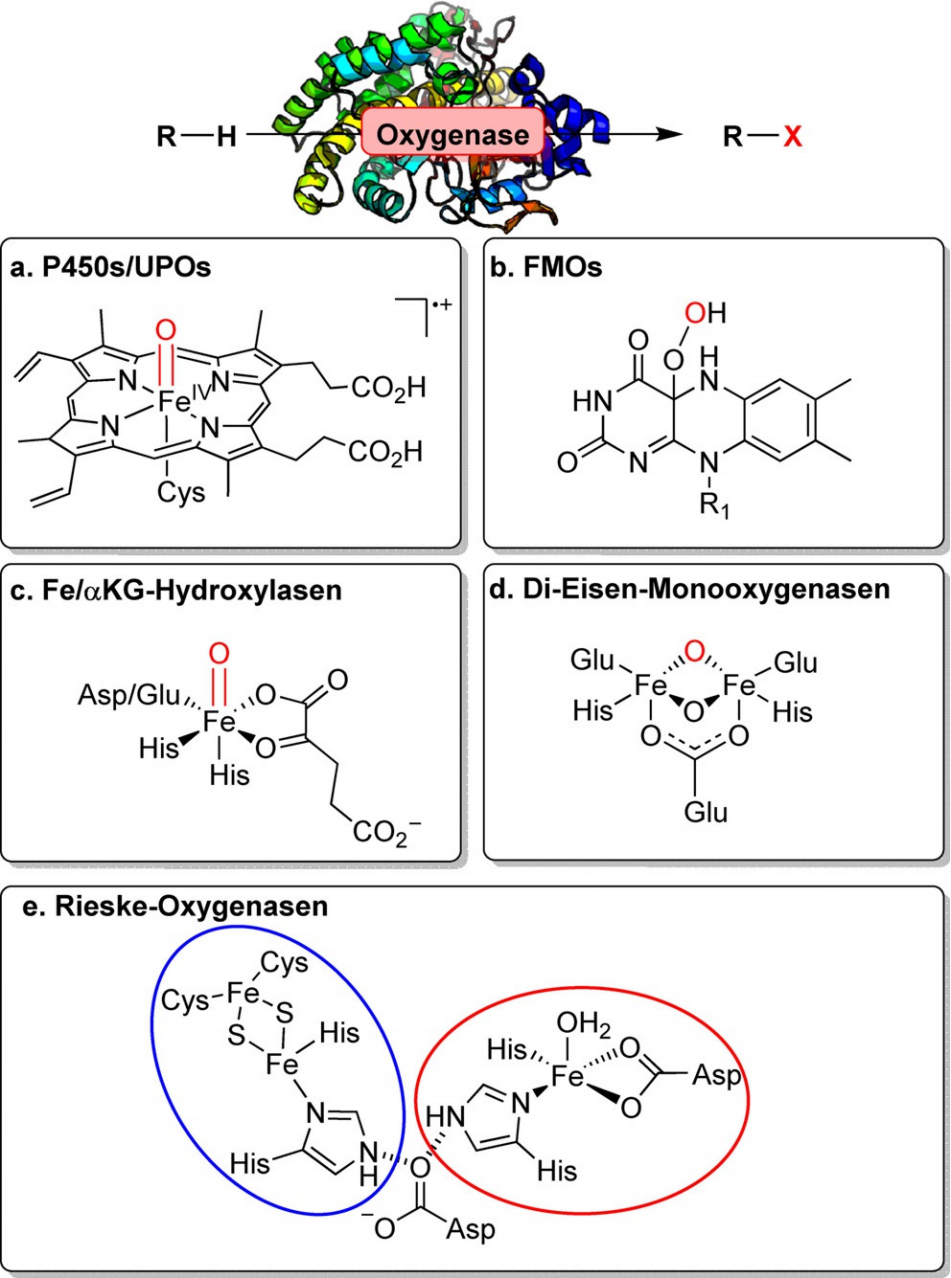
Darstellung der reaktiven Spezies verschiedener Oxygenase‐Klassen, welche die enyzmatische Oxyfunktionalisierung ermöglichen. a) P450‐Mono‐ und ‐Peroxygenasen: aktives Zentrum mit Häm‐Thiolat in der reaktiven “Compound I”‐Zwischenstufe gezeigt. b) Flavin‐abhängige Monooxygenasen: Flavin‐Hydroperoxid. c) Fe/αKG‐abhängige Hydroxylasen: Die Nicht‐Häm‐Eisen‐Oxo‐Spezies induziert eine Radikalabstraktion am Substrat. d) Di‐Eisen‐Monooxygenasen: Ein Nicht‐Häm‐di‐Eisen‐Komplex im aktiven Zentrum verbrückt die reaktive Oxo‐Spezies. e) Rieske‐Oxygenasen: Nicht‐Häm‐Eisen‐Zentrum (rot) ist zum [2Fe‐2S]‐Cluster (blau) verbrückt (Abbbildung angepasst nach Barry et al.).^[31]^

Die Oxyfunktionalisierug stellt ein breites Repertoire an verschiedenen Mono‐, Di‐ und Peroxygenasen zur Verfügung, welche die Oxidation nichtaktivierter C‐O‐Bindungen ermöglichen und damit einen breiten Werkzeugkasten für späte Funktionalisierungen bereitstellen.[^23^, ^24^] Obwohl diese Transformation hauptsächlich von Cytochrom‐P450‐Monooxygenasen (P450) katalysiert wird, ist eine große Bandbreite weiterer Biokatalysatoren verfügbar, insbesondere Peroxygenasen (UPO),[^25^, ^26^] Flavin‐abhängige Monooxygenasen,[^27^, ^28^] Eisen/α‐Ketoglutarat‐abhängige Hydroxylasen,^[29]^ Di‐Eisen‐^[30]^ und Rieske‐Monooxygenasen.[^31^, ^32^] Die Diversität dieser Enzyme spiegelt sich in deutlich verschiedenen aktiven Zentren sowie mechanistischen Differenzen wider (Abbildung 2). Eine detaillierte Beschreibung zum Hintergrund findet sich in Supporting Section 3 (siehe Hintergrundinformationen). In diesem Abschnitt heben wir insbesondere neue und herausragende Fortschritte in der späten Funktionalisierung hervor, wobei die Verbindung der Oxyfunktionalisierungen mit Folgemodifikationen besonders berücksichtigt wird.

### 2.1 Hydroxylierung

Aufgrund ihrer strukturellen Vielfalt und biologischen Aktivität sind Naturstoffe eine bedeutende Quelle für Wirkstoffgerüste.^[33]^ In vielen Biosynthesewegen sind P450‐Enzyme weit verbreitete Katalysatoren für die späte In‐vivo‐Oxyunktionalisierung, wie sich in der Fülle an Naturstoffen, Wirkstoff‐Substraten und Metaboliten widerspiegelt.[^34^, ^35^, ^36^, ^37^]

**Figure 3:**
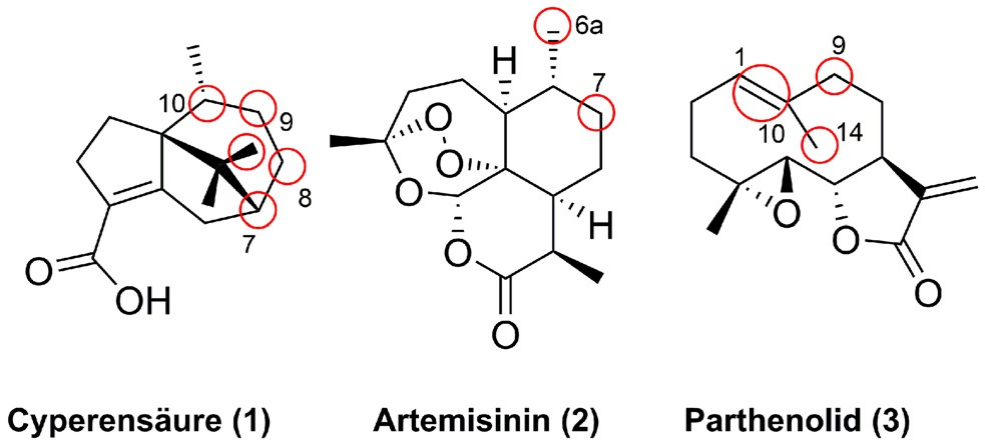
Regio‐ und stereoselektive Hydroxylierung der Sesquiterpene (Hydroxylierungspositionen: rote Kreise).

Die Hydroxylierung ist häufig essenziell für die weiterführende Modifikation von Naturstoffen. Dabei ist die Diversifizierung bioaktiver Verbindungen ein hocheffizienter Ansatz zur Untersuchung von Struktur‐Aktivitäts‐Beziehungen. Beispielsweise wurde dies für die Naturstoffklasse der Sesquiterpene gezeigt. In‐vivo‐Funktionalisierung der anti‐angiogenen Cyperensäure (**1**) wurde mithilfe des Pilzstammes *Cunninghamella elegans* AS 3.2028 ermöglicht (Abbildung 3): Es wurden verschiedene Isomere, C*7S*‐, C8*S*‐, C9*R*‐, C10*S*‐, und das C11*R*‐hydroxylierte Produkt erhalten, wobei die C7*S*‐ und C9*R*‐Hydroxyisomere besonders gute Cytoxizität gegenüber zwei Tumorzelllinien (HepG2 and MCF‐7) aufwiesen.^[38]^ Diese Beispiele untermauern die Relevanz der späten Funktionalisierung, um biologische Funktionen zu modulieren, indem eine entscheidende strukturelle Modifikation eingeführt wird. Anstatt eines In‐vivo‐Ansatzes wurde später das weitverbreitete Enzym P450‐BM3 rational optimiert, um **1** zu hydroxylieren. Dieser Ansatz lieferte effiziente Enzymvarianten, welche die bioaktiven C7*S*‐ und C9*R*‐Hydroxylierungen in hervorragender Selektivität vermittelten. Diese Ergebnisse unterstreichen die gewünschte Formbarkeit von P450‐Enzymen im Hinblick auf die Diversifizierung eines komplexen Sesquiterpengerüsts.^[39]^

Die Optimierung einer anfänglich nichtselektiven P450‐BM3‐Variante machte eine Reihe verschiedener hydroxylierter Produkte des Sesquiterpenlactons Artemisin (**2**) zugänglich. Drei der erzeugten Varianten zeigten einen besonders hohen Turnover gegenüber der regio‐ und stereoselektiven Hydroxylierung der drei C(sp^3^)‐H‐Positionen und bieten somit eine chemoenzymatische Plattform für neuartige Antimalaria‐Wirkstoffe.^[40]^ In ähnlicher Weise gelang die späte Diversifizierung des antikarzinogenen Sesquiterpenlactons Parthenolid (**3**). Ausgehend von der gleichen P450‐BM3‐Variante sollten die beiden C(sp^3^)‐H‐Zentren (C9 und C14) und die C1,C10‐Doppelbindung oxidiert werden. Drei Enzyme gingen aus der Evolution hervor, die letztlich die Epoxidierung und Hydroxylierung von Verbindung **3** ermöglichten und somit wichtige Grundbausteine für die Synthese weiterer bioaktiver Derivate lieferten.^[41]^

Darüber hinaus war im Rahmen der Totalsynthese des Norditerpenalkaloids Nigelladin A (**6**) eine optimierte P450‐BM3‐Variante in der Lage, die Oxidation des allylischen C7‐Atoms zu katalysieren, womit eine anschließende Dess‐Martin‐Oxidation zum gewünschten Zielmolekül möglich wurde (Schema 3). Diese Anwendung unterstreicht den Vorteil der Biokatalyse, da traditionelle chemische Oxidationsmittel in unzureichender Selektivität und Überoxidation resultierten.^[42]^

**Scheme 3:**
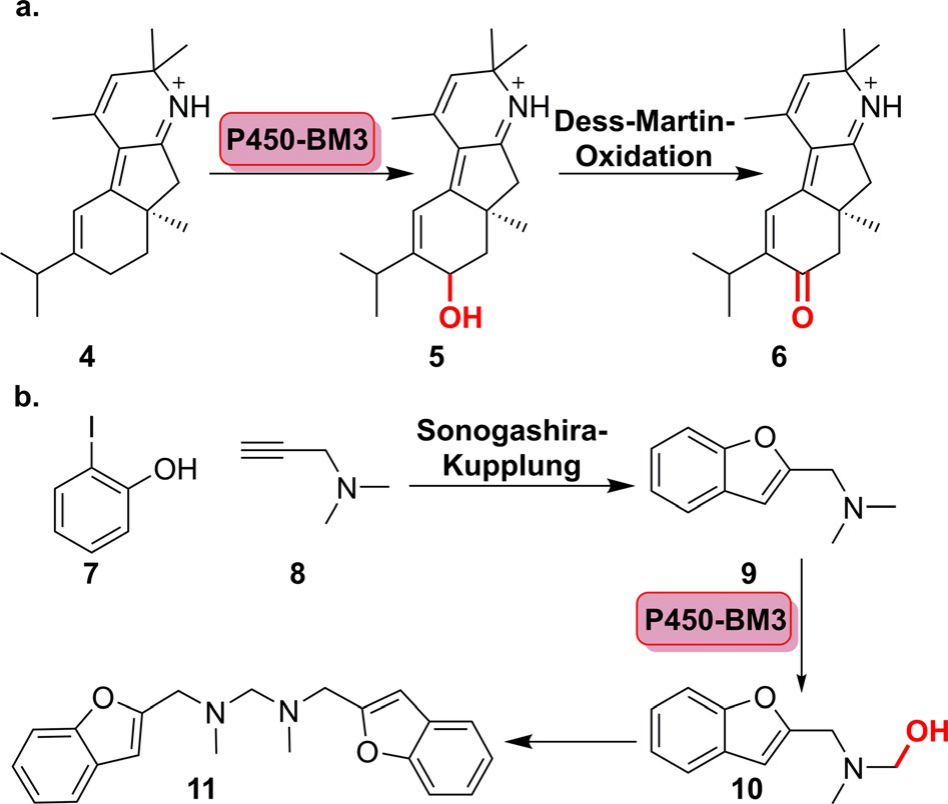
Chemoenzymatische Synthese durch optimierte P450‐BM3‐Varianten: a) Hydroxylierung ermöglicht die anschließende Dess‐Martin‐Oxidation zum Norditerpenoidalkaloid Nigelladin A (**6**). b) Eine vorangegangene Sonogashira‐Reaktion, gefolgt von einer Hydroxylierung mit einem P450‐Enzym, erlaubt Zugang zum bis‐2‐substituierten Benzofuran‐Derivat (**11**).

**Figure 4:**
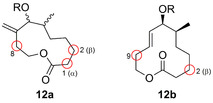
Regioselektive Hydroxylierung der Pikromycin‐Motive **12 a** und **12 b**. Diversifizierungspositionen sind durch einen roten Kreis gekennzeichnet.

Zudem wurde die chemoenzymatische Synthese in einer Zwei‐Schritt‐Eintopfkaskade gezeigt, wobei der erste Schritt eine Palladium‐freie Sonogashira‐Kreuzkupplung zum Benzofuran (**9**) war. Dieser schloss sich eine Hydroxylierung mithilfe einer BM3‐Variante an, sodass nach Freisetzung von Formaldehyd das bis‐2‐substituierte Produkt (**11**) entstand.^[43]^ Außerdem wurde die chemoenzymatische regio‐ und stereochemische Diversifizierung des Makrozyklus des Pikromycins (**12 a**/**b**) via Click‐Chemie und Veresterung mit nachfolgender Hydroxylierung durch eine optimierte Variante des P450‐Enzyms PikC ermöglicht (Abbildung 4).^[44]^ Diese Studie zeigt, wie eine P450‐Dreifachmutante aus einem Biosyntheseweg erfolgreich in einen synthetisch anwendbaren Biokatalysator für die späte Modifikation zyklischer Motive umgewandelt werden kann.

**Figure 5:**
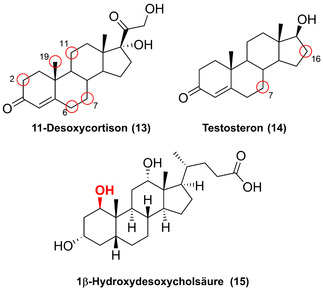
Regio‐ und stereoselektive Hydroxylierung verschiedener Steroide. Diversifizierte Positionen sind durch einen roten Kreis gekennzeichnet.

Die regio‐ und stereoselektive späte Hydroxylierung von Steroiden erweist sich gewöhnlich als eine besondere Herausforderung, die oftmals nur in Mehrschritt‐Synthesen möglich ist. Dennoch ist die selektive Dekoration des Steroidgerüsts essenziell für die Wirkstoffsynthese.^[45]^ Dieses Problem kann mithilfe von P450‐Enzymen umgangen werden: Es wurde gezeigt, dass verschiedene Wildtyp‐P450‐Enzyme aus Pilzen (STH10, CYP5150AP2, CYP5150AP3 und CYP5150AN1) eindeutige Regioselektivitäten gegenüber der Hydroxylierung des 11‐Desoxycortisons (**13**) besitzen. Dabei wurden die Isomere 19‐, 11β‐, 7β‐, 6β‐ und 2β‐Hydroxy‐11‐desoxycortison erhalten (Abbildung 5).[^46^, ^47^] Der Zugang zur C19‐Hydroxylierung ist ausschlaggebend für die Produktion von 19‐norsteroidalen Pharmazeutika, während steroidale 7β‐Alkohole anti‐inflammatorische und neuroprotektive Eigenschaften besitzen.[^46^, ^48^, ^49^]

In einer kürzlich erschienenen Studie wurde P450‐BM3 optimiert, um effizient die Funktionalisierung sechs verschiedener Steroidderivate mit hoher Regio‐ und Stereoselektivität (**14**; Adrenosteron; Nandrolon; Epistosteron; Androstenedion; d‐Ethylgonendion) zu katalysieren, sodass die entsprechenden 7β‐Alkohole isoliert wurden. Durch gerichtete Evolution verlief auch die C16‐Hydroxylierung mit deutlich abgegrenzten α‐ und β‐Diastereoselektivitäten erfolgreich, wie für ein anderes Set von Steroiden gezeigt.[^50^, ^51^] Ebenso wurde eine P450‐BM3‐Mutante für die selektive Synthese des Steroids 1β‐Hydroxydesoxycholsäure (**15**) genutzt, womit auch dessen deuteriertes Analogon im Milligramm‐Maßstab hergestellt wurde.^[52]^

Das Screening von P450‐BM3‐Bibliotheken erwies sich als ein wertvoller Ansatz für die späte Oxidation humaner Wirkstoffe, wie z. B. Chlorzoxazon, Testosteron (**14**), Amitriptylin, Lidocain, Diclofenac, Naproxen und Noscapin, welches somit die Bedeutung P450‐katalysierter Hydroxylierungen für die Synthese mutmaßlicher Wirkstoffmetabolite hervorhebt.[^53^, ^54^]

**Figure 6:**
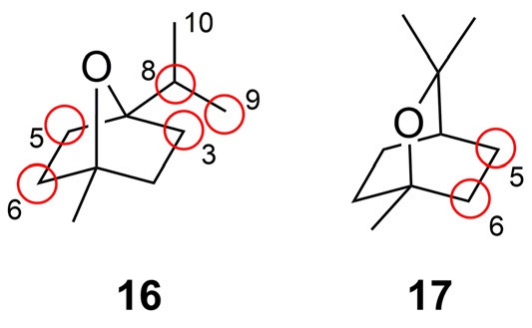
Regioselektive Hydroxylierung achiraler Terpene: 1,4‐Cineol (**16**) und 1,8‐Cineol (**17**). Diversifizierungspositionen sind in Rot gekennzeichnet.

Die Entwicklung von P450‐Enzymen für die Produktion nützlicher oxygenierter Terpenintermediate für die weitere Funktionalisierung eröffnet einen Ausganspunkt für wertvolle Carotenoide und Vitamine.^[55]^ Zum Beispiel erleichtern P450cam und P450‐BM3 eine Verschiebung der Hydroxylierungsselektivität der Monoterpene 1,4‐ (**16**) und 1,8‐Cineol (**17**), wodurch zwei Stereozentren aufgebaut werden (Abbildung 6).^[56]^

**Figure 7:**
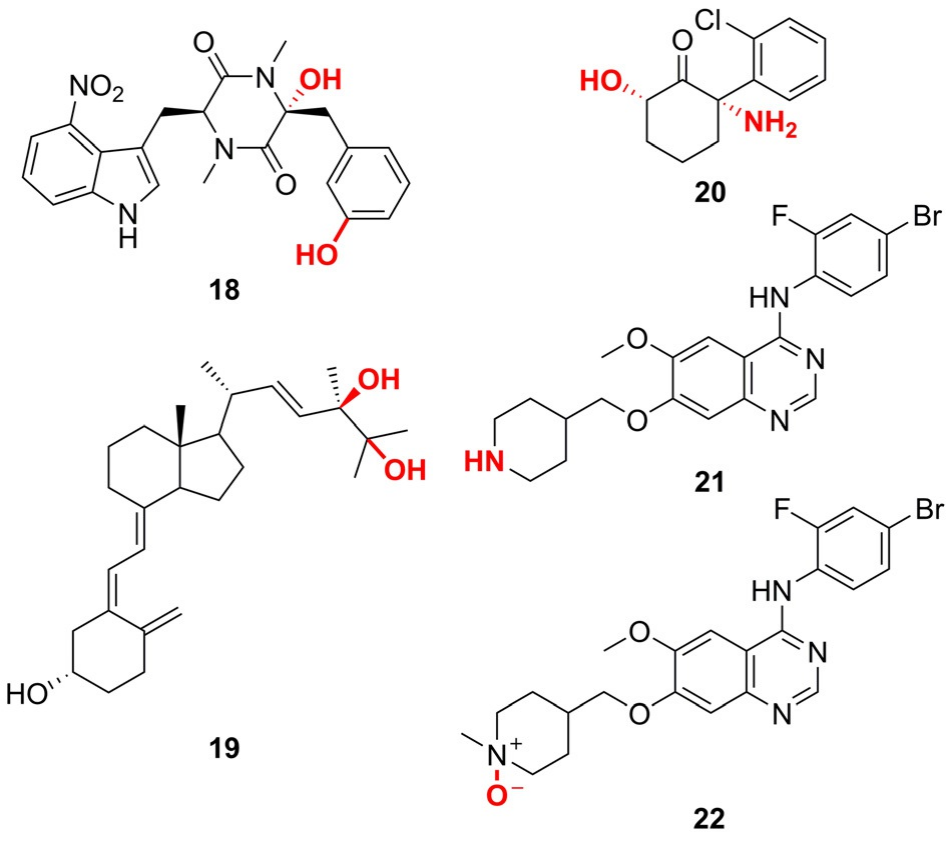
Multi‐Oxyfunktionalisierung in der späten Funktionalisierung. Die aromatische und aliphatische P450‐TxtC‐katalysierte Hydroxylierung liefert Diketopiperazin‐Derivat **18**, während die Dihydroxylierung von Vitamin D2, katalysiert von CYP109E1, die Verbindung **19** ergibt. Die konsekutive oxidative N‐Demethylierung und C6‐Hydroxylierung von (*S*)‐Ketamin zu **20** wird durch CYP154E1 ermöglicht. Oxidation von Vandetanib: Ein P450‐Enzym katalysiert die Demethylierung via Oxidation, die schließlich *N*‐Desmethylvandetanib (**21**) ergibt, während eine FMO‐katalysierte Oxidation Vandetanib‐*N*‐oxid (**22**) liefert.

Die Multi‐Oxyfunktionalisierung bietet einen zusätzlichen Ansatz, der in der Biokatalyse weit ausgeschöpft wird. Das Wildtypenzym P450 TxtC ermöglicht aliphatische sowie aromatische C‐H‐Hydroxylierungen in der späten Funktionalisierung eines Diketopiperazins (**18**).[^57^, ^58^] Zudem berichten aktuelle Beispiele über die Dihydroxylierung zweier aliphatischer oder aromatischer C‐H‐Bindungen innerhalb eines Vitamin‐D2‐Motivs bzw. Arens:[^59^, ^60^] Wildtyp‐CYP109E1 hydroxyliert Vitamin D2 hochgradig regio‐ und stereoselektiv in einer Zwei‐Schritt‐Dihydroxylierung (Abbildung 7).^[59]^

Darüber hinaus wurden P450‐Enzyme im Hinblick auf multiple Oxyfunktionalisierungen konstruiert.[^60^, ^61^] Eine Dreifachmutante von CYP154E1 bewerkstelligte die konsekutive oxidative N‐Demethylierung sowie regio‐ und stereoselektive C6‐Hydroxylierung. Damit konnte das Antidepressivum (2*S*,6*S*)‐Hydroxynorketamin (**20**) aus (*S*)‐Ketamin erhalten werden.^[61]^ Die Anwendung multipler Enzyme für verschiedene Oxyfunktionalisierungen wurde für den Antitumor‐Kinaseinhibitor Vandetanib berichtet. Der Wirkstoff wurde zu *N*‐Desmethylvandetanib (**21**) und Vandetanib‐*N*‐oxid (**22**) mittels einer P450 und einer FMO oxidiert.^[62]^

**Figure 8:**
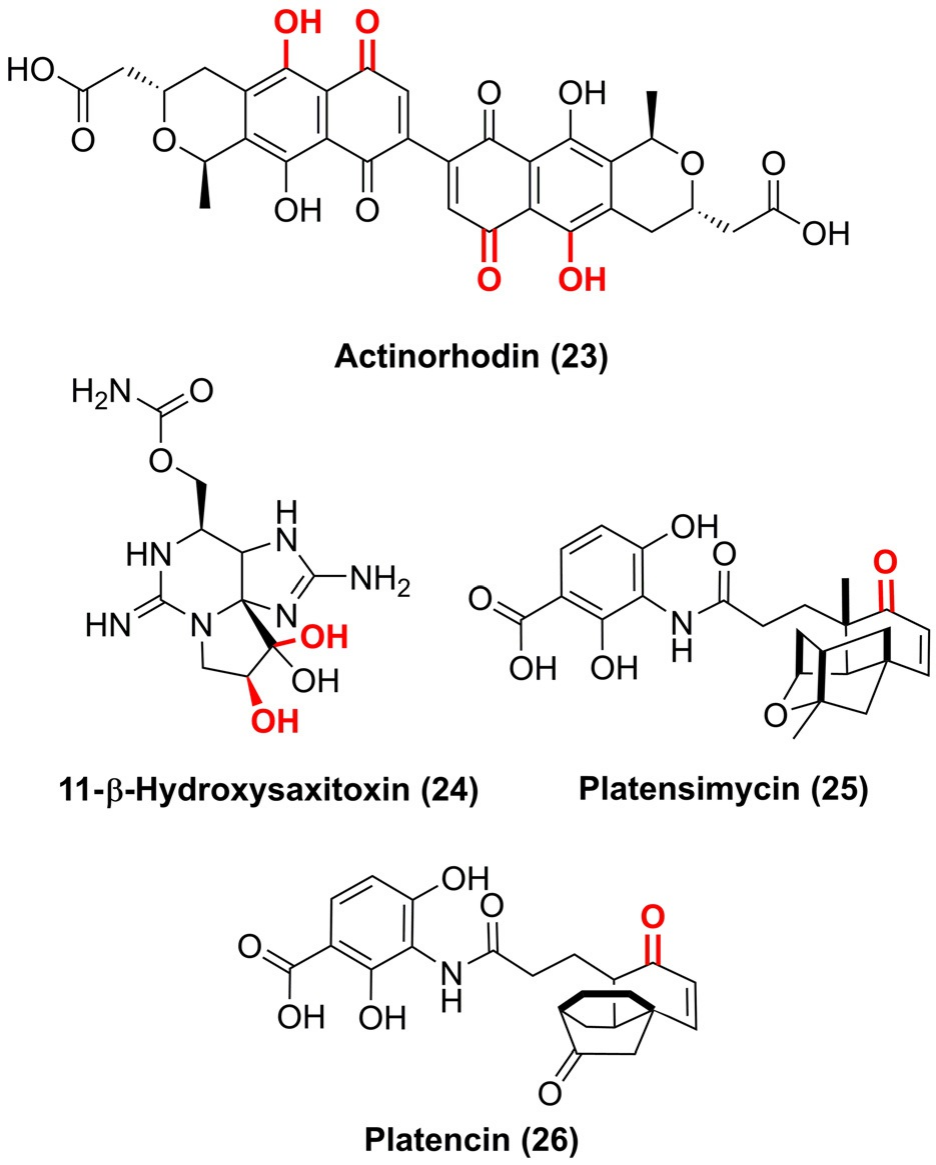
Nicht‐Häm Oxygenase‐katalysierte Hydroxylierung innerhalb verschiedener Biosynthesewege: FMO katalysiert zwei Hydroxylierungen in der Synthese des Pyranonaphthochinon‐Antibiotikums Actinorhodin (**23**) vor der Dimerisierung. Zwei Rieske‐Oxygenasen eröffnen die Reaktion von β‐Saxitoxinol zu 11‐β‐Hydroxysaxitoxin (**24**). Eine Nicht‐Häm‐di‐Eisen‐Monooxygenase hydroxyliert Platensimycin (**25**) und Platencin (**26**) vor deren weiterer Oxidation.

Hydroxylierungsreaktionen im Bereich der späten Funktionalisierungen werden ebenso von Nicht‐Häm‐Oxygenasen aus Biosynthesewegen, wie z. B. FMOs, Rieske‐Oxygenasen, Di‐Eisen‐Oxygenasen und Fe/αKG‐abhängigen Hydroxylasen, katalysiert. FMO‐katalysierte chemoenzymatische Synthesen lieferten beispielsweise ein vielfältiges Set aus stereodivergenten Azaphilon‐Naturstoffen.^[63]^ Innerhalb der Biosynthese des polyaromatischen Pyranonaphthochinon‐Antibiotikums Actinorhodin (**23**) fanden zwei aufeinanderfolgende Hydroxylierungen in C6‐ und C8‐Position mittels einer Zwei‐Komponenten‐FMO statt (Abbildung 8).^[64]^ Innerhalb des Naturstoffsynthesewegs zu Saxitoxin wurde kürzlich gezeigt, dass sequenzielle Dihydroxylierungen durch zwei Rieske‐Oxygenasen katalysiert werden. Jedes Enzym ist für eine selektive Hydroxylierung von β‐Saxitoxinol zu 11‐β‐Hydroxysaxitoxin (**24**) verantwortlich.^[65]^ Außerdem ist eine Nicht‐Häm‐di‐Eisen‐Monooxygenase an den Biosynthesen von Platensimycin (**25**) und Platencin (**26**) beteiligt, die in der C5‐β‐Position eine mühelose Diversifizierung der genannten Naturstoffe erlaubt.^[66]^ Insgesamt hebt die Breite an verschiedenen Oxygenasen und Substanzklassen, die enzymatisch hydroxyliert werden könnnen, die immense Bedeutung der späten Oxyfunktionalisierung in der Natur hervor.

### 2.2 Epoxidierung

Die späte Oxyfunktionalisierung von Kohlenstoff‐Kohlenstoff‐Doppelbindungen zu Epoxiden liefert ein wichtiges Motiv in vielen Naturstoffen. Trotzdem stellt sich die Chemoselektivität der Epoxidierung gegenüber der alternativen Hydroxylierung oftmals als eine Herausforderung dar. Hervorzuheben ist die späte Epoxidierung des Terpenoids β‐Cembrendiol (**27**), in der eine P450‐BM3‐Variante hohe Regio‐, Chemo‐ und Stereoselektivität lieferte (Schema 4).^[67]^ Innerhalb des 14‐gliedrigen Makrozyklus existieren drei potenzielle Positionen zur Epoxidierung, sodass die Kontrolle der Regioselektivität ein Problem bieten kann.^[67]^ Später wurde die Mutante P450‐BM3 V78A/F87A als Ausgangsvariante in einer rationalen Optimierungskampagne eingesetzt. Bindungsdichte‐Oberflächenkarten wurden dabei genutzt, um Aminosäurereste zu identifizieren, die verschiedene Bindungsmodi (de‐)stabilisieren und damit die C7,C8‐Epoxidierung gegenüber alternativen Hydroxylierungspositionen begünstigen.^[68]^ Systematische Substratoptimierung für 14‐gliedrige Cembranoide lieferte ein besseres Verständnis der regioselektiven Oxidation mittels P450‐BM3. Die Untersuchung verdeutlichte, dass die Ringrigidität zusammen mit dirigierenden Gruppen die Regioselektivität stark beeinflusst. Rekonstruktion der aktiven Tasche verlief erfolgreich, sodass die Regioselektivität für die Oxidation des Cembranoids abgestimmt werden konnte.^[69]^

**Scheme 4:**
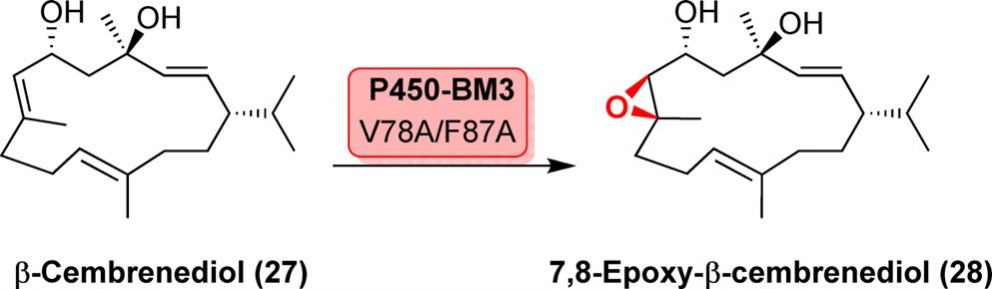
Epoxidierung des 14‐gliedrigen Makrozyklus β‐Cembrendiol (**27**) zu 7,8‐Epoxy‐β‐cembrendiol (**28**).

**Figure 9:**
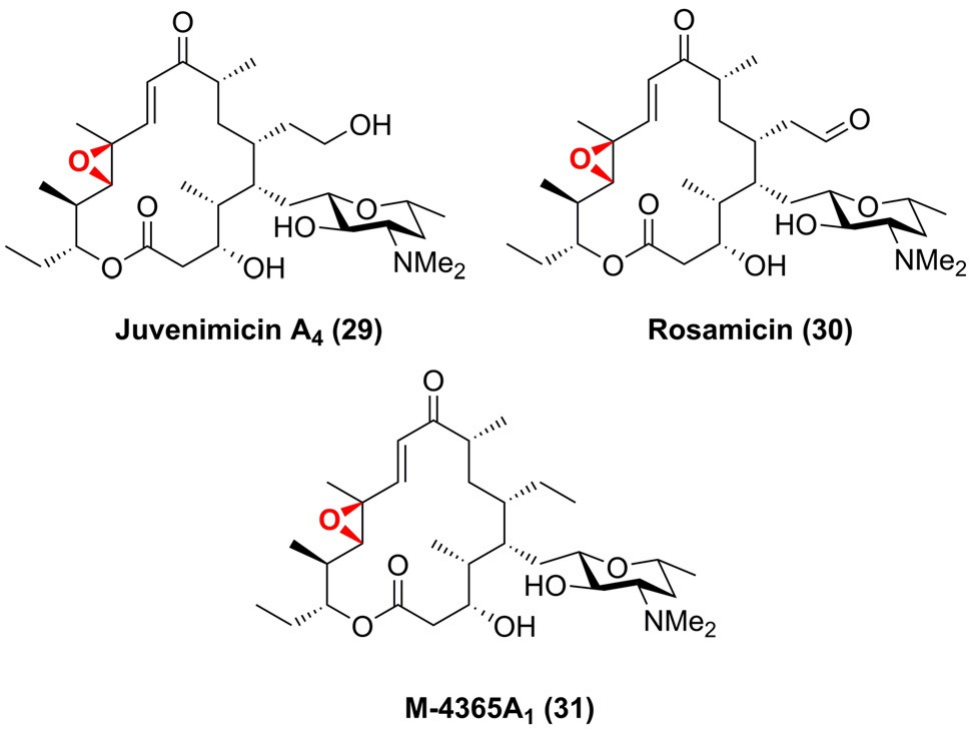
Epoxidierung der Tylacton‐basierten Makrolid‐Antibiotika Juvenimicin (**29**), Rosamicin (**30**) und M‐4365 (**31**), die durch die P450 JuvD katalysiert wird.

Durch Kombination aus Engineering und In‐vivo‐Synthese wurde die C12,C13‐Epoxidierung zu den Tylacton‐ähnlichen Makrolid‐Antibiotika Juvenimicin (**29**), Rosamicin (**30**) und M‐4365 (**31**) ermöglicht, indem ein artifizielles chimäres Konstrukt aus JuvD und einer Reduktase in die Biosynthesewege eingeführt wurde (Abbildung 9).^[70]^

In Ergänzung zu P450‐Enzymen sind UPOs und FMOs wichtige Biokatalysatoren für Epoxidierungsreaktionen. Vor kurzem wurde eine UPO (*Cgl*UPO) identifiziert, welche die Bildung des 4,5β‐Epoxids (**32**) aus **14** katalysiert (Schema 5).^[71]^

**Scheme 5:**
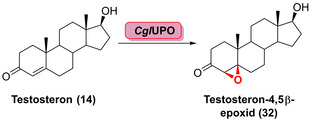
Stereoselektive UPO‐katalysierte Epoxidierung des Steroids Testosteron (**14**).

*Cgl*UPO zeigte eine hohe Chemo‐ und Stereoselektivität, sodass die Epoxidierung gegenüber der 16α‐Hydroxylierung bevorzugt wurde. UPOs bieten eine Alternative zu den besser etablierten P450‐Enzymen. Jedoch stellt die geringe Toleranz des Cosubstrats H_2_O_2_ einen beachtenswerten Nachteil dar, was ausgeklügelte In‐situ‐Regenerationssysteme erforderlich macht. Im Gegensatz zu P450‐Enzymen sind UPOs in späten Funktionalisierungen komplexer Moleküle deutlich weniger untersucht.

### 2.3 Spirozyklisierung

Das Spiro‐Motiv ist aufgrund seiner rigiden Konformation, die Ligand‐Rezeptor‐Interaktionen begünstigen kann, besonders attraktiv in der Wirkstoffentwicklung.^[72]^ Die Bedeutung spirozyklischer Verbindungen in Naturstoffwegen wurde von Tang et al. 2017 in einem Übersichtsartikel veröffentlicht.^[24]^ Es sollte erwähnt werden, dass die biokatalytische Spirozyklisierung bislang auf wenige außergewöhnliche Beispiele begrenzt ist, die lediglich aus Naturstoffsynthesewegen stammen und im Folgenden diskutiert werden.

**Figure 10:**
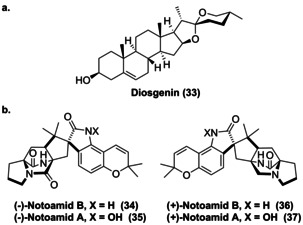
Die Spiroketal‐Naturstoffe Diosgenin (**33**) und Notoamid‐Derivate (**34**–**37**), in welchen die Spirozyklisierung durch Oxyfunktionalisierungen mittels P450‐Enzymen bzw. FMOs katalysiert wird.

Die Dihydroxylierung von Cholesterin zum 5,6‐Furoketal des Cholesterins wird durch zwei P450‐Enyzme, *Pp*CYP90G4 und *Tf*CYP90B50, katalysiert, welche die weiterführende Oxidation erlauben und schließlich das Spiroketal Diosgenin (**33**) produzieren (Abbildung 10 a).^[73]^

Außerdem weisen die antikanzerogenen Substanzen (+)/(−)‐Notoamid A/B (**34**–**37**) eine Spiro‐Oxindol‐Gruppe auf, die biokatalytisch mittels zweier FMOs (NotI und NotI′) durch sequenzielle stereoselektive Epoxidierung und *semi*‐Pinakol‐Umlagerung gebildet wird (Abbildung 10 b).^[74]^

Die beiden Fe/αKG‐Hydroxylasen (SptF and SptN) wurden kürzlich im Gencluster des Meroterpenoids Emeridon F aus *Apergillus* sp. TJ23 identifiziert.^[75]^ Beide Dioxygenasen zeigen verschiedene Oxyfunktionalisierungen, wobei SptF eine oxidative Umlagerung katalysiert, der eine Epoxidierung vorangeht, während SptN die regio‐ und stereoselektive Hydroxylierung in C9‐Position des Kernmotivs von Emeridon F (**39**) katalysiert (Schema 6). Es lässt sich vermuten, dass diese Enzyme Teil des Biosynthesewegs des spirozyklischen Emeridon‐Analogs Spiroaspertrion A (**40**) sind.^[75]^ In diesem Zusammenhang ist interessant, dass SptF und SptN auch In‐vitro‐Aktivität gegenüber verschiedenen Emeridon‐Derivaten zeigen, was somit die erste potenzielle Anwendung in der späten Funktionalisierung demonstriert.^[75]^

**Scheme 6:**
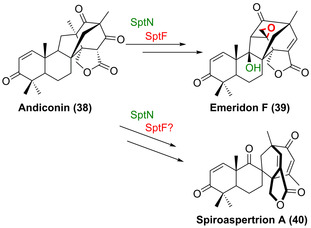
Fe/αKG‐Hydroxylase‐katalysierte Hydroxylierung und Epoxidierung als Teil des Biosynthesewegs von Emeridon F (**39**) aus Andiconin (**38**) und ihre mutmaßliche Beteiligung innerhalb des Synthesewegs zum Spiro‐Meroterpenoid Spiroaspertrion A (**40**).

### 2.4 Desoxyfluorierung

Die Oxyfunktionalisierung bietet die Möglichkeit zum Einbau von Fluor via Desoxyfluorierung. Als alternativer Ansatz wird die direkte Fluorierung durch Biohalogenierungsreaktionen ausführlich in Abschnitt 3.4 behandelt.

**Figure 11:**
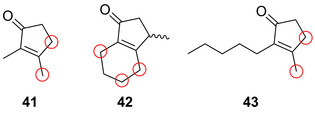
Regioselektive enzymatische Fluorierung der Cyclopentenon‐Derivate (**41**–**43**) durch eine von einer P450‐BM3‐Variante katalysierte Hydroxylierung und anschließende Desoxyfluorierung (rote Kreise: Diversifizierungspositionen).

Die biokatalytische regio‐ und stereoselektive Hydroxylierung pharmazeutisch relevanter Bausteine macht die weitere Modifizierung durch einen chemischen Fluorierungsschritt möglich. Ein entsprechendes chemoenzymatisches Verfahren wurde von Rentmeister et al. entwickelt.^[76]^ Für die selektive Zwei‐Schritt‐Fluorierung niedermolekularer organischer Verbindungen katalysiert eine optimierte P450‐BM3‐Variante zunächst eine selektive Hydroxylierung, wobei die Cyclopentenon‐Derivate (**41**–**43**) in zwei bis drei verschiedenen Positionen hydroxyliert wurden (Abbildung 11). Nach Isolierung des hydroxylierten Produkts konnte schließlich die Desoxyfluorierung mithilfe von Diethylaminoschwefeltrifluorid (DAST) ausgeführt werden (Schema 7 a). Zur Erweiterung des Konzepts wurde ebenfalls gezeigt, dass die Transformation einer Methoxygruppe in einen Fluorsubstituenten möglich ist. Nach vorangehender Hydroxylierung und dem daraus resultierenden Zerfall des Halbacetals in das entsprechende hydroxylierte Intermediat (**48**) erfolgte die Fluorierung im finalen Schritt (Schema 7 b).

**Scheme 7:**
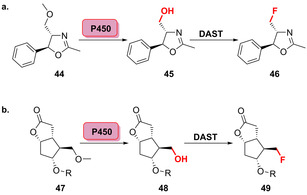
Regioselektive chemoenzymatische Transformation der Methoxygruppe in einen Fluorsubstituenten, gezeigt für a) 5‐Phenyloxazolin‐Derivate (**44**) und b) Corey‐Lacton‐Derivate (**47**).

Außerdem wurde die Desoxyfluorierung auf sterisch anspruchsvolle Terpenoide angewendet, z. B. für die Sesquiterpenlacton‐Derivate (7*R*)‐Fluorartemether (**52**) und (7*R*)‐Fluorartersunat (**53**).^[40]^ Die zuvor erwähnten optimierten P450‐BM3‐Varianten wurden eingesetzt, um die regio‐ und stereoselektive Hydroxylierung von Artemisin (**2**), gefolgt von der Desoxyfluorierung, auszuführen (Schema 8). Die Anwendung der biokatalytisch induzierten Desoxyfluorierung erweitert das Potenzial oxidativer Enzyme für die Synthese fluorierter Wirkstoffderivate. P450‐Enzyme ermöglichen die stereoselektive Heterofunktionalisierung komplexer Molekülgerüste und bieten daher eine nützliche Alternative zu den deutlich seltener vorkommenden Fluorinasen (Abschnitt 3.4).

**Scheme 8:**
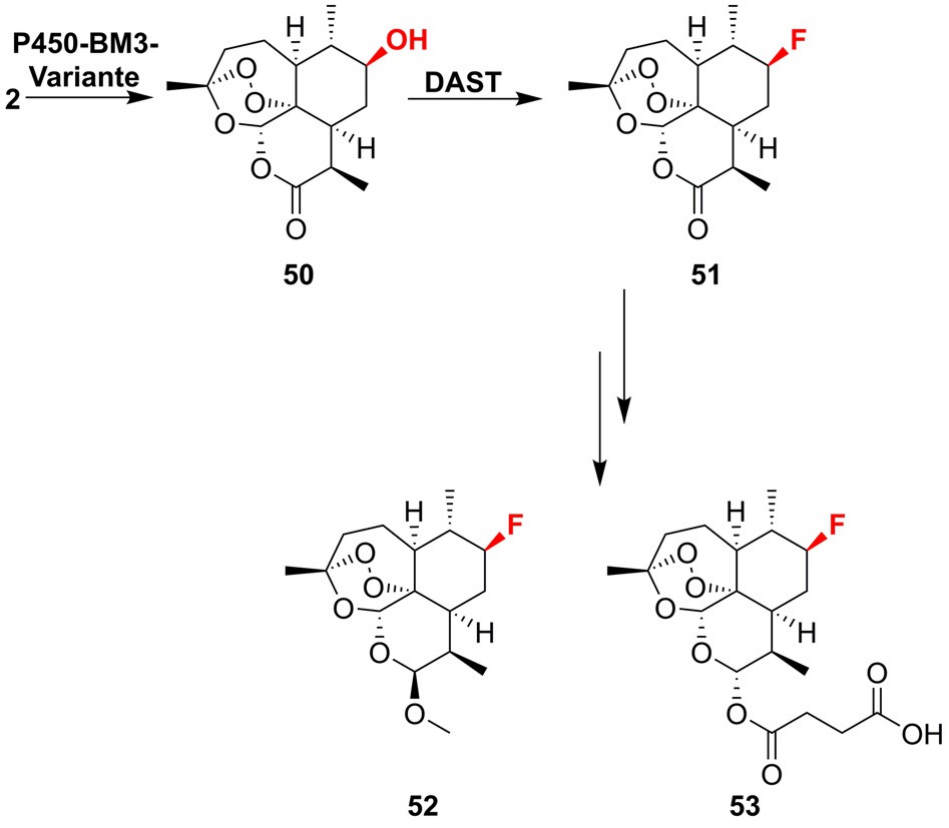
Chemoenzymatische Desoxyfluorierung von Artemisin (**2**) ermöglicht die weitere Reduktion zu (7*R*)‐Fluorartemether (**52**) sowie (7*R*)‐Fluoroartesunat (**53**).

### 2.5 Nicht‐natürliche Oxyfunktionalisierungen

Nicht‐natürliche Oxyfunktionalisierungen stellen ein sich zunehmend entwickelndes Feld dar, das optimierte Biokatalysatoren für nicht‐natürliche Reaktionen ausnutzt. Hochaktuelle Beispiele sind z. B. Carbentransfer,[^77^, ^78^, ^79^, ^80^, ^81^, ^82^, ^83^] C‐H‐Aminierung,[^84^, ^85^, ^86^, ^87^] S‐H‐Sulfimidierung,^[88]^ Si‐H‐Hydroxylierung^[89]^ und die Aziridinierung.^[90]^ Diese für Enzyme neuartigen Funktionalisierungen, die vorwiegend von P450‐Enzymvarianten bewerkstelligt werden, wurden in aktuellen Übersichtsartikeln zur Optimierung und Anwendung im Detail beschrieben.[^91^, ^92^, ^93^] Ein typisches Merkmal der nicht‐natürlichen Enzymaktivitäten ist die Mutation des konservierten proximalen Cysteins zu einem Serin‐Rest in P450‐BM3. Infolgedessen steigt das Reduktionspotenzial des Ferryl‐Komplexes, und aufgrund der daraus resultierenden veränderten spektroskopischen Eigenschaften werden solche Enzyme als P411‐Enzyme bezeichnet.^[78]^

In einer kürzlich erschienenen Studie wurde die enantioselektive Cyclopropenierung interner Alkine vorgestellt. Eine evolvierte P411‐Variante war in der Lage, die Cyclopropenierung für eine Reihe von Alkin‐Substraten (**54**) zu katalysieren. Ein hoher Grad an Stereoselektivität (>99.9 % Enantiomerenüberschuss, *ee*) wurde mithilfe der hocheffizienten P411‐Variante (TTN ≤5760) für die resultierenden Cyclopropene (**56**) erreicht (Schema 9 a). Zudem konnten P411‐Varianten eine propargylische C‐H‐Insertion (**58**), Cyclopropanierung (**59**) oder [3+2]‐Cycloaddition (**60**) katalysieren (Schema 9 b).^[83]^ Enzymvarianten wurden kürzlich für die stereoselektive Lacton‐Carben‐Insertion entwickelt (Schema 10). Durch die Einführung dieser funktionellen Gruppe waren zahlreiche Derivate des Sequiterpenlactonamins (**61**–**74**) in hohen Enantio‐ und Diastereoselektivitäten zugänglich.^[82]^

**Scheme 9:**
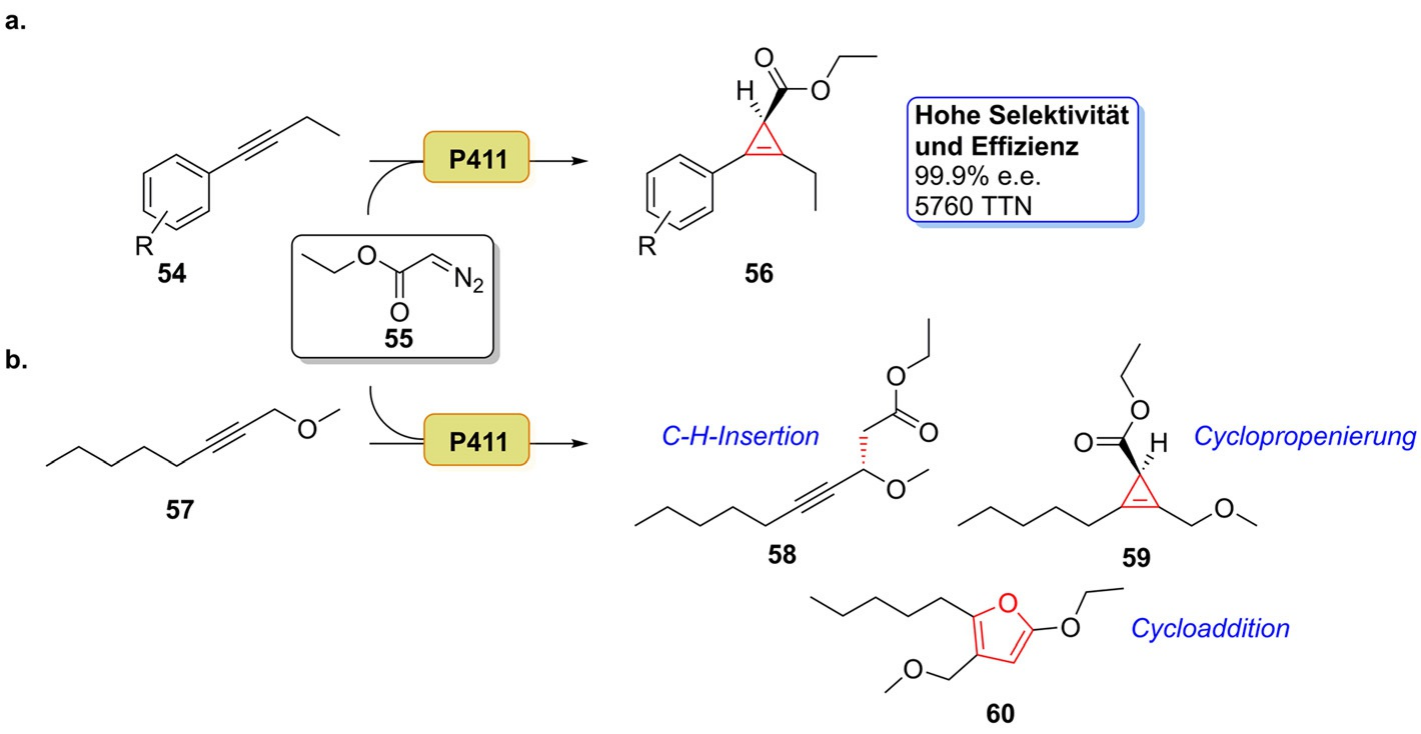
a) Enzymatische P411‐Varianten katalysieren Carbentransfer, der die Cyclopropenierung interner Alkine ermöglicht. b) Chemoselektive P411‐Varianten ermöglichen entweder die propargylische C‐H‐Insertion (**58**), eine Cyclopropenierung (**59**) oder eine [3+2]‐Cycloaddition (**60**).

**Scheme 10:**
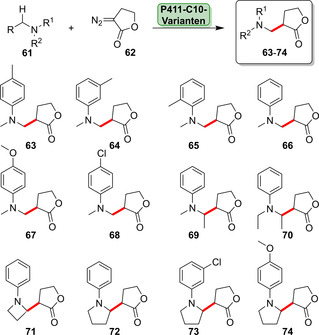
Spektrum der Lacton‐Carben‐Insertionen in primäre und sekundäre α‐Amino‐C‐H‐Bindungen durch optimierte Enzymvarianten, die von P411‐C10 abstammen. P411‐C10 wurde in der zuvor erwähnten Cyclopropenierung verwendet (Schema 9).

**Figure 12:**
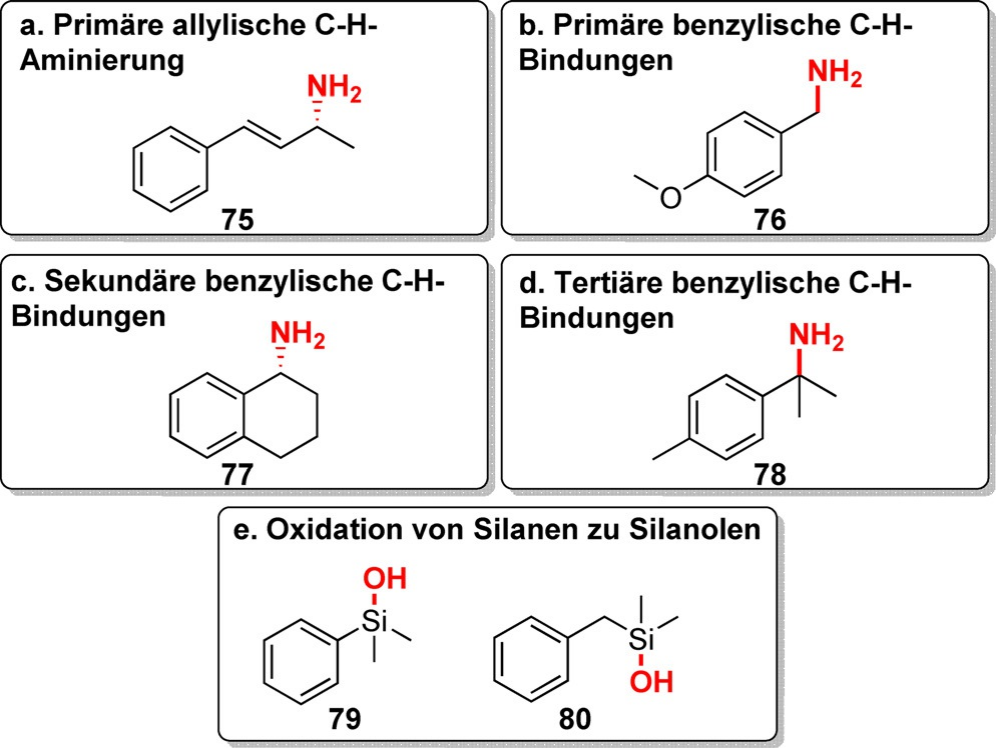
a–d) P411‐katalysierte primäre Aminierung allylischer und benzylischer C‐H‐Bindungen. e) Beispiele für die selektive Silan‐Oxidation durch Optimierung von P450‐BM3, was das Potenzial der Si‐basierten Chemie in der Biokatalyse zeigt.

Kürzlich wurde über die Konstruktion von P411‐Enzymen für die C(sp^3^)‐H‐Aminierung berichtet. Die hochgradig regio‐ und chemoselektive primäre Amininierung war in allylischen und benzylischen Positionen möglich (**75**–**78**, Abbildung 12 a–d).^[87]^ Somit haben P411‐Varianten eine Vielfalt an in der Natur bisher nicht beschriebenen Reaktionen für die späte Funktionalisierung eröffnet. Komplementäre Aktivität, Selektivität und Effizienz untermauern das große Potenzial für weiterführende Ansätze zur Funktionalisierung. In diesem Zusammenhang wurde gezeigt, dass P450‐BM3 in der Lage ist, Silane zu Silanolen zu hydroxylieren (**79**–**80**, Abbildung 12 e).^[89]^ Obwohl derzeit keine Silicium‐haltigen Wirkstoffe zugelassen sind,^[94]^ ist der Einbau von Silicium in Wirkstoffe als Bioisoster zum Kohlenstoffatom von zunehmendem Interesse.[^95^, ^96^]

Trotz des immensen Fortschritts und eines breiten Reaktionssets haben nicht‐natürliche Reaktionen bislang noch keinen Einzug in den Bereich der Wirkstoffentwicklung gefunden, da Studien zur Robustheit und einem ausgeweiteten Anwendungsspektrum noch ausstehen.

## 3 Biohalogenierung: Vielfältige Wege zum selektiven Aufbau der Kohlenstoff‐Halogen‐Bindung

### 3.1 Wie nutzen Halogenasen Halogenidsalze?

Die Halogenierung ist eine der häufigsten organischen Reaktionen und findet breite Anwendung in der Groß‐ und Feinchemikaliensynthese. Halogenatome üben oftmals einen günstigen Effekt auf die Wirksamkeit und pharmakokinetischen Eigenschaften von Wirkstoffen aus und sind zudem nützliche Mittel für eine Reihe von Derivatisierungen, wie z. B. Kreuzkupplungen.[^97^, ^98^] In der Tat enthielten im Zeitraum von 1914–2014 rund 30 % der zugelassenen Wirkstoffe ein Halogenatom.^[97]^

Halogenasen nutzen Halogenid‐Ionen und molekularen Sauerstoff oder Wasserstoffperoxid als Substrate und werden in vier Gruppen unterteilt: 1) Haloperoxidasen (Häm‐ oder Vanadium‐enthaltende Enzyme); 2) Flavin‐abhängige Halogenasen; 3) Nicht‐Häm‐Eisen/α‐Ketoglutarat‐abhängige Halogenasen;[^99^, ^100^] sowie 4) nucleophile Halogenasen (Fluorinasen).[^101^, ^102^] Oxidative Halogenasen bilden entweder formal eine “X^+^”‐Spezies (X=Cl, Br, I) oder ein Halogenradikal, wohingegen Fluorinasen ein nucleophiles Fluorid‐Ion übertragen (Schema 11). Für einen Überblick über die Fortschritte und aktuelle Anwendungen der halogenierenden Enzyme verweisen wir auf einen aktuellen Aufsatz von Minges und Sewald.^[103]^ In Ergänzung dazu sind weitere Details über die biokatalytische Halogenierung in Supporting Section 4 zusammengefasst (siehe Hintergrundinformationen). Da Haloperoxidasen aufgrund mangelnder Selektivität keine große Bedeutung in der späten Funktionalisierung haben, wird ihre Anwendung hier nicht weiter ausgeführt.^[104]^

**Scheme 11:**
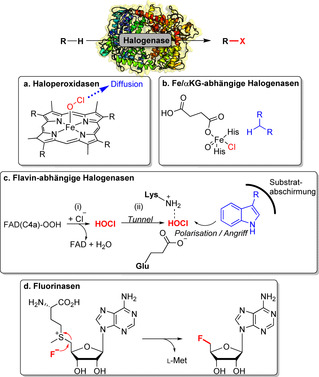
Reaktive Zwischenstufen für enzymatische Halogenierungen, gezeigt für verschiedene Halogenase‐Klassen. a) Haloperoxidasen: HOCl wird aus dem aktiven Zentrum freigesetzt. b) Fe/αKG‐abhängige Halogenasen: Die Nicht‐Häm‐Ferryl‐Oxo‐Spezies initiiert die Abstraktion eines Radikals vom Substrat. c) Flavin‐abhängige Halogenasen: Hypohalogenige Säure diffundiert durch einen Tunnel zum Substrat. d) Fluorinasen: In einer S_N_2‐Reaktion wird Fluorid als Nucleophil auf *S*‐Adenosylmethionin übertragen.

### 3.2 Flavin‐abhängige Halogenasen

Die herausragende Regioselektivität sowie die Möglichkeit, Reaktionen unter milden Reaktionsbedingungen durchzuführen, sind außergewöhnliche Charakteristika der Flavin‐abhängigen Halogenasen.[^105^, ^106^] Dennoch sind nicht alle Halogenasen synthetisch nutzbar, da einige Mitglieder dieser Klasse ein Carrier‐gebundenes statt eines freien Substrats benötigen.[^107^, ^108^] Zurzeit sind Tryptophan‐Halogenasen, die freies Tryptophan halogenieren, die am besten untersuchten Enzyme dieser Klasse. Der modulare Werkzeugkasten aus regiokomplementären Tryptophan‐Halogenasen hat sich im Laufe der Jahre ständig erweitert. In Anwesenheit von O_2_, einem Halogenid‐Salz und FADH_2_ wird selektiv entweder die C5‐, die C6‐ oder die C7‐Position des Indolrings von l‐Tryptophan (**81**) adressiert, und damit entsteht l‐Halogentryptophan (**82**) (Schema 12).[^109^, ^110^, ^111^, ^112^, ^113^, ^114^] Als Folge der inhärenten Sauerstoffempfindlichkeit von FADH_2_ muss der Cofaktor in situ durch eine Hilfsreaktion, z. B. mit einer Flavin‐Reduktase, zur Verfügung gestellt werden, um eine kontinuierliche Versorgung zu gewährleisten. Lichtgetriebene Reduktion des Flavins oder Nicotinamid‐Mimetika haben sich als nützliche Alternativen erwiesen.[^115^, ^116^] Ein bifunktionales Fusionsprotein bestehend aus Halogenase und Flavin‐Reduktase wurde ebenso generiert, um einen beschleunigten FADH_2_‐Austausch zwischen den katalytischen Zentren zu fördern.^[117]^ Jedoch wiesen die Konstrukte verschlechterte Produktausbeuten im Vergleich zu den separaten Enzymen auf.

**Scheme 12:**
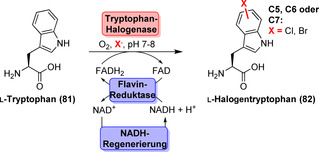
Regioselektive enzymatische Halogenierung von l‐Tryptophan (**81**) mit kontinuierlicher Cofaktorregenerierung.

**Figure 13:**
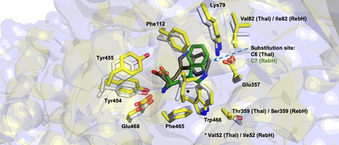
Die Überlagerung der aktiven Zentren von RebH (PDB‐ID: 2OA1) und Thal (PDB‐ID: 6H44) zeigt die modulare Regioselektivität der Halogenasen gegenüber l‐Tryptophan (**81**). Durch optimale Feinabstimmung sind die Indol‐Seitenketten in beiden aktiven Zentren im Vergleich koplanar zueinander orientiert, sodass entweder die C7‐ (RebH) oder die C6‐Position (Thal) in Richtung der katalytisch relevanten Reste, Lys79 und Glu357, ausgerichtet ist. Aminosäurereste mit abweichender Nummerierung zwischen RebH und Thal sind angezeigt, falls nötig. Tryptophan im Komplex mit Thal (dunkelgrau) und RebH (grün) ist als Stabmodell gezeigt; Seitenketten des aktiven Zentrums von Thal (C‐Atome: hellgrau) und RebH (C‐Atome: gelb) sind hervorgehoben (O‐Atome: rot, N‐Atome: blau).

Die Kristallstrukturen verschiedener komplementärer Enzyme zeigen eine starke Fixierung des Substrates im aktiven Zentrum, was ein gutes Beispiel für Katalysatorkontrolle darstellt, da die indolischen C‐H‐Positionen durch sterisch anspruchsvolle Reste des Proteins abgeschirmt werden und damit lediglich an einem Kohlenstoffatom des Indol‐Restes die elektrophile aromatische Substitution möglich ist (Abbildung 13).[^105^, ^118^, ^119^]

Tryptophan‐Halogenasen akzeptieren eine Reihe verschiedener substituierter Tryptophane und elektronenreiche Arene.[^120^, ^121^] Außerdem unternahm die Gruppe um Lewis umfangreiche Untersuchungen zum Substratspektrum verschiedener Halogenasen, wodurch sich ein deutlich breiteres Spektrum zeigte als ursprünglich angenommen.^[122]^

Frese und Sewald brachten die präparative Anwendung Flavin‐abhängiger Halogenasen voran, indem die Halogenase RebH mit den notwendigen Hilfsenzymen als vernetzte Enzymaggregate coimmobilisiert wurde.^[123]^ Ein Fluoreszenz‐Screening, das die Detektion von Bromtryptophan auf Basis eines Biaryls ermöglicht und die Suzuki‐Miyaura‐Kreuzkupplung als Reporterreaktion anwendet, vereinfacht das Enzym‐Engineering in der gerichteten Evolution.^[124]^ Minges et al. führten eine aufwändige Evolutionskampagne durch, in der randomisiertes und rationales Engineering kombiniert wurden, um Einflussfaktoren für die Thermostabilität und Aktivität zu untersuchen.^[125]^ Trotz vielfältiger Anstrengungen ist die Biohalogenierung bisher durch ihre geringe Effizienz begrenzt, die überwunden werden muss, um den Wert dieser Enzyme für die späte Funktionalisierung zu steigern.

Die späte Halogenierung anspruchsvoller biologisch aktiver Heterozyklen wurde durch einen Substratwanderungsansatz erreicht. Nach mehreren Runden der gerichteten Evolution und schrittweisen Modifizierungen der Substrate wurden sterisch anspruchsvolle Alkaloide und beispielsweise der β‐Blocker Carvedilol in die entsprechenden chlorierten Derivate (**83**–**88**) überführt (Schema 13).^[126]^ Ebenso erwies sich eine der Mutanten für die Desymmetrisierung von Derivaten des Methylendianilins (**89**) als nützlich, indem ein Stereozentrum weiter entfernt von der Halogenierungsposition aufgebaut wird, was auf konventionell chemischem Weg schwer erreichbar ist.^[127]^

**Scheme 13:**
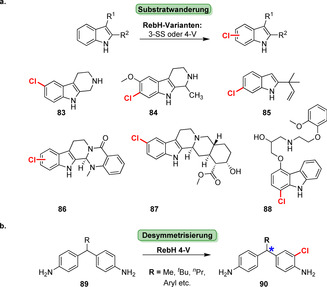
a) Späte Halogenierung verschiedener Indol‐abgeleiteter, sterisch anspruchsvoller Verbindungen (**83**–**88**) durch schrittweise gerichtete Evolution von RebH. b) Die enantioselektive Desymmetrisierung von Methylendianilinen (**89**) mithilfe von RebH 4‐V wird durch den Substituenten “R” ausgelöst (Stereozentrum: blauer Stern).

Ortega et al. identifizierten jüngst eine ungewöhnliche Tryptophan‐Halogenase, die an der Biosynthese eines 23‐mer Lanthipeptids beteiligt ist: MibH katalysiert die späte Halogenierung eines Trp‐Restes innerhalb eines peptidischen Biosynthesevorläufers (**91**), wodurch das Peptid NAI‐107 (**92**) resultiert (Schema 14).^[128]^ MibH ist hochgradig substratspezifisch, sodass selbst kleine Veränderungen des Peptidsubstrats nicht akzeptiert wurden und somit die Anwendung dieses Enzym in der Halogenierung von Peptiden eingeschränkt ist.

**Scheme 14:**
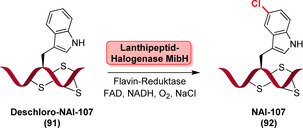
Späte Halogenierung eines Tryptophan‐Restes in der Lanthipeptid‐Vorstufe, was durch die nicht‐Carrier‐abhängige Tryptophan‐Halogenase MibH katalysiert wird. FADH_2_ wird mithilfe einer Flavin‐Reduktase bereitgestellt, die begleitend NADH oxidiert.

Während sich die überwiegende Zahl der Beispiele für die späte Funktionalisierung auf Tryptophan‐Halogenasen fokussiert, wurden ebenfalls Anstrengungen unternommen, um neue Halogenasen aus Genomdaten zu identifizieren. Dementsprechend wurden durch die Analyse von Sequenzähnlichkeitsnetzwerken 39 neue Halogenasen entdeckt.^[129]^ Ebenso führte dies zu der Identifizierung und genaueren Untersuchung von Halogenid‐spezifischen Halogenasen, wie z. B. Brominasen.[^130^, ^131^] Insbesondere Gkotsi et al. machten mit einer viralen Iodinase einen außerordentlichen Fund (Schema 15). Durch die Erstellung eines umfangreichen Substratprofils wurde eine deutliche Präferenz für die Iodierung deutlich.^[132]^ Diese Eigenschaft ist hochattraktiv für die C‐H‐Aktivierung, da Aryliodide wertvolle Ausgangssubstanzen für Kreuzkupplungsreaktionen sind. Dennoch bleibt die Frage ungeklärt, wie Brominasen oder Iodinasen vorzugsweise größere Halogenide akzeptieren und ob das Redoxpotenzial und/oder sterische Effekte entscheidende Faktoren zur Kontrolle der Halogenidselektivität sind.

**Scheme 15:**
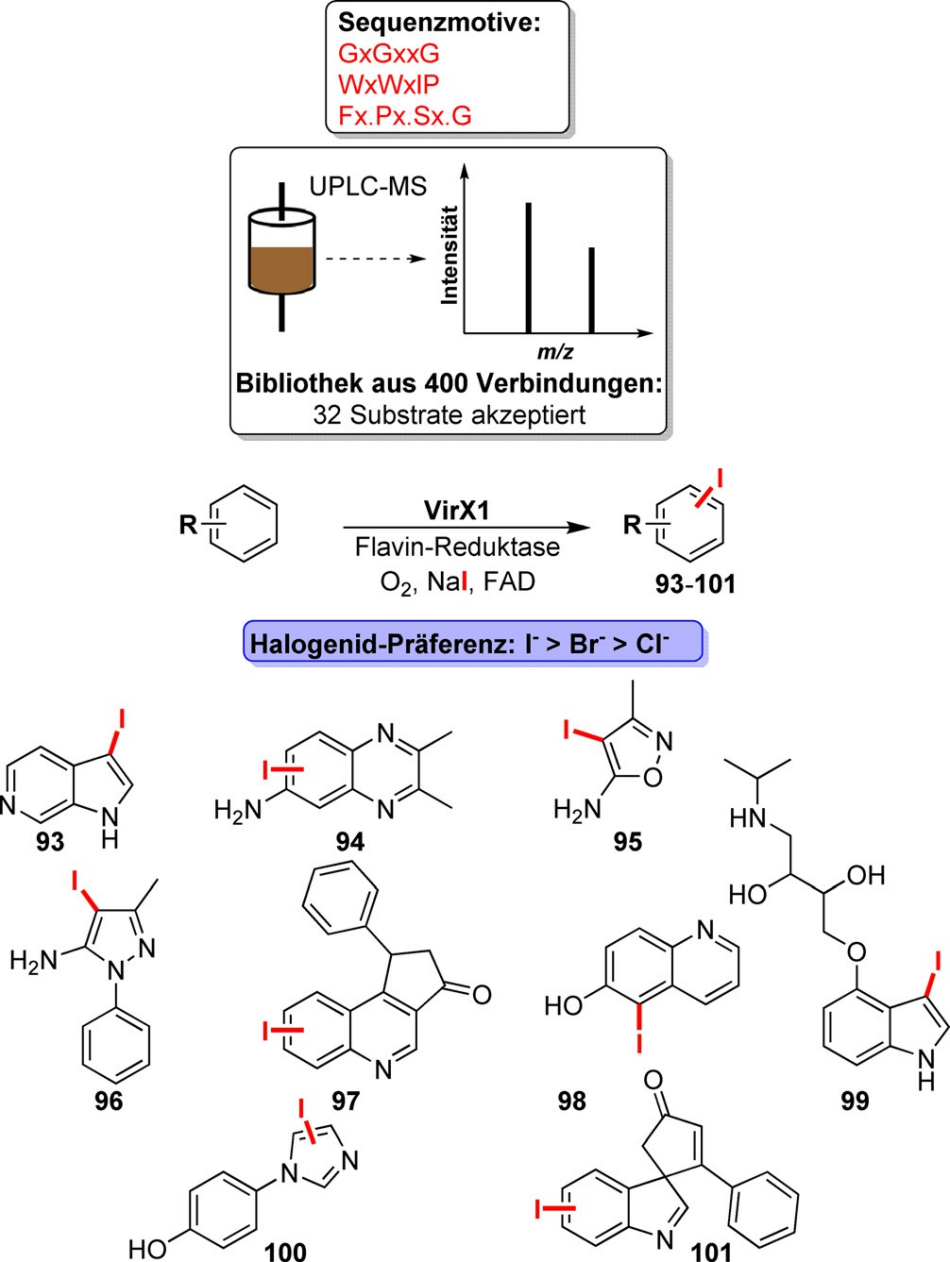
Bioinformatisches Halogenase‐Screening und Identifizierung einer Iodinase, die von Gkotsi et al. beschrieben wurde. Ein schrittweiser Arbeitsablauf ausgehend von der Sequenzidentifikation (Genome Mining) bis hin zur Erstellung eines Substratprofils brachte eine bis dato nicht beschriebene Iodinase hervor. Exemplarisch sind einige iodierte Reaktionsprodukte gezeigt.

### 3.3 Nicht‐Häm‐Eisen/α‐Ketoglutarat‐abhängige Halogenasen

Fe/αKG‐abhängige Halogenasen sind attraktive Werkzeuge, um weniger aktivierte C(sp^3^)‐H‐Reste zu aktivieren. Diese Eigenschaft wurde bislang nicht für Flavin‐abhängige Halogenasen oder Haloperoxidasen beschrieben.^[133]^ Die Fe/αKG‐abhängige Halogenase SyrB2 war die erste ausgiebig charakterisierte Halogenase dieses Typs.^[99]^ Obwohl ein Carrier‐gebundenes Substrat notwendig ist, wurde die Aktivität in vitro erfolgreich für die Halogenierung von Carrier‐gebundenem l‐Threonin (**102**) oder l‐Alloisoleucin nachgewiesen (Schema 16 a). Selbst Pseudohalogene wurden akzeptiert, sodass Azidierung und Nitrierung von **102** zu den Produkten **104** bzw. **105** möglich war.^[134]^

**Scheme 16:**
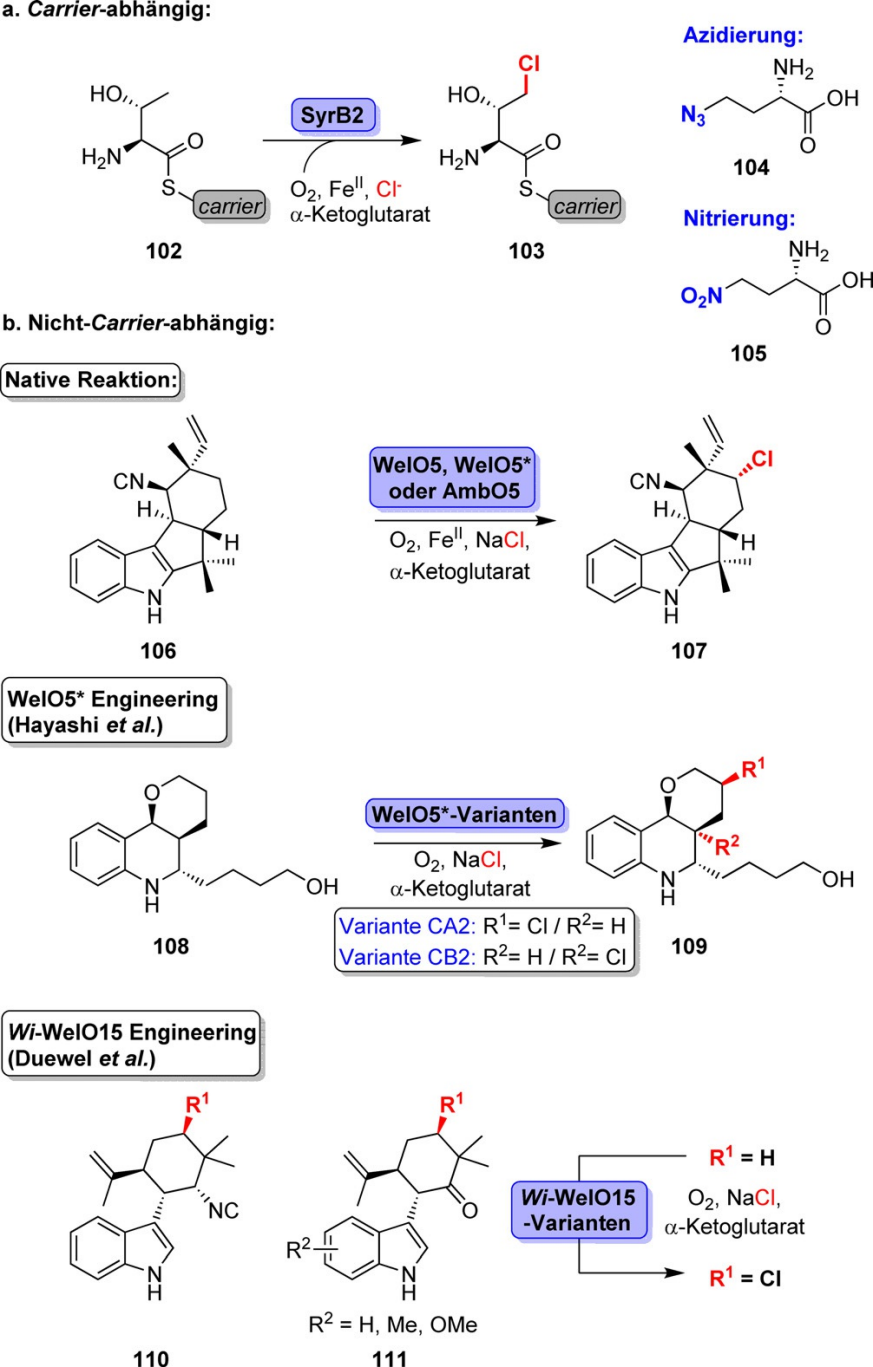
Repräsentative Beispiele für Reaktionen, die von Fe/αKG‐abhängigen Halogenasen für Carrier‐gebundene oder Carrier‐freie Substrate katalysiert werden.

Mit der Entdeckung einer Carrier‐unabhängigen Fe/αKG‐Halogenase stieg das Interesse an dieser Enzymklasse: WelO5 katalysiert die selektive Chlorierung von 12‐*epi*‐Fischer‐Indol U (**106**) in einem späten Stadium der Biosynthese.^[135]^ Das ist besonders überraschend vor dem Hintergrund der komplizierten Struktur sowie der zahlreichen ähnlichen C(sp^3^)‐H‐Positionen (Schema 16 b). Später wurden weitere Homologe identifiziert:^[135]^ AmbO5, WelO5* (oder *Hw*‐WelO15) und *Wi*‐WelO15 ermöglichen die Halogenierung strukturell wenig voneinander abweichender Fischer‐Indole und Hapalindole.[^136^, ^137^]

Das enge Substratspektrum der beschriebenen Enzyme ist ein entscheidendes Hindernis, das durch Protein‐Engineering angegangen wurde. Hayashi et al. beschrieben die erste Evolutionskampagne für eine Fe/αKG‐abhängige Halogenase mit dem Ziel, Nicht‐Indol‐Alkaloide zu halogenieren.^[138]^ Ein vom Martinellin abgeleitetes Fragment (**108**) diente als Modellsubstrat für die Evolution, ausgehend von einer schwachen promiskuitiven Aktivität von WelO5*. Struktur‐orientiertes Engineering führte schließlich zu den Varianten CA2 und CB2, die sich in der Regioselektivität unterscheiden und deutlich verbesserte katalytische Parameter für die Reaktion zum halogenierten Derivat (**109**) aufweisen. Ähnlich zum Wildtyp wies die Variante CA2 jedoch eine signifikante Hydroxylierungsaktivität auf. Erfreulicherweise wurde diese Nebenreaktion für die beste Variante CB2 minimiert und eine deutlich verbesserte Halogenierungsaktivität zum Derivat **109** festgestellt. Ebenso wurde über das Engineering von *Wi*‐WelO15 von Hoebenreich et al. berichtet. Die über vier Generationen evolvierten Enzymvarianten katalysierten die späte Chlorierung nicht‐natürlicher Hapalindol‐Derivate (**110**, **111**) im Milligramm‐Maßstab.^[139]^

Die jüngste Entdeckung von Fe/αKG‐abhängigen Aminosäure‐Halogenasen erweitert den synthetischen Nutzen dieser Enzymklasse deutlich. Die BesD‐Familie zeigt Aktivität gegenüber aliphatischen C‐H‐Gruppen verschiedener Aminosäuren (Schema 17).^[140]^ Bemerkenswert ist dabei, dass regioselektive Halogenierungen von Lysin und Ornithin zu den Verbindungen **112**–**114** sowie die von weiteren aliphatischen Aminosäuren, z. B. Leucin, Isoleucin und Norleucin, gelangen. Die Modifikation durch nachfolgende Enzyme erweiterte das Repertoir an zugänglichen halogenierten Aminosäurederivaten (**118**–**121**) zudem. Die Biohalogenierung freier aliphatischer Aminosäuren wurde bis dato nicht beschrieben und lässt sich nur schwierig mithilfe anderer Methoden umsetzen. Vor kurzem wurde außerdem die erste Nucleotid‐Halogenase veröffentlicht: Das Fe/αKG‐abhängige Enzym AdeV katalysiert die 2′‐Chlorierung eines Desoxyadenosin‐Restes und anderer Nucleotid‐Derivate, jedoch mit verringerter Effizienz.^[141]^

**Scheme 17:**
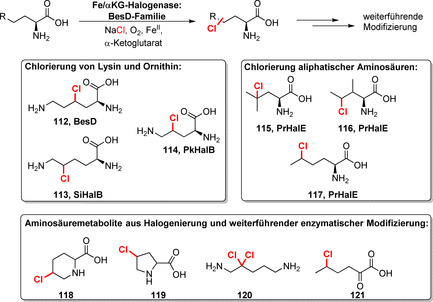
Halogenierung freier Aminosäuren durch Halogenasen der BesD‐Familie. Repräsentative Biotransformationsprodukte und die beteiligten Enzyme sind dargestellt.

Zweifellos ist die Anwendung von Fe/αKG‐Halogenasen mit Problemen verbunden. Unter anderem sind ein enges Substratspektrum und die bisher berichteten Reaktionen lediglich im analytischen oder geringen Milligramm‐Maßstab durchgeführt worden. Diese Faktoren beeinträchtigen die Bedeutung dieser Enzyme für die Biokatalyse. Selbst ausgeklügeltes Engineering hat sich bislang als schwierig erwiesen, um das Substratprofil drastisch zu verbreitern. Daher sind weitere Verbesserungen nötig: Allen voran muss sich das Set an Enzymen vergrößern, um diese zu attraktiven Katalysatoren für späte Funktionalisierungsreaktionen zu machen.

### 3.4 Fluorierende Enzyme

Organofluorverbindungen repräsentieren 38 % der zugelassenen, Halogen‐enthaltenden Wirkstoffe und sind damit nach Organochlorverbindugen am zweithäufigsten.^[97]^ Im Gegensatz dazu sind fluorierte Naturstoffe sehr selten, und ihre Biosynthesen beinhalten im Allgemeinen eine Adenosylfluorid‐Synthase, häufig auch Fluorinase genannt.^[142]^ Im Jahr 2002 wurde die erste Fluorinase im Bakterium *Streptomyces cattleya* entdeckt.^[143]^ Auch wenn später weitere homologe Enzyme folgten, ist das Spektrum bisher begrenzt.^[142]^ Generell katalysiert die Fluorinase den Austausch eines Chloratoms des 5′‐Chlor‐5′‐desoxyadenosins (5′‐ClDA, **122**) gegen l‐Methionin und generiert dabei *S*‐Adenosylmethionin (AdoMet, **123**). Eine anschließende S_N_2‐Reaktion ergibt den fluorierten Metaboliten 5′‐Fluor‐5′‐desoxyadenosin (5′‐FDA, **124**; Schema 18), was sich als ein mühsamer und wenig effizienter Weg herausstellt. Lowe et al. versuchten diesen Flaschenhals zu umgehen und die Nutzbarkeit der Fluorierung weiter auszuschöpfen, indem eine Finkelstein‐Reaktion angewendet wurde.^[144]^ In diesem Fall konnte **124** ausgehend von dessen 5′‐bromiertem Derivat (**125**) direkt in einem statt in zwei Schritten hergestellt werden.

**Scheme 18:**
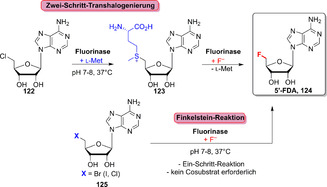
Heute angewendete Ansätze für den Zugang zu 5′‐FDA (**124**) durch eine Zwei‐Schritt‐Transhalogenierung oder eine Finkelstein‐Reaktion nach O′Hagan und Mitarbeitern.

Die [^18^F]‐Radiomarkierung bioaktiver Moleküle für die Positronen‐Emissions‐Tomographie (PET) ist ein Bereich, in dem Fluorinasen von Vorteil sind, um den ortsselektiven Einbau von [^18^F]‐Markern zu erzielen.^[145]^ Beispielsweise konnten sperrige Reste wie das zyklische Peptid c[RGDfK] über einen modifizierten Alkin‐Linker (**126**) angefügt werden, sodass die ^18^F‐Fluorierung des Derivats **127** möglich wurde (Schema 19).^[146]^ Ebenso wurde die Fluorinase‐katalysierte Transhalogenierung für das Antikörper‐Pretargeting sowie in der Markierung von Pharmakophoren von Prostatakrebstumoren eingesetzt.[^147^, ^148^]

**Scheme 19:**
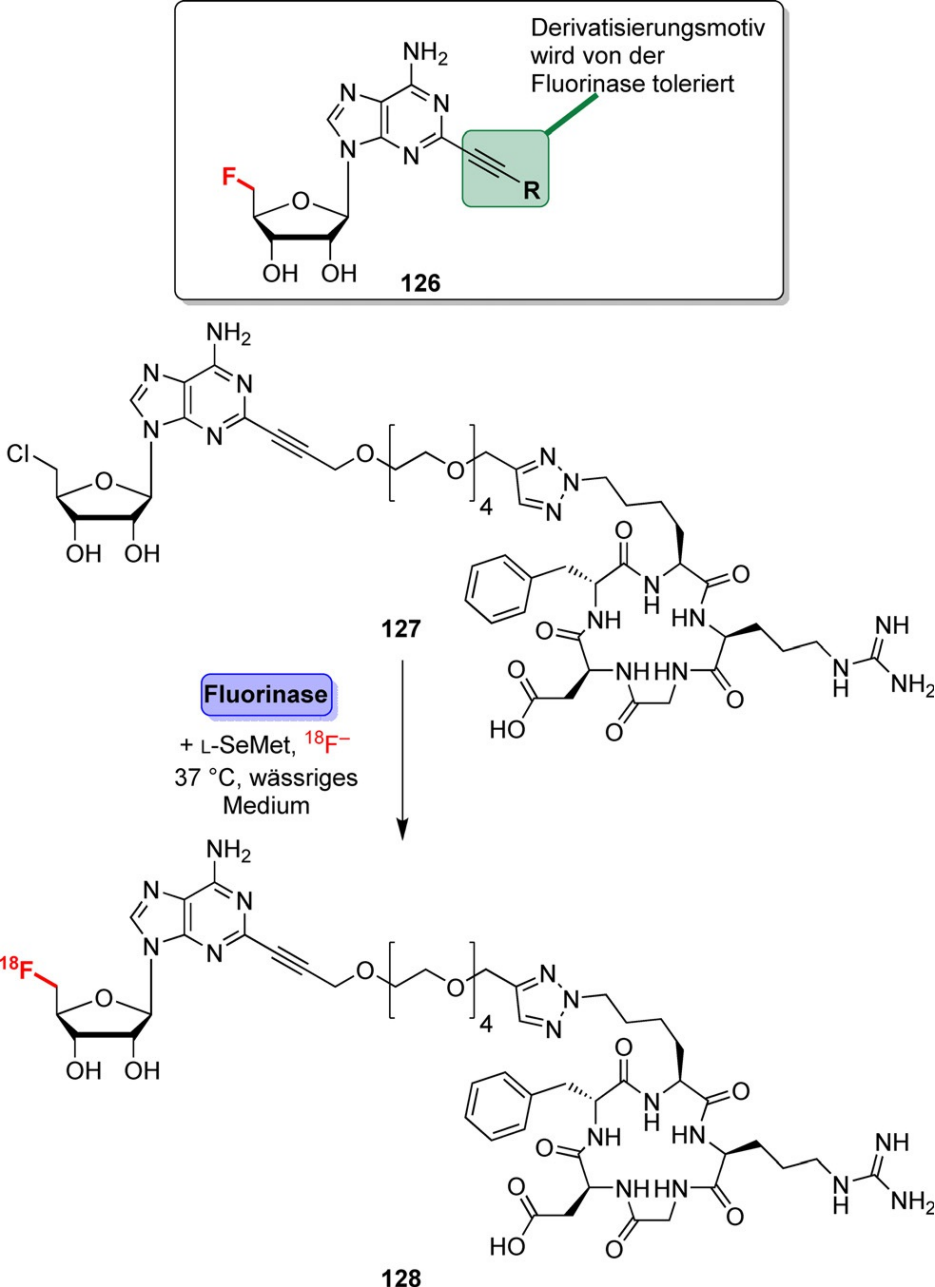
Biomoleküle können durch eine Alkin‐Gruppe verknüpft werden, während die Fluorierung weiterhin möglich ist, wie für die späte [^18^F]‐Markierung eines zyklischen RGD‐Peptidderivats (**127**) gezeigt. Die Fluorinase katalysiert den letzten Schritt zur Einführung des Radiomarkers durch Nutzung von [^18^F]‐Fluorid.

Trotz vorheriger Ansätze mit dem Ziel, die Fluorinase‐Effizienz durch Substratoptimierung und Reaktions‐Engineering zu steigern, ist die Nutzung von Fluorinasen in der späten Markierung aufgrund geringer kinetischer Effizienz und eines limitierten Substratspektrums leider wenig verbreitet. Auch Bemühungen, die Effizienz durch gerichtete Evolution zu erhöhen, führten zu mäßig verbesserten Varianten.[^149^, ^150^] Generell sind weitere Anstrengungen erforderlich, um die Substratspezifität zu lockern sowie die Synthesen der Cofaktor‐Analoga zu vereinfachen, damit die enzymatische Fluorierung ein zentrales Instrument für die pharmazeutische Synthese werden kann.

### 3.5 Aryl‐Diversifizierung durch Kombination der Biohalogenierung mit Kreuzkupplungsreaktionen

Die Kombination der Halogenierung mit Pd‐katalysierter Kreuzkupplung ist eine hervorragende Methode für die späte C‐C‐Bindungsbildung. Bio‐ und chemokatalytische Kaskadenprozesse sind hochattraktiv, da sie die hohe Spezifität von Enzymen mit der Vielfalt der Transformationen in der Chemokatalyse verbinden:^[151]^ Es wurde über Suzuki‐Miyaura‐Kreuzkupplungen, Mizoroki‐Heck‐ und Sonogashira‐Reaktionen berichtet, die gemeinsam mit der enzymkatalysierten Halogenierung im initialen Schritt eine Reihe substituierter Tryptophane zugänglich machten (**129**–**132**, Schema 20 a).[^152^, ^153^, ^154^, ^155^] Durch die Anwendung der zuvor erwähnten RebH‐Variante 4‐V konnten verschiedene bioaktive Arene (z. B. **88**, **135**) halogeniert und anschließend Kreuzkupplungen durchgeführt werden, wobei der Aufbau von C‐C‐, C‐N‐ und C‐O‐Bindungen demonstriert wurde (Schema 20 b).^[156]^

**Scheme 20:**
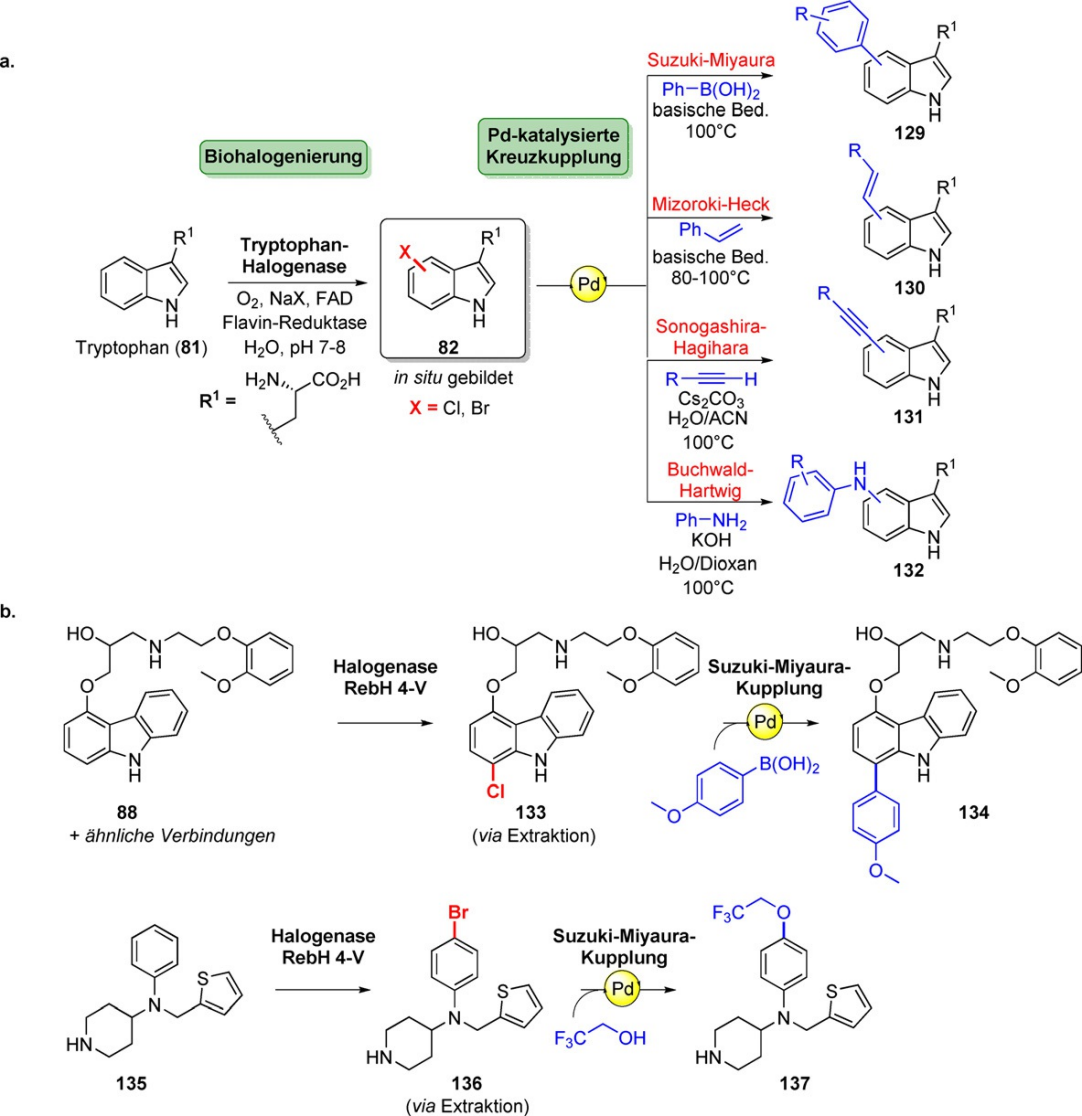
a) Pd‐katalysierte Kreuzkupplungen, verbunden mit der Biohalogenierung zur Diversifizierung von Tryptophan (**81**). b) Beispiele für späte Modifizierungsreaktionen mithilfe der Halogenase‐Variante RebH 4‐V und Suzuki‐Miyaura‐Kreuzkupplung.

Dachwitz et al. zeigten vor kurzem, dass Pd‐Nanopartikel das Potenzial für nützliche Kreuzkupplungskatalysatoren besitzen. Die Suzuki‐Reaktion konnte sowohl mit Bromtryptophanen als auch mit bromierten Peptiden in Wasser und an Luft unter milden Bedingungen durchgeführt werden, was eine wichtige Voraussetzung in chemoenzymatischen Kaskaden bietet, um Kompatibilitätsprobleme zu umgehen.^[157]^ Verschiedene Gruppen kombinierten erfolgreich enzymatische Halogenierung und Kreuzkupplung in einem chemogenetischen Ansatz. Dies erlaubte die Synthese von Aryl‐substituierten Naturstoffen durch den heterologen Einbau von Halogenasegenen in Biosynthesewege. Auf diese Weise konnten nicht‐natürliche Metabolite (**139**–**140**) erhalten werden (Schema 21).[^158^, ^159^, ^160^] Diese In‐vivo‐Ansätze umgehen Komplikationen, die häufig in vitro mit der Handhabung biosynthetischer Enzyme zur Herstellung komplexer Naturstoffe verbunden sind. Dennoch können die Gen‐Insertion und eventuell mangelnde Toleranz des extrinsischen Gens durch den Wirt mögliche Probleme verursachen. Oftmals stellt die Isolierung des modifizierten Naturstoffes aus dem Fermentationsmedium eine Herausforderung dar und resultiert in geringen Ausbeuten. Somit fehlt diesem Ansatz momentan ein breites Anwendungsspektrum, u. a. in der Erstellung Naturstoff‐abgeleiteter Wirkstoffbibliotheken.

**Scheme 21:**
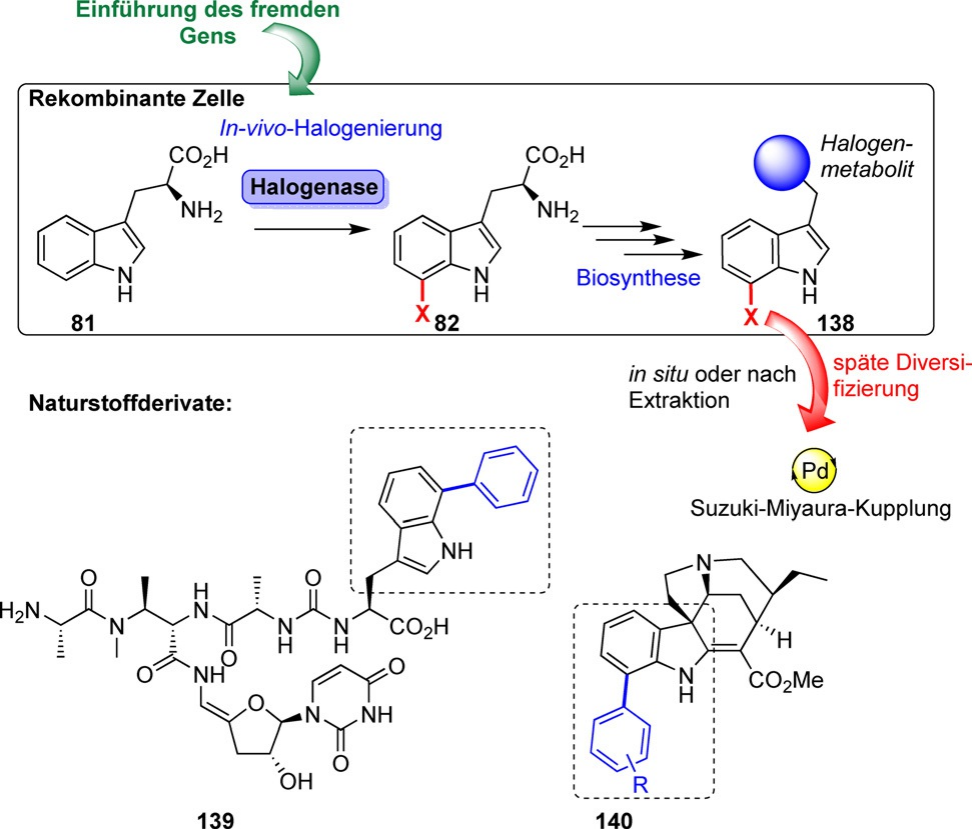
Ein chemogenetischer Ansatz kombiniert die Halogenierung von Tryptophan (**81**) mit der Biosynthese von Naturstoffen. Die resultierenden Naturstoffprodukte wurden final mittels Suzuki‐Miyaura‐Kupplung modifiziert. Somit ermöglicht die Biohalogenierung die Einführung von Diversität in komplexe Naturstoffe.

## 4 Späte Alkylierung und Acylierung

Der Aufbau von C‐C‐Bindungen ist fundamental für die Synthesechemie, um komplexe Molekülgerüste aufzubauen. Zahlreiche Methoden existieren dafür in der Chemie, angefangen von Aldolreaktionen bis hin zum weitläufigen Gebiet der Metallorganik. Im Gegenzug ist die Bandbreite solcher Transformationen in der Enzymkatalyse weniger weit entwickelt.^[161]^ Nichtsdestotrotz sind späte Funktionalisierungsreaktionen, die es ermöglichen, Kohlenstoffreste auf multifunktionelle Moleküle zu übertragen, essenziell in der Wirkstoffentwicklung.

### 4.1 Biokatalytische Übertragung von Methylgruppen und analogen Bausteinen

Eine bedeutsame Verbesserung von Rezeptor‐Bindungsaffinitäten kann durch die Methylierung einer Wirkstoff‐Leitstruktur beobachtet werden. Daher sprechen Medizinalchemiker auch vom “magischen Methyleffekt”.^[162]^ Der selektive Einbau von Methylgruppen in komplexe Moleküle ist oftmals eine Herausforderung und erfordert mehrere chemische Schritte sowie harsche Methylierungsreagenzien.^[163]^

In der Natur katalysieren *S*‐Adenosylmethionin‐abhängige Methyltransferasen (MTasen) die selektive Methylierung von Biopolymeren, z. B. von Nucleinsäuren, Proteinen oder Sekundärmetaboliten. Diese Fähigkeit hebt das große Potenzial der zielgerichteten enzymatischen Methylierung hervor.

AdoMet‐abhängige MTasen sind in der Lage, eine Methylgruppe von dem Donor AdoMet (**123**) auf eine Vielzahl von Nucleophilen (z. B. C, O, N, S, P) zu übertragen.^[164]^ In ersten Studien legte die Röntgenkristallstruktur der Catechol‐O‐Methyltransferase (COMT) die Grundlage für das Design entsprechender Varianten, die entweder *meta*‐ oder *para*‐methylierte Catechole produzieren und damit Bausteine für die Wirkstoffe Aliskiren und Mesopram bereitstellen.^[165]^

Dennoch besteht eine stetige Nachfrage nach robusten enzymatischen Methylierungsstrategien, denn das Donormolekül AdoMet (**123**) ist aufgrund seiner inhärenten Instabilität (*t*_1/2_=942 min bei pH 8.0 und 37 °C), aufwändigen Synthese und hohen Kosten ein Engpass.^[166]^ In der Vergangenheit wurde über attraktive Enzymkaskaden berichtet, welche die AdoMet‐Versorgung gewährleisten: Das Enzym SalL, das nativ als Fluorinase fungiert (siehe Abschnitt 3.5), erlaubt den Transfer von l‐Met auf 5′‐ClDA (**122**), wodurch Verbindung **123** gebildet wird (Schema 22).^[167]^ In Kombination mit einer MTase erlaubt die In‐situ‐Versorgung mit dem Donor **123** die Methylierung eines Substrat‐Nucleophils (**141**). Alternativ kann eine Methionin‐Adenosyltransferase (MAT) genutzt werden, um **123** aus ATP (**144**) und l‐Met zu generieren. Dieses Verfahren bietet den Vorteil, die AdoMet‐Erzeugung mit einem ATP‐Recyclingsystem zu kombinieren und die aufwändige Synthese von **122** zu umgehen.^[168]^

**Scheme 22:**
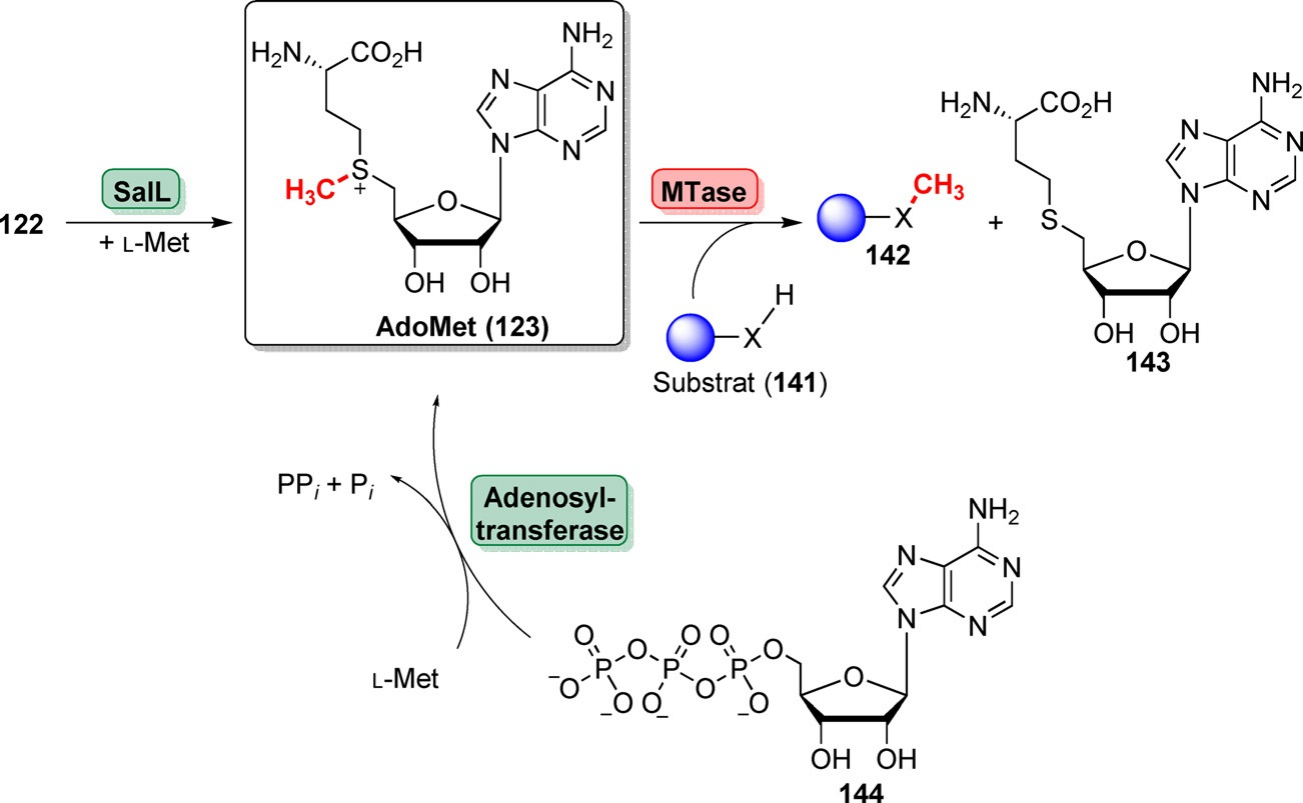
Enzymatische Ansätze zur Generierung des Methyldonors AdoMet (**123**), der für die Methylierung durch MTasen essenziell ist. SalL katalysiert die Substitution des 5′‐Chlorsubstituenten von **122** gegen l‐Met, sodass **126** gebildet wird. Alternativ können MATs genutzt werden, die aus ATP (**144**) und l‐Met den Donor **123** erzeugen.

Insbesondere AdoMet‐Analoga sind aufgrund höherer Cofaktor‐Lebensdauer und der Möglichkeit, andere Kohlenstoff‐Bausteine einzuführen, eine wichtige Errungenschaft für die späte Derivatisierung.^[169]^ Beispielsweise war in einer zweischrittigen Eintopf‐Reaktion eine NovO‐katalysierte C‐Methylierung möglich, wobei Methionin‐ (**145**) und ClDA‐Derivate (**146**) genutzt wurden, die von SalL akzeptiert wurden. Selbst die nicht‐natürliche Ethylierung war möglich, was die Flexibilität dieses breit untersuchten Systems hervorhebt (Schema 23).[^166^, ^170^, ^171^]

**Scheme 23:**
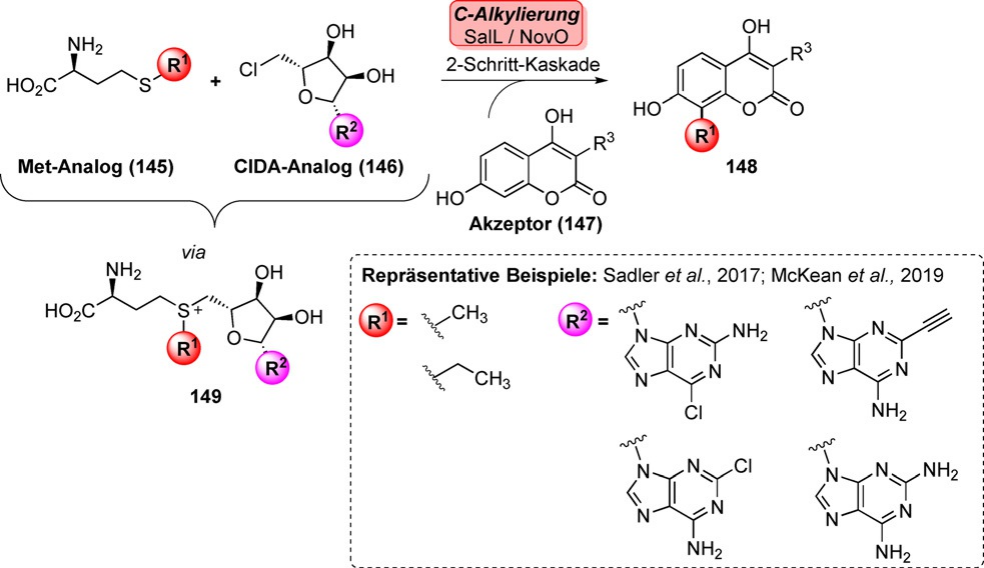
Synthese eines Sets aus AdoMet‐Analoga mithilfe von SalL ausgehend von Met‐ (**145**) und ClDA‐Derivaten (**146**). Die Modifikation der Nucleobase (R^2^) am Ribose‐Rest hat sich als nützlich erwiesen, um die Cofaktorstabilität zu steigern. Veränderung der Thioether‐Kette (R^1^) erlaubt den Einbau anderer Reste anstatt Methyl.

Die kürzlich beschriebene Carboxymethylierung erweitert das Spektrum möglicher Kohlenstoff‐Reste, die für die Dekorierung von Molekülgerüsten genutzt werden können:^[172]^ Das seltene AdoMet‐Derivat cxSAM (**152**) wird von der Synthase CmoA in situ aus Prephenat (**150**) und **123** hergestellt (Schema 24). Darüber hinaus wurden orthogonale MTAse‐Varianten konstruiert, die mit einer höheren Donorspezifität ausgestattet sind, damit die konkurrierende Methylierung unterdrückt wird und stattdessen die orthogonale Insertion des Carboxymethylrestes des Alkyldonors **152** in das Akzeptor‐Substrat (**153**) bewerkstelligt wird.

**Scheme 24:**
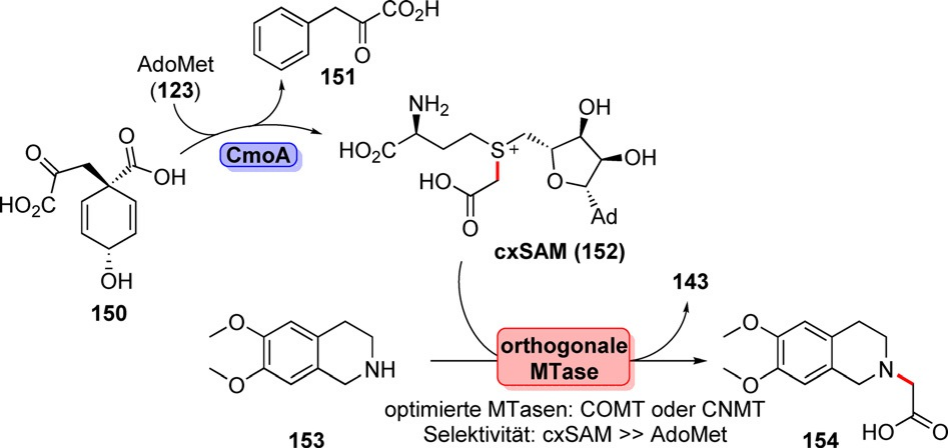
cxSAM (**152**) wird aus AdoMet (**123**) und Prephenat (**150**) mithilfe der Synthase CmoA erzeugt. Durch Engineering orthogonaler MTasen konnte schließlich eine selektive Carboxymethylierung erzielt werden.

Vor kurzem berichteten Liao und Seebeck über ein hochinnovatives System zum AdoMet‐Recycling, das die schlechte Atomökonomie und schwierige Mehrschritt‐Reaktionssysteme umgehen kann.^[173]^ Zentraler Bestandteil ist eine Halogenid‐Methyltransferase (HMT), die z. B. Iodmethan (MeI) als leicht verfügbare Alkylquelle nutzen kann (Schema 25). Die Autoren zeigten, dass HMT die exergonische Reaktion zwischen *S*‐Adenosylhomocystein (**143**) und MeI zum Donor **123** katalysieren und von MTasen in situ zur Methylierung genutzt werden kann. Die Anwendung in einer Kaskade machte β‐Methyl‐α‐aminosäuren zugänglich, indem eine Transaminase und eine MTase in Verbindung mit der HMT eingesetzt wurden.^[174]^ Dieser neuartige Ansatz zur AdoMet‐Generierung wurde in zwei aktuellen Studien erweitert: Durch die erfolgreiche Evolution der HMT wurden alternative Halogenalkane in Ergänzung zu MeI für enzymatische Alkylierungen zugänglich gemacht.^[175]^ Ebenso resultierten die N‐Methylierung, ‐ethylierung und ‐propylierung von Pyrazolen in außerordentlichen Regioselektivitäten durch die Anwendung optimierter MTasen. Dabei wurde eine promiskuitive HMT aus einem Pilz identifiziert, die neben Iodmethan auch andere Halogenalkane als Substrate umsetzt und damit die entsprechenden alkylierten Derivate des AdoMets generiert und auf das Pyrazol‐Gerüst überträgt.^[176]^

**Scheme 25:**
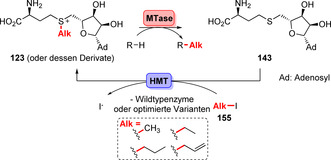
AdoMet‐Generierung durch Verwendung leicht verfügbarer Iodalkane (**155**), die von HMTs akzeptiert werden. Die MTase ermöglicht die direkte enzymkatalysierte Übertragung der Alkylgruppe durch das Donormolekül **123** oder seine Derivate.

Die vielfältige Alkylierung eines Rebeccamycin‐Derivats (**156**) gelang in einem als “Alkylrandomisierung” bezeichneten Ansatz. Dabei wurden 18 S‐ oder Se‐enthaltende Analoga des AdoMet kumulativ gebildet. Die Promiskuität des humanen Enzyms hMAT2 wurde ausgeschöpft, um acht AdoMet‐Analoga, u. a. Selen‐Derivate, zu erhalten, die von der MTase RebM akzeptiert wurden und die entsprechenden Derivate der Antitumor‐Substanz (**157**) lieferten (Schema 26).^[171]^

**Scheme 26:**
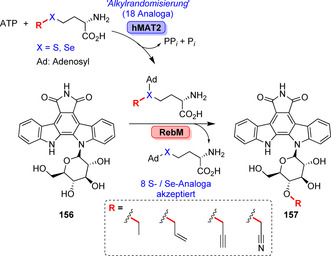
Späte Alkylierung des Indolcarbozols am Beispiel eines Rebeccamycin‐Derivats (**156**). Die “Alkylrandomisierung” kombinierte das humane Enzym hMAT2 mit der MTase, sodass vier Paare von S/Se‐Congeneren akzeptiert wurden und die entsprechenden Alkylderivate (**157**) entstanden.

Durch die Kombination einer Tyrosinase mit COMT wurde die späte Methylierung nicht‐geschützter Peptide ermöglicht.^[177]^ Die direkte Hydroxylierung eines Tyrosinrestes (**156**) als Teil einer Peptidsequenz erlaubte im Anschluss die Methylierung des neu eingeführten Hydroxyrestes in einem Eintopf‐Verfahren (Schema 27)

**Scheme 27:**
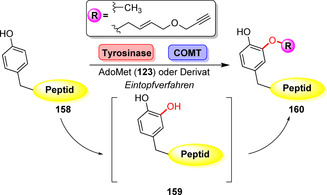
Peptid‐Alkoxylierung durch Kombination einer Tyrosinase mit COMT.

Vor kurzem wurde das rationale Engineering einer O‐MTase aus einem Pilz beschrieben, um die Regiospezifität in der Synthese der von Wirkstoffen abgeleiteten O‐methylierten Benzoldiollacton‐Polyketide zu modulieren.^[178]^ Ein nennenswerter Vorteil von In‐vivo‐Systemen ist die kontinuierliche Produktion von AdoMet (**123**). Dabei wurden z. B. *E.‐coli*‐Zellen als “Biofabriken” genutzt, damit Dopamin zu (*S*)‐Reticulin umgesetzt werden konnte. Dabei handelt es sich um ein Schlüsselintermediat in der Biosynthese pharmazeutisch relevanter Benzylisochinolinalkaloid‐Morphine und Codeine (Schema S1, Hintergrundinformationen).^[179]^ Dieser Pfad nutzte fünf mikrobielle bzw. pflanzliche Enzyme, u. a. drei verschiedene N‐ oder O‐MTasen. Ein anderes Beispiel ist das Engineering von *E.‐coli*‐Zellen, um *p*‐Cumarinsäure in das Flavonoid 7‐*O*‐Methylaromadendrin zu konvertieren, das anti‐inflammatorische und anti‐kanzerogene Aktivität besitzt.^[180]^

### 4.2 Friedel‐Crafts‐Alkylierung und ‐Acylierung

Die vor über einem Jahrhundert entdeckten Friedel‐Crafts‐Reaktionen haben sich zu nützlichen Werkzeugen für die Synthesechemie entwickelt, da sie die Möglichkeit bieten, C‐C‐Bindungen an Arenen aufzubauen.^[181]^ Vor kurzem wurde über biokatalytische Pendants dieser klassischen Reaktionen berichtet, die zahlreiche Vorteile bieten könnten, u. a. Regio‐ und Stereoselektivität, die häufig in der klassischen Friedel‐Crafts‐Synthese fehlen. Somit bietet dieses aufkommende Gebiet eine neue Alternative zur bislang weniger untersuchten Arylmodifizierung.

Ein aktuelles Beispiel für die Friedel‐Crafts‐Acylierung wurde von Kroutils Gruppe beschrieben. Eine bakterielle Mehrkomponenten‐Acyltransferase aus *Pseudomonas protegens* (PPATase) war zur regioselektiven C‐Acylierung von Phenolderivaten, z. B. elektronenreiche Resorcinol‐Derivate (**161**), in der Lage, indem typische Acyldonoren (z. B. **162**) eingesetzt wurden (Schema 28 a).^[182]^

**Scheme 28:**
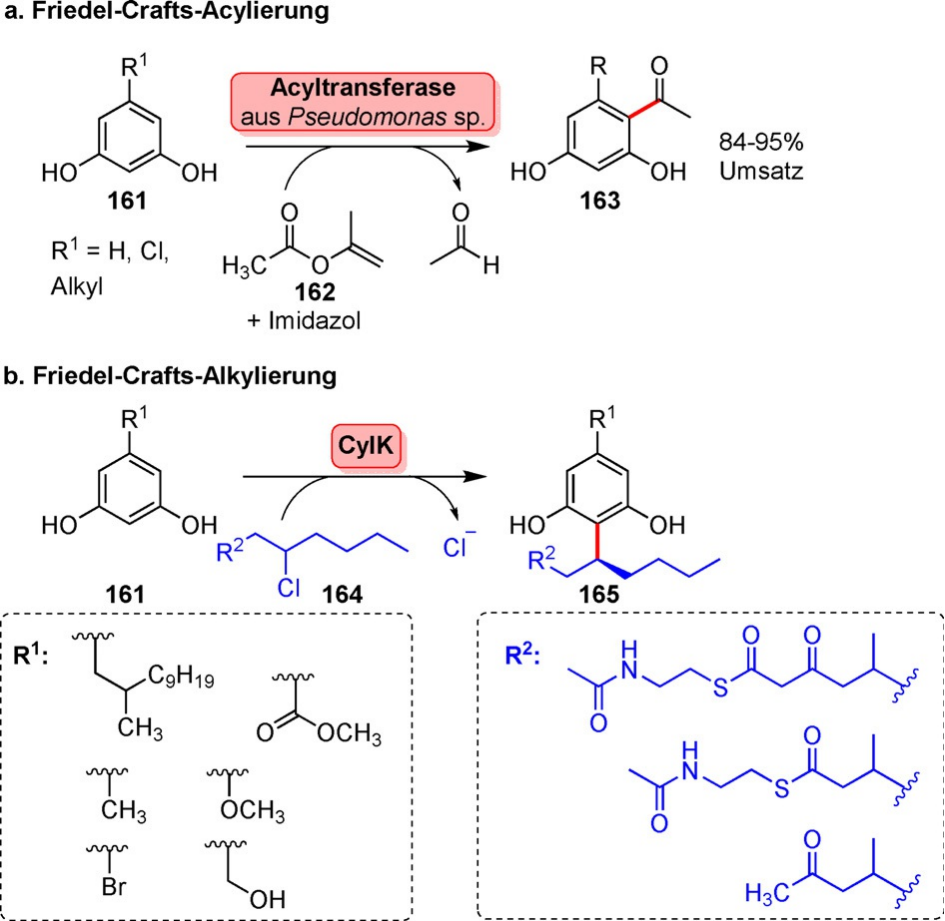
a) Friedel‐Crafts‐Acylierung, katalysiert von einer Acyltransferase. Die aktivierten Acyldonoren wurden erfolgreich auf Resorcinol (**161**) übertragen. b) Die Friedel‐Crafts‐Alkylierung wurde durch das C‐C‐kuppelnde Enzym CylK ermöglicht.

Ebenso wurde kürzlich ein Beispiel für die Friedel‐Crafts‐Alkylierung beschrieben. CylK, das aus der Cilindrophan‐Biosynthese stammt, katalysiert den Aufbau einer Aryl‐Aryl‐Verbindung in der nativen Reaktion, die zur Synthese eines komplexen Naturstoffs beiträgt.^[183]^ Darauf aufbauend wurde der biokatalytische Nutzen von CylK für die C‐Alkylierung von Resorcinolen gezeigt, wobei die Kupplung verschiedener Alkylbausteine in C2‐Position gelang.

Wie diese Beispiele zeigen, sind weitere Untersuchungen notwendig, um diese neuartigen, jedoch vielversprechenden Transformationen weiter auszubauen und auf weitere Substrate neben Verbindung **161** anzuwenden, um einen weitergehenden Nutzen für die späte Funktionalisierung von Arenen zu eröffnen (Schema 28 b).^[184]^

### 4.3 Pictet‐Spengler‐Reaktion

Zum Aufbau heterozyklischer Molekülgerüste im Wirkstoffdesign ist die Pictet‐Spengler‐Reaktion ein besonders nützliches Mittel, um verschiedenartig dekorierte bizyklische Motive für die Untersuchung von Struktur‐Aktivitäts‐Beziehungen zu erhalten.^[185]^ An die Kondensation zwischen einem elektronenreichen Arylethylamin und einem Aldehyd oder Keton schließt sich im Sauren ein Ringschluss des intermediären Iminium‐Ions an. Alkaloide, Tetrahydroisochinoline (THQ) und β‐Carboline können stereoselektiv hergestellt werden und bieten damit wichtige Naturstoffe oder pharmakologische Gerüste.[^186^, ^187^]

Norcoclaurin‐Synthase (NCS) und Strictosidin‐Synthase (STR) sind für biotechnologische Anwendungen gut untersuchte Pictet‐Spenglerasen (PSasen; Schema 29 a).^[187]^ (*S*)‐Norcoclaurin (**167**) wird durch NCS‐katalysierte Kondensation von Dopamin (**166**) mit 4‐Hydroxyphenylacetaldehyd erhalten.^[188]^ Die PSase STR katalysiert hingegen die Zyklisierung von Tryptamin (**168**) mit Secologanin (**169**), welches das 1,2,3,4‐Tetrahydro‐β‐carbolin‐Gerüst (THBC) aufbaut, das Teil des Indolalkaloids (*S*)‐Strictosidin (**170**) ist (Schema 29 b).^[189]^

**Scheme 29:**
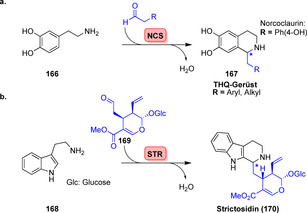
Enzymkatalysierte Pictet‐Spengler‐Reaktion. a) Kondensation von Dopamin mit Aldehyden ergibt verschiedene THQs (**167**). b) Stricosidin (**170**) wird durch Kondensation zwischen Tryptamin (**168**) und Secologanin (**169**) synthetisiert.

Zur Einführung von Diversität kann der Substituent am C1‐Atom des Heterozyklus durch Wahl der gewünschten Aldehyd‐Komponente variiert werden.^[190]^ Weiterführende Untersuchungen des Substratspektrums verdeutlichten, dass verschiedene Substituenten, vor allem Phenyl‐ und Alkyl‐, in hohen Enantioselektivitäten eingeführt werden können.^[191]^ Die Gruppen um Hailes und Ward verfolgten kürzlich das Ziel, PSasen für sperrigere Keton‐Substrate (**171**) zugänglich zu machen.^[192]^ Dafür wurde eine trunkierte NCS (Δ29*Tf*NCS) mit geringer promiskuitiver Aktivität gegenüber 4‐Hydroxyphenylaceton rational optimiert, sodass 1,1′‐bis‐substituierte THQs (**173**) ausgehend von Methylketonen erhalten wurden (Schema 30).

**Scheme 30:**
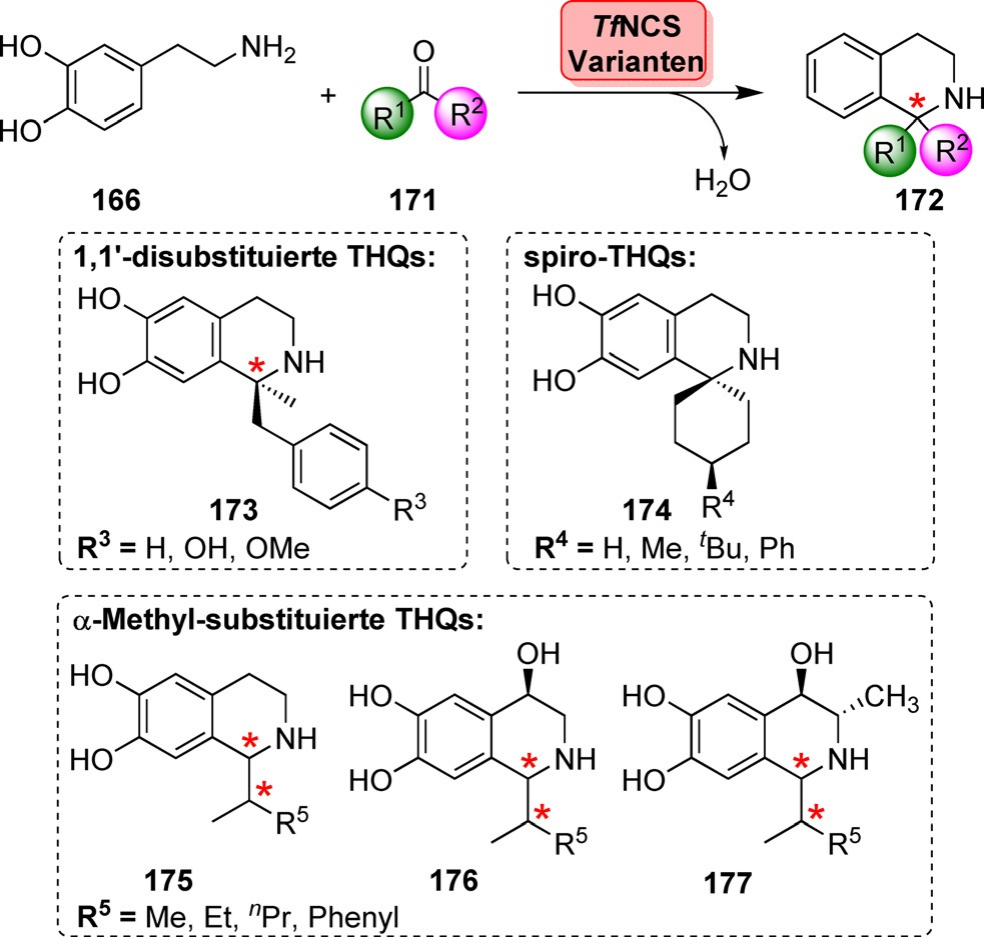
Engineering der T*f*NCS erweiterte das Substratspektrum in Richtung disubstituierte, Spiro‐ und α‐Methyl‐THQs. Einige Produkte sind repräsentativ gezeigt.

Besonders interessant ist ebenfalls, dass Spiro‐Derivate (**174**) erhalten wurden, indem Dopamin mit Cyclohexanonen gekuppelt wurde. Insbesondere diese Transformation ist eine attraktive Methode zur Diversifizierung. Außerdem konnte das Substratspektrum gegenüber α‐substituierten Aldehyden erweitert werden, was die THQs **175**–**177** mit exzellenten Diastereoselektivitäten in Bezug auf die zwei aufgebauten Stereozentren lieferte.^[193]^

Frühe Studien zum Substratspektrum und Engineering der aktiven Taschen von STRs deuteten darauf hin, dass diese Enzyme promiskuitiv gegenüber substituierten Tryptaminen und verschiedenen aliphatischen und aromatischen Aldehyden sind, jedoch mit geringerer Effizienz im Vergleich zum nativen Aldehyd (**169**).[^194^, ^195^, ^196^] Durch Expressionsoptimierung und das Screening verschiedener STRs konnte das Spektrum in Richtung kurzkettiger Aldehyde erweitert werden. Für das Enzym *Rs*STR wurde dabei eine unerwartete (*R*)‐Konfiguration des resultierenden THBC (**179**) festgestellt, während für sperrigere Aldehyde die (*S*)‐Konfiguration überwiegt (Schema 31). Strukturelle Untersuchungen und Modelling‐Studien bieten eine Erklärung für diesen auffälligen Selektivitätswechsel: Kleinere Aldehyde werden in inverser Orientierung gebunden, und somit wird die unterschiedliche Stereopräferenz durch den Aldehyd bestimmt.^[197]^ Diese Selektivitätseigenschaft konnte dann ausgenutzt werden, um einen Vorläufer (**182**) des (*R*)‐Harmicins aus **178** und **180** zu synthetisieren.^[198]^

**Scheme 31:**
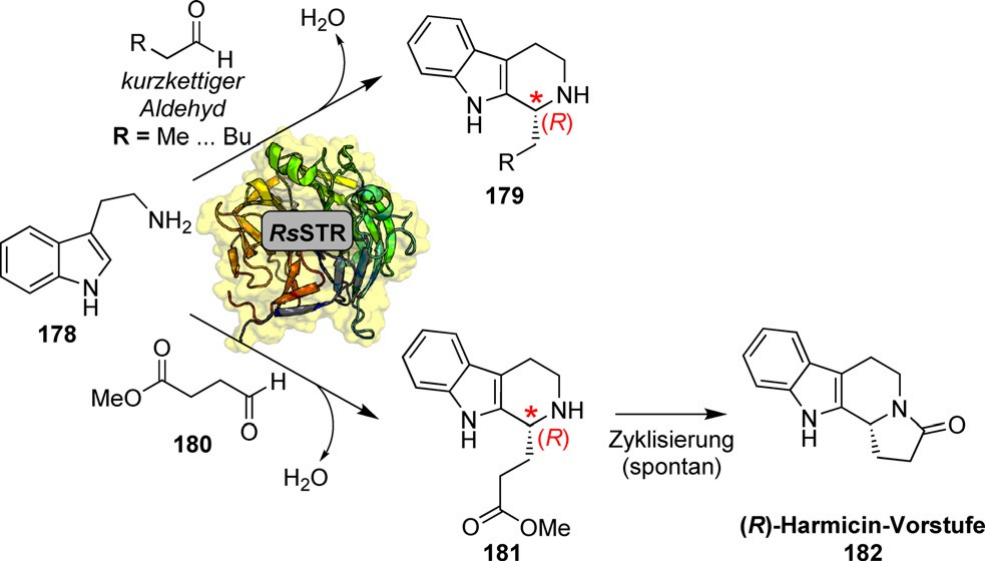
Kondensation von Tryptamin mit kurzkettigen Aldehyden resultiert in einer unerwarteten (*R*)‐Konfiguration des Reaktionsprodukts, während die (*S*)‐Konfiguration für sterisch anspruchsvollere Aldehyd‐Substrate überwiegt.

## 5 Selektiver enzymkatalysierter Aufbau von Amidbindungen

### 5.1 Amidsynthese in der Natur

Amidbindungen sind die häufigsten Bindungsmotive in Pharmazeutika, wie eine kürzlich erschienene Analyse über die Häufigkeit funktioneller Gruppen in Publikationen im Bereich der medizinischen Chemie verdeutlichte.^[199]^ Es ist daher nicht überraschend, dass gemäß einer Untersuchung aus dem Jahr 2011 16 % aller Reaktionen, die in der medizinischen Chemie angewendet werden, Amidkupplungen sind.^[200]^ Ein breites Portfolio an Methoden ist heutzutage verfügbar, und die Entwicklung selektiver und nicht toxischer Amidierungsmethoden geht kontinuierlich weiter. Typische Ansätze erfordern die Aktivierung von Carbonsäuren, z. B. durch Bildung von Säurechloriden, die Nutzung von Carbodiimiden oder Uronium‐Reagenzien sowie eine geeignete Schutzgruppenstrategie und häufig toxische, umweltbelastende Lösungsmittel.^[201]^ Diese Defizite bieten einen Anreiz, um neue Reaktionen zu entwickeln, die Amide selektiv und unter milden Bedingungen herstellen.

Neben der Peptidbindung als zentrales Bindungsmotiv in Proteinen existiert eine große Anzahl von Enzymen, die diese äußerst wichtige Verbindung in Naturstoffen aufbauen und auch für die synthetisch orientierte Enzymkatalyse an Bedeutung gewinnen. Im Allgemeinen können Hydrolasen, einige Transferasen und ATP‐abhängige Ligasen Amidierungsreaktionen katalysieren. Hydrolase‐basierte Ansätze laufen in den meisten Fällen über eine Aminolyse, der oftmals eine Veresterung vorangeht (Schema 32). Ein Ausnahme davon ist die kürzlich veröffentlichte Lipase SpL, die aus freien Carbonsäuren und Aminen unmittelbar Amide in Mischungen aus organischem Solvens und Wasser bildet (Schema S2, Hintergrundinformationen).^[202]^ Ein Überblick über wichtige Beispiele der Hydrolase‐katalysierten Amidierungen mithilfe von Lipasen und Penicillin‐G‐Acylasen findet sich in Supporting Section 6 (siehe Hintergrundinformationen). Transferasen übertragen aktivierte Acyldonoren, während in ATP‐abhängigen, enzymkatalysierten Amidierungen Carbonsäuren direkt in Wasser unter milden Bedingungen in Anwesenheit von Aminen zu Amiden umgesetzt werden. Dieser Reaktionstyp wird in diesem Aufsatz vor dem Hintergrund der späten Modifizierungen näher beleuchtet.^[203]^

**Scheme 32:**
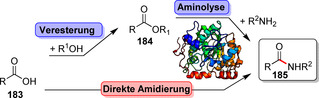
Strategien für den enzymkatalysierten Aufbau von Amidbindungen ausgehend von einer Carbonsäure (**183**). Die Hydrolase‐katalysierte Aminolyse erfordert eine vorherige Veresterung, während in einem direkten Ansatz die Carbonsäure als Substrat fungiert und direkt zum Amid (**185**) umgesetzt wird.

In den vergangenen Jahren wurden zahlreiche Enzyme, die Teil der ANL (Acyl‐CoA‐Synthetase, Nicht‐ribosomale Peptid‐Synthetase, Luciferase)‐Superfamilie der adenylierenden Enzyme sind, zugänglich gemacht. Diese ATP‐abhängigen Enzyme spielen eine zentrale Rolle, bilden jedoch eine hochdiverse Enzymklasse: Alle Vertreter nutzen ATP, um die Carbonsäure (**183**) für den Acyltransfer zu aktivieren. In der nicht‐ribosomalen Amidsynthese verläuft die Amidierung über drei Schritte: 1) Adenylierung; 2) Thiolyse; und 3) Kondensation (Schema 33).^[204]^ ATP‐*grasp*‐Enzyme bilden ein Acylphosphat‐Intermediat (**186**) durch Angriff auf die γ‐Phosphatgruppe des ATP, während Adenylierungsdomänen (A‐Domänen) und Amidsynthetasen die Entstehung eines intermediären Acyladenylats (**187**) katalysieren.^[205]^

**Scheme 33:**
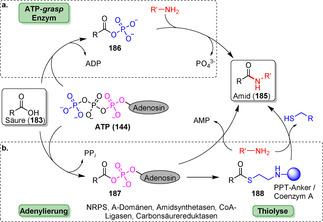
Aktivierung der Carboxygruppe durch ATP‐abhängige Enzyme. Es überwiegen zwei verschiedene Strategien zur Nutzung von ATP: a) In ATP‐*grasp*‐Enzymen wird das γ‐Phosphat durch nucleophilen Angriff des Carboxylats übertragen, wodurch ein gemischtes Anhydrid (**186**) entsteht. b) Die Adenylierungsdomäne resultiert in einem Acyladenylat (**187**). In Abhängigkeit vom Enzym wird die aktivierte Spezies entweder direkt von einem Amin angegriffen oder verläuft über einen intermediären Thioester (**188**). NRPS: nicht‐ribosomale Peptidsynthetase.

### 5.2 Direkter Aufbau von Amidbindungen: Beispiele und aktuelle Entwicklungen in der späten Funktionalisierung

l‐Aminosäure‐Ligasen sind die häufigsten Mitglieder der ATP‐*grasp*‐Enzymfamilie. Oftmals ermöglichen sie den Aufbau von Peptidbindungen in der Biosynthese. Ein recht promiskuitives ATP‐*grasp*‐Enzym wurde in der Biosynthese von Tabtoxin S gefunden. TabS ist in der Lage, verschiedene Dipeptide aus nicht‐geschützten Aminosäuren zu synthetisieren. Insgesamt führten 136 verschiedene Kombinationen zu den entsprechenden Dipeptiden, wobei auch nicht‐natürliche Aminosäuren genutzt wurden.^[206]^ Obwohl einige weitere l‐Aminosäure‐Ligasen charakterisiert wurden, ist ihre Anwendung aufgrund geringer Produktivität im Vergleich zur konventionellen Peptidsynthese und hoher Substratspezifität eher wenig verbreitet.[^207^, ^208^, ^209^, ^210^] Ein wichtiger Fund für die späte Funktionalisierung war die Identifikation des *grasp*‐Enzyms PGM1 im Biosyntheseweg des Antibiotikums Pheganomycin (**190**). PGM1 katalysiert selektiv die N‐terminale Kupplung zwischen dem Biosynthese‐Vorläuferpeptid (**189**) mit verschieden substituierten Essigsäure‐Derivaten und liefert somit eine wertvolle Reaktion für die Peptidmodifikation (Schema 34).^[211]^

**Scheme 34:**
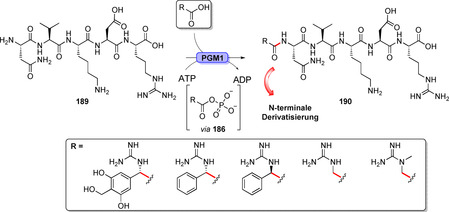
N‐terminale Derivatisierung des Pheganomycin‐Derivats mithilfe der Peptidligase PGM1. Das Enzym katalysiert die selektive Acylierung am N‐Terminus des Peptids.

*N*‐Acyltransferasen ermöglichen die Übertragung von aktivierten Acylgruppen auf verschiedene Amine. Zum Beispiel wurde die Acylierung von Aminen mit Vinylestern in Wasser für die Transferase MsAcT von Paradisi und Coautoren gezeigt.^[212]^ In einer kürzlich erschienenen Studie wurde die Reaktivität von MsAcT deutlich gesteigert, indem der Serin‐Rest der katalytischen Triade in einen Cysteinrest mutiert wurde.^[213]^ Die Variante S11C ermöglichte auch die Synthese von Thioestern und schwierigen tertiären Amiden mit einer industriell bedeutsamen Substratbeladung (Schema 35 a). Darüber hinaus berichteten Lovelock und Coautoren über eine Plattform aus zwei Enzymen, die CoA‐Ligasen mit Acyltransferasen vereinigt (Schema 35 b).^[214]^ Dabei wird ein Coenzym‐A‐Thioester ausgehend von der Säure **183** durch eine Ligase gebildet und in Anwesenheit der *N*‐Acyltransferase im zweiten Schritt von einem Amin‐Nucleophil angegriffen. Unter der Voraussetzung, dass geeignete Paarungen aus Ligase und Acylase gefunden werden, kann eine Vielfalt von Amidierungen mit dieser Methode abgedeckt werden, ohne dass zuvor aktivierte Acyldonoren verwendet werden müssen.

**Scheme 35:**
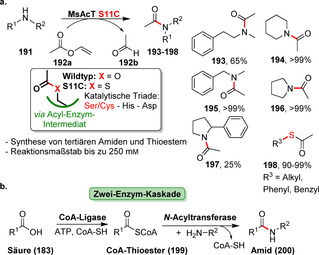
a) Die *N*‐Acyltransferase MsAcT S11C ermöglicht schwierige Acetylierungen in hoher Substratkonzentration als Folge des Austauschs des Serins in der aktiven Tasche gegen einen Cysteinrest, sodass ein kovalentes Thioester‐Intermediat gebildet wird. b) Die Kombination einer CoA‐Ligase mit einer *N*‐Acyltransferase ermöglicht es, ein breites Spektrum an Amiden zu synthetisieren.

Adenylierungsdomänen (A‐Domänen) sind weit verbreitet in NRPS‐Systemen und bieten einen Zugang zur ATP‐abhängigen Aktivierung von Carboxygruppen. Es wurde gezeigt, dass eigenständige A‐Domänen aus einem NRPS‐System, verantwortlich für die Biosynthese von Streptothricin‐Antibiotika in *Streptomyces* sp., als autarke Biokatalysatoren fungieren, welche die Adenylierung der Aminosäure l‐β‐Lysin katalysieren. Diese wiederum wird entweder von einer PCP‐Domäne (Peptid‐Carrier‐Protein) oder einem Carrier‐gebundenen l‐β‐Lysin angegriffen, wodurch ein Oligopeptid entsteht.^[215]^ Darüber hinaus wurde die Amidierung von Fettsäuren durch A‐Domänen für eine Reihe von Aminen katalysiert, was auch die Synthese seltener *N*‐Acylhistidine neben anderen acylierten Verbindungen ermöglichte.^[216]^ Auch die Diversifizierung von Tryptophan verlief erfolgreich durch Verwendung einer A‐Domäne aus der Tyrocidin‐Synthetase (TycA).^[217]^ Jedoch begrenzt ein stark spezialisiertes Substratspektrum oftmals die breite Anwendung von A‐Domänen aus NRPS‐Systemen.

Eine Alternative sind Carbonsäurereduktasen (CAR), die nativ die Reduktion von Carbonsäuren (**183**) in die entsprechenden Aldehyde (**201**) katalysieren.[^218^, ^219^] Strukturuntersuchungen und Engineering machten deutlich, dass CARs als Multidomänen‐Enzyme fungieren, die aus einer abgegrenzten A‐, PCP‐ und Reduktionsdomäne bestehen (Schema 36 a).^[220]^ Durch Wood et al. wurde gezeigt, dass CARs zu Amidierungen fähig sind, wenn ein Überschuss an Amin anstatt des Cosubstrats NADPH eingesetzt wird, sodass die Reduktionsfunktion unterdrückt ist.^[221]^ Auf diesem Weg wurde eine Palette von Benzoe‐ und Zimtsäureamiden synthetisiert. In einer Folgestudie wurde dieses Konzept weiter verschlankt, indem eine verkürzte CAR‐Variante, die lediglich aus der A‐Domäne (CAR*mm*‐A) besteht, eingesetzt wurde. CAR*mm*‐A erlaubte die selektive Monoacylierung von Diaminen, ohne dass Schutzgruppen erforderlich waren. Darauf basierend wurde ein Set aus Carbonsäuren in einem Maßstab von 10 mm für die Ein‐Schritt‐Amidierung eingesetzt, was zahlreiche Amide (**208**–**216**) lieferte, z. B. den Vasodilator Cinepazid (**216**) (Schema 36 b).^[222]^

**Scheme 36:**
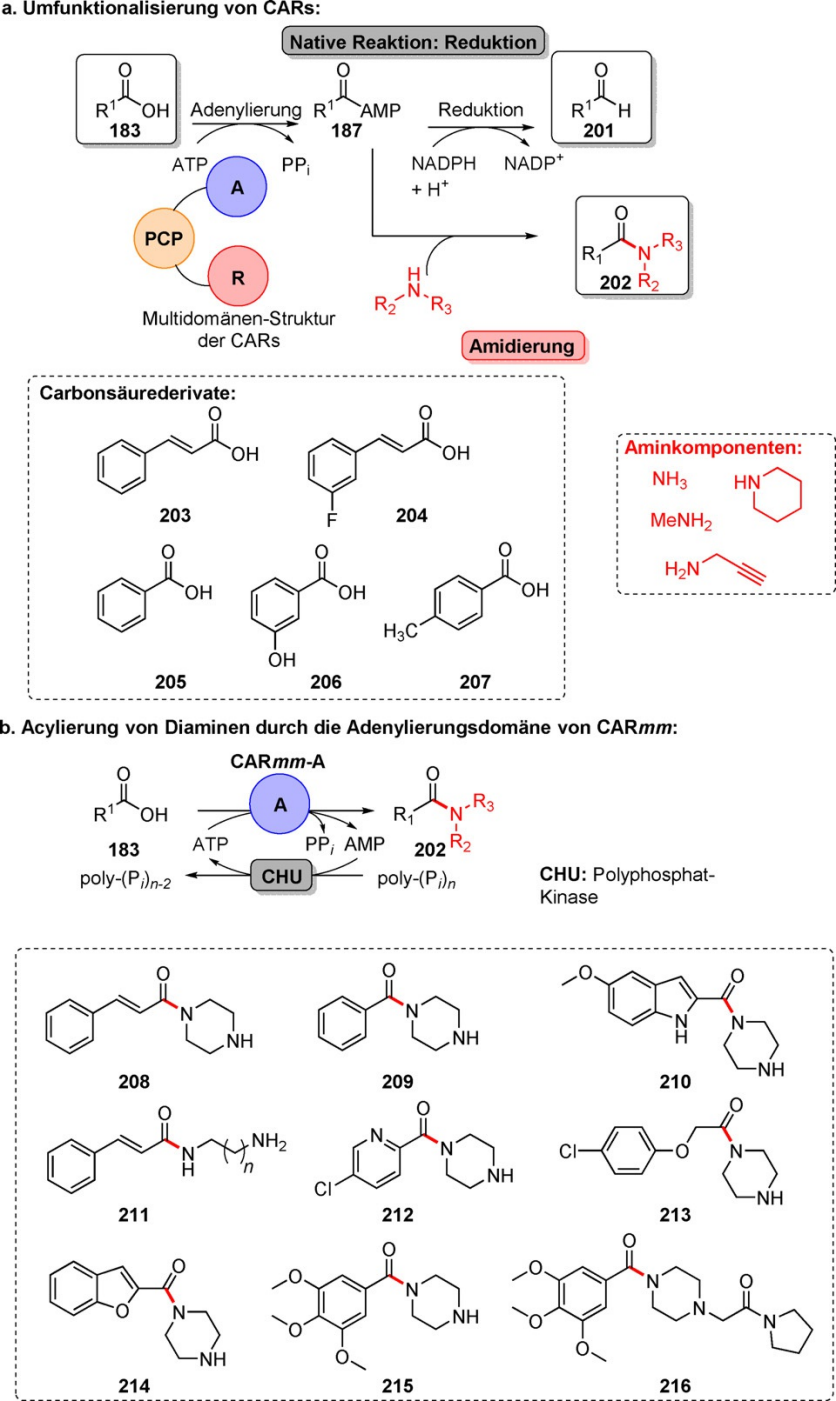
a) Carbonsäurereduktasen können für den Aufbau von Amidbindungen statt zur Reduktion einer Carbonsäure genutzt werden. b) Lediglich die A‐Domäne reicht für die Amidierung aus, wie für die Nutzung einer trunkierten CAR (CAR*mm*‐A) gemeinsam mit einer Kinase für die begleitende ATP‐Regeneration gezeigt wurde.

Obwohl das Substratspektrum und die schwierige Handhabung aufgrund ihrer schwachen Expression und Löslichkeit die Nutzung einschränken, erhalten Amidsynthetasen zunehmende Bedeutung für die späte Modifizierung. Wessjohann und Coautoren berichteten über die homologen Amidsynthetasen CloL, SimL und CouL, die zur Modifikation von Aminocumarinen (**217**) in einem modularen Verfahren geeignet waren. Eine kleine Bibliothek aus Derivaten wurde mithilfe dieses Systems im Milligramm‐Maßstab synthetisiert (Schema 37 a).^[223]^ Für die Amidsynthetase XimA wurde gezeigt, dass sie den letzten Schritt der Biosynthese von Xiamenmycin A durch Kupplung von l‐Threonin mit der freien Vorläufer‐Carbonsäure (Xiamenmycin B) katalysiert (Schema 37 b). Die Autoren erweiterten das Aminosäurespektrum durch rationales Design von Mutanten ausgehend von l‐Threonin in Richtung elf verschiedener l‐ und d‐Aminosäuren.^[224]^

**Scheme 37:**
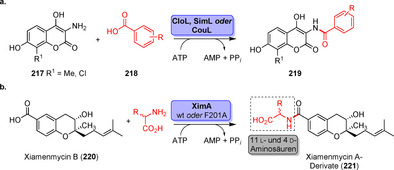
a) Amidkupplung von Aminocumarinen (**217**), um acylierte Aminocumarine (**219**) zu erhalten. b) Derivatisierung von Xiamenmycin durch gezielte Kupplung der Aminosäuren mit **220** durch Anwendung des XimA‐Wildtyps oder einer Mutante.

Kürzlich wurde über die synthetische Anwendbarkeit der Synthetase McbA aus *Marinactinospora thermotolerans* von Petchey et al. berichtet.^[225]^ In der natürlichen Reaktion katalysiert McbA die Amidierung von β‐Carbolinen (**222**), dennoch ist McbA nicht ausschließlich auf das natürliche Substrat beschränkt (Schema 38). Breit ausgelegte Untersuchungen zum Substratspektrum ergaben, dass ein weiter Bereich aus aromatischen Carbonsäuren von McbA akzeptiert wird.^[226]^ Ein fast äquimolares Verhältnis von Säure zu Amin stellt zudem einen wichtigen Fortschritt für den Aufbau von Amidbindungen dar, insbesondere im Hinblick auf den präparativen Maßstab. In weiteren Untersuchungen wurde auch das Aminspektrum in Richtung verschiedener aliphatischer und zyklischer Gruppen verbreitert. Beachtenswert war, dass die Acylierung schwach nucleophiler Amine gelang, wie für eine Reihe von Anilinen gezeigt (**233**–**237**).^[227]^

**Scheme 38:**
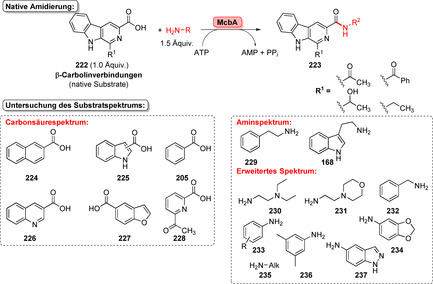
Die Amidsynthetase McbA katalysiert die Amidierung von β‐Carbolinen in der natürlichen Reaktion. McbA akzeptiert verschiedene aromatische Carbonsäuren. Das Aminspektrum, das für die Säure **222** mit R^1^=Acetyl untersucht wurde, zeigt, dass diverse aliphatische und aromatische Aminbausteine von McbA akzeptiert werden.

Der Aufbau von Amidbindungen mittels Biokatalyse steckt noch in den Kinderschuhen, und Untersuchungen zur späten Diversifizierung haben gerade erst begonnen, wie die hier gezeigten Beispiele abbilden. Derzeit sind die Skalierbarkeit, das Spektrum der akzeptierten Carbonsäuren bzw. Amine sowie die zur Verfügung stehenden Enzyme starke Einschränkungen, die in zukünftigen Studien angegangen werden müssen, um die Methode weiterzuentwickeln.

## 6 Reduktion von Doppelbindungen

### 6.1 C=O‐Reduktion

Die Reduktion von C=O‐Doppelbindungen in den entsprechenden Alkohol ist eine atomökonomische Alternative zur stereoselektiven Oxyfunktionalisierung. Eine große Bandbreite verschiedener Enzyme ist zur C=O‐Reduktion fähig, wie von Hollmann et al. in einem Übersichtsartikel dargestellt, u. a. Ketoreduktasen (KRED), Aldo‐Ketoreduktasen (AKR), mittelkettige Dehydrogenase‐Reduktasen (MDR) sowie kurzkettige Dehydrogenasen.^[228]^ Neben der herausragenden Bedeutung für die industrielle Biokatalyse haben C=O‐Reduktionen auch ihre Bedeutung in der späten Funktionalisierung.

Gong et al. beschäftigten sich mit einem Ansatz zur Synthese von Atorvastatin, einem wichtigen Wirkstoff zur Cholesterinsenkung.^[229]^ Die Synthese der Seitenkette (ATS‐7, **239**) ist durch ihre hohe Stereoselektivität ein exzellentes Beispiel für eine KRED‐katalysierte Biotransformation (Schema 39). Es wurden Anstrengungen unternommen, um eine KRED‐Variante mit gesteigerter Aktivität und Thermostabilität durch gerichtete Evolution zu generieren, sodass schließlich der chirale Alkohol in 87 % Ausbeute und >99.5 % Diastereomerenüberschuss (*de*) in einem Maßstab von 100 mL bei 40 °C zugänglich war.^[229]^ Die biokatalytische Einführung von Deuterium ist ein bemerkenswertes Beispiel für die späte Markierung durch C=O‐Reduktion (Schema 40). Die Anwesenheit von Deuterium‐Atomen in Pharmazeutika kann in verbesserten pharmakokinetischen Eigenschaften, wie z. B. höherer metabolischer Stabilität, resultieren.^[230]^ Rowbotham et al. präsentierten in diesem Zusammenhang einen eleganten Ansatz für die asymmetrische Deuterierung durch NADH‐Reduktasen, die H_2_ als Reduktionsmittel zusammen mit ^2^H_2_O als Isotopenquelle nutzen. Hydrogenase und NAD^+^‐Reduktase wurden auf Kohlenstoffpartikeln coimmobilisiert, wodurch das H_2_‐getriebene System in der Lage ist, NAD^+^ zu 4‐[^2^H]‐NADH zu reduzieren. In Verbindung mit einer KRED wird der Deuteriumtransfer auf ein Keton (**240**) möglich, wodurch ein markierter Alkohol (**241**) hergestellt wurde.^[231]^

**Scheme 39:**
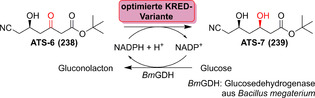
Asymmetrische Reduktion von ATS‐6 zur Synthese der Atorvastatin‐Seitenkette durch eine optimierte KRED‐Variante.

**Scheme 40:**
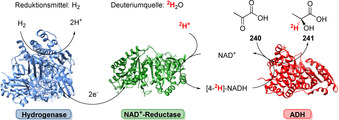
Reduktive Deuterierung von Carbonylgruppen durch Verwendung eines heterogenen biokatalytischen Cofaktorregenerierungssystems zusammen mit einer KRED und ^2^H_2_O, wodurch Deuterium bereitgestellt wird.

### 6.2 Späte reduktive Aminierung und Aminoxidation

Die Biokatalyse ist ein starkes Werkzeug für die C=N‐Reduktion geworden, um Amine selektiv herzustellen. Viele Enzymklassen können diese Reaktion bewerkstelligen, wie z. B. Iminreduktasen (IREDs), reduktive Aminasen (RedAms), Transaminasen, Amindehydrogenasen (AmDH) und kurzkettige Dehydrogenasen/Reduktasen (short chain dehydrogenases/reductases, SDRs). Hier beschäftigen wir uns mit aktuellen Fortschritten in der Synthese von Aminen vor dem Hintergrund der späten Modifizierung.

Über die biokatalytische reduktive Aminierung für die Synthese pharmazeutischer Verbindungen und chemischer Bausteine wurde ausgiebig zuvor berichtet.[^232^, ^233^] (*R*)‐Rasagilin (**244**), ein Wirkstoff, der zur Behandlung der Parkinsonschen Krankheit eingesetzt wird, ist eine geeignete Zielstruktur für RedAms. RedAms besitzen die Fähigkeit, die asymmetrische Reduktion zwischen Ketonen oder Aldehyden zu katalysieren, indem sowohl die Iminbildung als auch die nachfolgende Reduktion kombiniert werden (Schema S4, siehe Hintergrundinformationen).^[232]^ Im Gegensatz dazu muss im Falle von IREDs das Imin a priori entstehen und wird nachfolgend enzymkatalytisch reduziert.

Die Synthese der Verbindung **244**, entweder ausgehend von einem prochiralen Ketonvorläufer (**242**) oder einem racemischen Amin (*rac*‐**244**), eröffnete die Nutzung verschiedener biokatalytischer Ansätze. Aleku et al. berichteten über eine Variante des Enzyms *Asp*RedAm, das **244** direkt aus **242** und Propargylamin (**243**) mit >97 % Umsatz und exzellentem *ee* herstellen konnte (Schema 41 a).

**Scheme 41:**
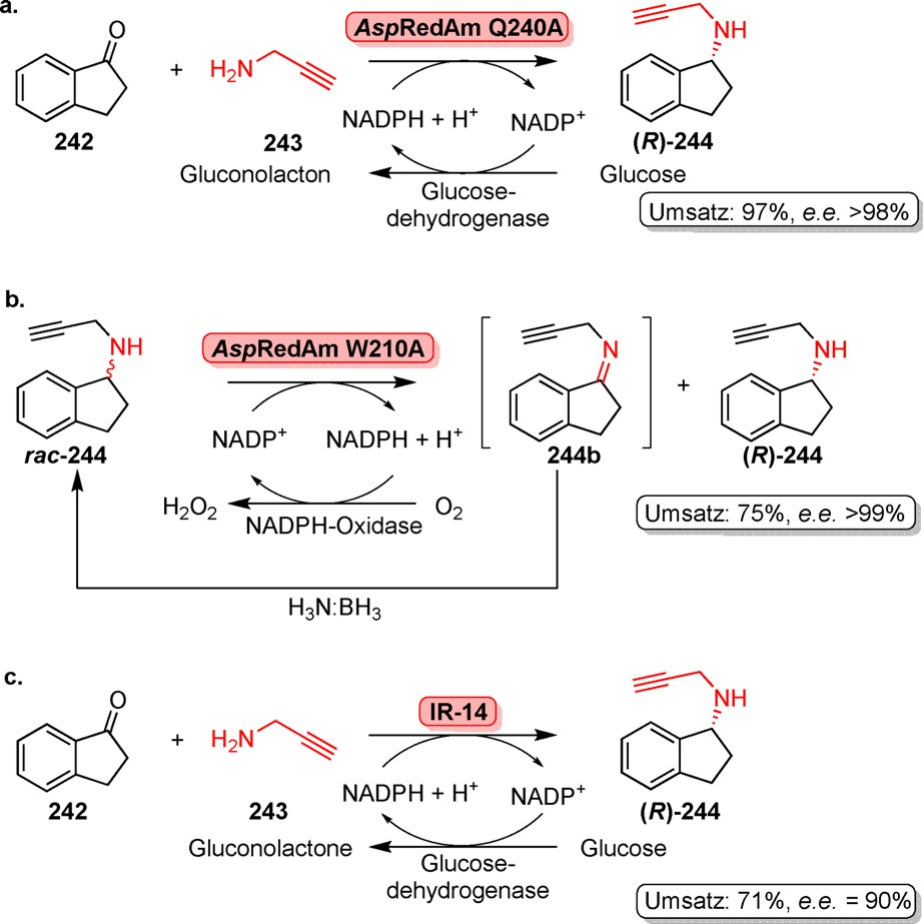
Biokatalytische Ansätze für die Synthese von (*R*)‐Rasagilin (**244**).

Eine andere *Asp*RedAm‐Variante, die für die Oxidation von (*S*)‐**244** selektiv ist, wurde genutzt, um exklusiv das Imin **244b** zu erhalten, wodurch das gewünschte Enantiomer (*R*)‐**244** durch dynamisch‐kinetische Racematspaltung mit >99 % *ee* akkumulierte (Schema 41 b).^[234]^ Ebenso identifizierten Matzel et al. ein Enzym, das **244** in einem Schritt aus dem gleichen Ausgangsstoff produzieren kann (Schema 41 c).^[233]^

Die Synthese des Lysin‐spezifischen Demethylase‐1‐Inhibitors (GSK2879552, **248**) für die Behandlung des kleinzelligen Lungenkrebses und akuter Leukämie ist ein aktuelles Beispiel für die späte Aminierung:^[235]^ Der *tert*‐Butylester (1*R*,2*S*)‐**247** wurde aus dem Vorläufer‐Aldehyd (**245**) erhalten (Schema 42). Durch ausgiebiges Engineering und die vorherige Identifizierung einer geeigneten IRED, die aus einer zuvor etablierten Bibliothek stammt (IRED‐46),^[236]^ konnte die resultierende Variante gleichzeitig die kinetische Racematspaltung und reduktive Aminierung katalysieren. Beachtenswert ist zudem, dass der optimierte Biokatalysator die Anforderungen für einen industriell nutzbaren Prozess erfüllte.

**Scheme 42:**
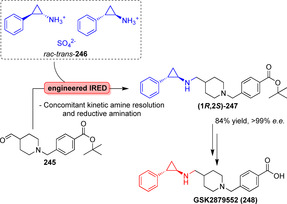
Biokatalytische kinetische Racematspaltung und reduktive Aminierung für die Herstellung des LSD‐1‐Inhibitors GSK2879552 (**248**) durch eine hochgradig optimierte IRED.

Aminoxidasen können die Gegenreaktion zu IREDs katalysieren und eröffnen damit ein attraktives Mittel für verschiedene Arten der Funktionalisierung von N‐Heterozyklen. Aus der Monoaminoxidase‐N (MAO‐N) von *Aspergillus niger* resultierte nach mehreren Generationen von gerichteter Evolution und Protein‐Engineering ein Set verschiedener Varianten (z. B. D5, D9, D10) mit komplementärer Substratpräferenz und hoher Aktivität.^[237]^ Diese Varianten sind besonders aktiv gegenüber zyklischen fünfgliedrigen Ring‐Aminen und katalysieren die Oxidation zum korrespondierenden Amin oder Iminium unter milden Bedingungen (Raumtemperatur, pH 7.5, atmosphärischer Sauerstoff). Diese Transformation ist äquivalent zu der α‐C‐H‐Aktivierung zu einem Amin, und die resultierenden Imine bzw. Iminium‐Ionen wurden für eine Reihe von Transformation eingesetzt (Schema 43): 1) die Addition von Nucleophilen (z. B. CN, Bisulfit), 2) Oxidation zu Lactamen (**252**)^[238]^ und 3) Bausteine für Mehrkomponenten‐Reaktionen, z. B. Ugi und Ugi‐Smiles.^[239]^ MAO‐N‐Varianten wurden außerdem verwendet, um Pyrrole sowie Pyridine aus den entsprechenden Dihydro‐ und Tetrahydro‐Vorstufen zu generieren.[^240^, ^241^] Ausgiebiges Engineering der MAO‐N erwies sich als nützlich in der Desymmetrisierung von Wirkstoff‐Bausteinen, um enantiomerenreine Amin‐Vorstufen zu bilden.^[242]^

**Scheme 43:**
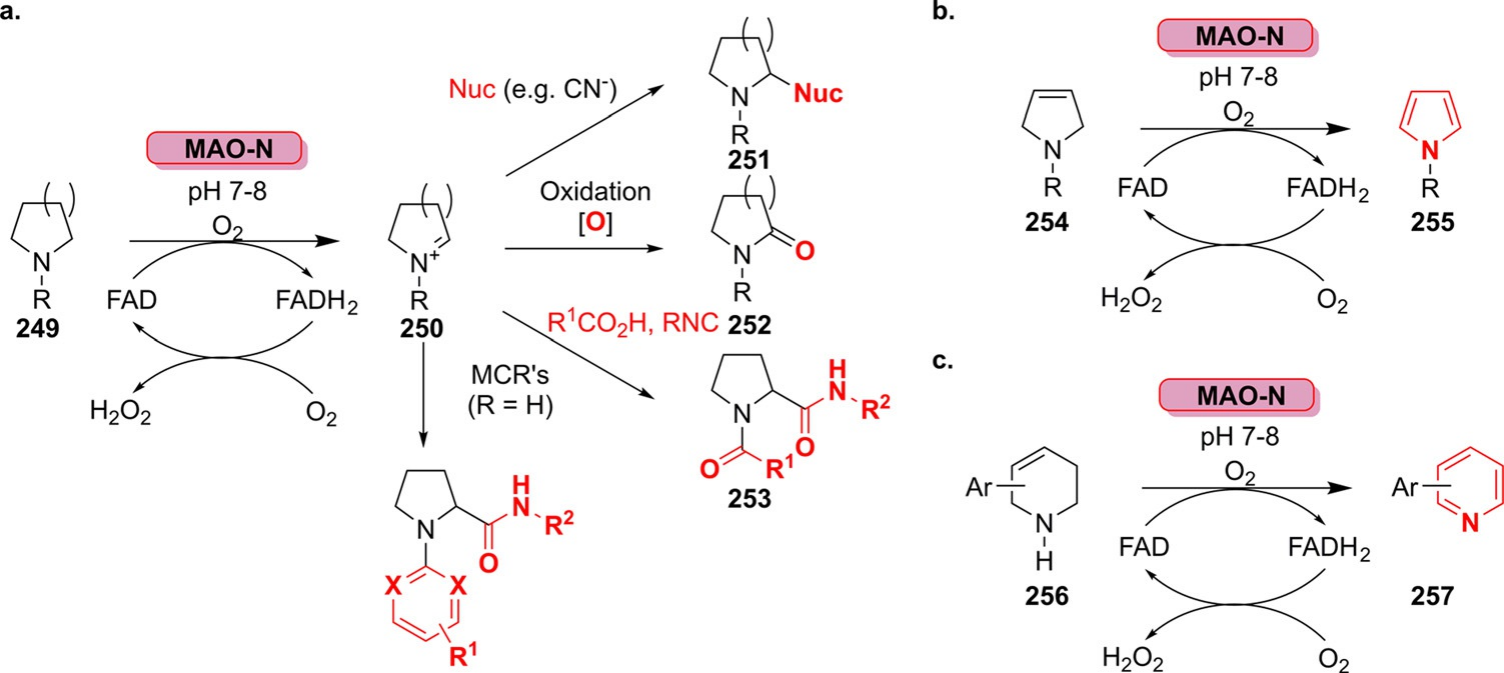
Vielfältige Funktionalisierungsstrategien mithilfe von MAO‐N und ihre Anwendung in der Synthese von Heterozyklen. a) Funktionalisierung des α‐Atoms zum N‐Atom; b) Pyrrol‐Synthese; c) Pyridin‐Synthese.

Der Antidiabetes‐Wirkstoff Sitagliptin (**259**) spiegelt ein ausgezeichnetes Beispiel für die späte Aminierung durch eine von Codexis und Merck entwickelte Transaminase wider (Schema 44).^[243]^ Die schrittweise Evolution der Transaminase ermöglichte die reduktive Aminierung der entsprechenden Keton‐Vorstufe (**258**). Ausgehend von einem verkürzten Methylketon‐Substrat wurde das aktive Zentrum in Richtung des sterisch anspruchsvolleren Ketons evolviert, sodass schließlich **259** mit exzellenter Enantioselektivität im industriellen Maßstab gewonnen werden konnte.

**Scheme 44:**
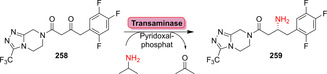
Eine optimierte Transaminase ermöglicht die späte Aminierung der Keton‐Vorstufe **258** zu Sitagliptin (**259**).

## 7 Aufstrebende Gebiete für die späte Modifizierung

### 7.1 Photobiokatalyse

Die Photobiokatalyse ist ein neues Konzept, das die Merkmale der organischen Photo‐ mit der Biokatalyse verbindet und im vergangenen Jahrzehnt zunehmend an Interesse gewonnen hat. Im Hinblick auf die C‐H‐Aktivierung ermöglichen lichtgetriebene Reaktionen eine hohe katalytische Promiskuität sowie saubere Reaktionsbedingungen und hohe Kompatibilität zu funktionellen Gruppen.^[244]^ Eine Reihe von Photokatalysatoren, z. B. Übergangsmetalle oder organische Gerüstverbindungen, sind gut etabliert für die Nutzung von Licht als Energiequelle, während die Entwicklung der biokatalytischen Gegenstücke noch in den Kinderschuhen steckt.^[245]^ Eine ausführliche Diskussion über den Hintergrund der photochemischen Anregung von Enzym‐Cofaktoren kann in einem kürzlich erschienenen Übersichtsartikel von Sandoval et al. gefunden werden.^[246]^

Photokatalytische Reaktionen können mit enzymkatalysierten Transformationen kombiniert werden, um neue katalytische Funktionen auszuschöpfen. Insbesondere NAD(P)H‐abhängige Enzyme sind attraktive biokatalytische Einheiten, da der Cofaktor im visuellen Bereich des Spektrums angeregt werden kann. Die Anregung der 1,4‐Dihydropyridin‐Gruppe (**160**) zu dem stärkeren Reduktionsmittel **261** führt zu einer Zunahme des Reduktionspotenzials von NAD(P)H, was die Reduktion verschiedener funktioneller Gruppen erlaubt.^[247]^

Hysters Gruppe war in der Lage, einige Enzyme umzufunktionieren, indem die photochemischen Eigenschaften der entsprechenden beteiligten Cofaktoren ausgeschöpft wurden. Die Anregung von **260** in einer KRED wurde für die Dehalogenierung achiraler substituierter Lactone (**262**) genutzt. Infolge der Anregung von NAD(P)H verlief die Transformation des racemischen Lactons (**262**) zum dehalogenierten, enantiomerenreinen Derivat (**264**) über die Bildung einer intermediären Radikalspezies (**263**; Schema 45 b).[^247^, ^248^]

**Scheme 45:**
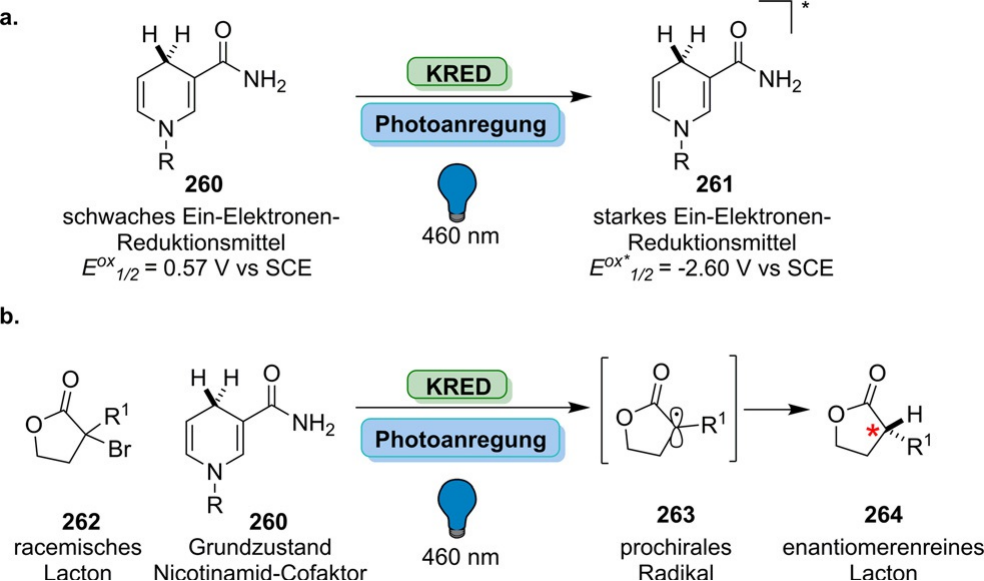
a) Photoanregung des NAD(P)H‐Cofaktors in ein starkes Ein‐Elektronen‐Reduktionsmittel. b) Anwendung des angeregten NAD(P)H für die radikalische Dehalognierung von α‐Halogenlacton **262** zur Synthese des chiralen Lactons **264**.

In Ergänzung zu ihrer Bedeutung für die Oxyfunktionalisierung sind P450‐Enzyme attraktive Hilfsmittel für photobiokatalytische Reaktionen geworden.^[249]^ Vorangegangene Berichte über lichtgetriebene P450‐katalysierte Reaktionen beinhalten die Nutzung des Photosystems I aus Pflanzen zur Hydroxylierung von Tyrosin.^[250]^ Tran und Mitarbeiter verknüpften ein Ru^II^‐Diimin kovalent mit dem Komplex der Häm‐Domäne von P450‐BM3‐Varianten. Dieses Hybridenzym erlaubte die selektive Hydroxylierung C‐terminaler C‐H‐Bindungen von Fettsäuren. Zusätzlich zu hohen Umsätzen und Reaktionsgeschwindigkeiten sind im Falle des Photo‐Biokatalysators die Reduktase und der NAD(P)H‐Cofaktor nicht notwendig, was den Gesamtprozess im Vergleich zur nativen Reaktion deutlich optimiert.^[251]^

Huang et al. nutzten das lichtgetriebene Redoxpotential des Flavin‐Mononucleotids für die enzymkatalysierte intermolekulare Alkylierung terminaler Alkene (**266**) (Schema 46). Somit wurden chirale γ‐substituierte Carbonylverbindungen (**267**) erzeugt, die in vielen bioaktiven Substanzen, wie z. B. Piperidonen, (+)‐3‐Oxoabolen und (*R*)‐4‐Methoxyalkansäuren, zu finden sind.^[252]^ Zuvor beschriebene Substrat‐tolerante Wildtyp‐“En”‐Reduktasen (EREDs) wurden als Biokatalysatoren auf ein breites Spektrum von α‐Halogencarbonylen angewendet. Diese Verbindungen gehen Elektronen‐Donor‐Akzeptor‐Komplexe mit dem reduzierten FMN im aktiven Zentrum ein, das durch sichtbares Licht angeregt wird, um die radikalische Alkylierung zu initiieren. Bemerkenswert ist, dass hervorragende Ausbeuten und Enantioselektivitäten erzielt wurden. Weitere Beispiele in diesem Kontext können in Supporting Section 8 (siehe Hintergrundinformationen) gefunden werden.

**Scheme 46:**
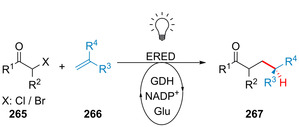
Intermolekulare Alkylierung des terminalen Alkens, katalysiert von EREDs. Abbildung angepasst nach Huang et al.^[252]^

Obwohl sich die Anwendung der Photobiokatalyse im Gebiet der späten Funktionalisierung noch im Anfangsstadium befindet und weitere Fortschritte notwendig sind, um das aktuelle Repertoire zu erweitern, beschreiben die hier dargestellten Studien, dass durch Licht nicht‐natürliche, hochselektive Reaktionen in Enzymen möglich werden, was zahlreiche Möglichkeiten für die selektive Funktionalisierung multifunktionaler Verbindungen bietet.

### 7.2 Glykoengineering

In der Natur sind Zucker häufig an niedermolekulare Verbindungen gebunden,^[253]^ sie sind aber auch wichtige Bestandteile vieler Proteine, wie z. B. in Antikörpern, oder werden an DNA konjugiert und können einen wichtigen Einfluss auf die biologische Aktivität und Stabilität haben.[^254^, ^255^] Zum Beispiel ist 2‐*O*‐α‐d‐Glucopyranosyl‐l‐ascorbat (**268**) ein bioverfügbares und stabiles Derivat der Ascorbinsäure, das von Bedeutung für die Nahrungsmittel‐, Getränke‐ und Kosmetikindustrie ist.^[256]^ Die chemische Konjugation von Glykanen an Kernstrukturen ist eine große Herausforderung, während die Biokatalyse Glykosylierungen im Ein‐Schritt‐Verfahren mit hoher Selektivität und ohne Schutzgruppen ermöglichen kann.^[257]^ Ein typisches Beispiel ist die Generierung des Zuckers **268** direkt aus Ascorbinsäure mithilfe der Cyclodextrin‐Glykosyltransferase (CGTase), welche die Übertragung von α‐Glucose aus Stärke auf eine Reihe von Alkoholen, wie z. B. Ascorbat, regioselektiv und unter Erhalt der Konfiguration am anomeren Zentrum katalysieren kann (Schema 47 a).^[258]^ Die Anwendung der CGTase für die Produktion von Chemikalien ist vorteilhaft gegenüber der weit verbreiteten “Leloir”‐Glykosyltransferase, da die Letztgenannte Zucker‐Nucleotid‐Substrate erfordert, die mithilfe von Enzymkaskaden regeneriert werden können.^[259]^

**Scheme 47:**
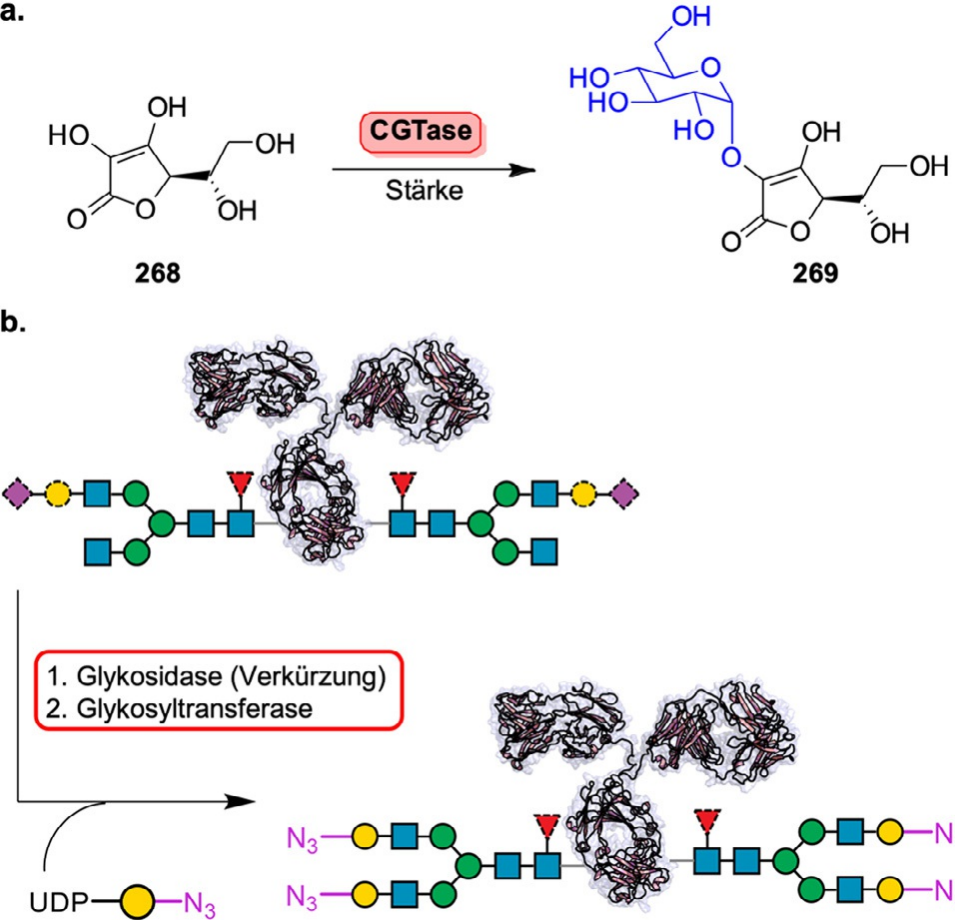
Beispiele für Glykosylierungsreaktionen für die späte Funktionalisierung. a) Enzymatische Glykosylierung niedermolekularer Verbindungen wie Ascorbinsäure; b) enzymatisches Glykoengineering von Biopharmazeutika und die Einführung nicht‐natürlicher bioorthogonaler Gruppen in Glycan‐Ketten von Antikörpern (Glykan‐Nomenklatur ist entsprechend dem zuvor festgelegten Standard gezeigt).^[266]^

Einige elegante Hochdurchsatz‐Screeningmethoden wurden kürzlich entwickelt und ermöglichten die Entdeckung neuer glykosylierender Aktivitäten durch die (Meta‐)Genomik aus umfangreichen exprimierten Bibliotheken bestehend aus Glykosidasen und Glykosynthasen.[^260^, ^261^] Der Umbau von *N*‐Glykanen durch die Biokatalyse findet besondere Beachtung aufgrund breiter Anwendungen für Biopharmazeutika, wie z. B. therapeutische Antikörper und Antikörper‐Wirkstoff‐Konjugate (antibody drug conjugates, ADC). Im Allgemeinen tragen Antikörper ein Asparagin‐gebundenes Oligosaccharid (*N*‐Glycan, Schema 47 b), das essenziell für die Funktion ist. Der Werkzeugkasten der Biokatalysatoren, die entweder die *N*‐Glykan‐Sequenz verkürzen (Endo‐ und Exoglykosidasen) oder spezifisch Zuckermoleküle an die Enden der *N*‐Glykanketten konjugieren, wächst stetig weiter.^[262]^ Die Biokatalyse kann genutzt werden, um natürliche Glycanstrukturen zu erzeugen, wohingegen eine große Anzahl von Enzymen auch eine promiskuitive Funktion besitzt, die es erlaubt, bioorthogonale, nicht‐natürliche Funktionalitäten in Glykoproteine einzubauen. Dies ist insbesondere für die Produktion von ADCs interessant, jedoch auch auf andere Proteine anwendbar.[^263^, ^264^, ^265^]

## 8 Zusammenfassung und Ausblick

Die späte Funktionalisierung ist eine wichtige Säule der modernen organischen Synthesechemie. Es werden intensive Anstrengungen unternommen, um neue Methoden zu entwickeln, die auch von wesentlicher Bedeutung für die Erfolge in der Wirkstoffentwicklung sind. Ein‐Schritt‐Transformationen an komplexen Molekülgerüsten erleichtern Diversifizierungsreaktionen, die wesentlich für die Wirksamkeit sind und wichtige physikochemische Eigenschaften, den Wirkstoffmetabolismus und die Pharmakokinetik steuern.

Enzyme halten zunehmend Einzug in späte Modifizierungen, die sich in vielfältige Richtungen weiterentwickeln. Es ist bereits ein breites Repertoire biokatalytischer Transformationen zugänglich, um Molekülgerüste zu derivatisieren. Jedoch bedingt eine hohe Spezialisierung der Biokatalysatoren oftmals ein eingeschränktes Substratprofil. Fortwährende Anstrengungen werden daher dazu beitragen, diese Schattenseite durch 1) Protein‐Engineering und 2) Sequenzidentifikation aus (Meta‐)Genomen zu verdrängen. Zusätzlich zu der Fülle bereits existierender Beispiele zur molekularen Diversifizierung werden zukünftig weitere enzymkatalysierte Reaktionen folgen. Angesichts der bemerkenswerten Errungenschaften der Biokatalyse in den vergangenen Jahrzehnten wird die Bedeutung später Biotransformationen ohne Zweifel über die kommenden Jahre zunehmen. Es lässt sich erahnen, dass die Biokatalyse eine überragende Rolle in den initialen Stadien der Wirkstoffentwicklung einnehmen kann, da in diesem Bereich die miniaturisierte Synthese in Verbindung mit Hochdurchsatz‐Screenings zunehmend an Bedeutung gewinnen wird.

Es existiert eine nahezu unendliche Anzahl von Biokatalysatoren und orthogonalen Transformationen, die in der Natur genutzt werden, um polyfunktionelle Verbindungen selektiv zu modifizieren. Bis heute haben wir nur an der Oberfläche des Möglichen gekratzt.

## Conflict of interest

Die Autoren erklären, dass keine Interessenkonflikte vorliegen.

## Biographical Information

*Elvira Romero erhielt ihren B.Sc. in Biowissenschaften von der Alcalá University im Jahr 2002. Daraufhin nahm sie ihr M.Sc.‐ (2002) und Ph.D.‐Studium (2010) am Margarita Salas Center for Biological Research (CSIC) auf, wobei sie sich mit lignolytischen Oxidoreduktasen beschäftigte. Als Postdoc untersuchte sie Enzymkinetiken und ‐mechanismen am Virginia Tech und der Georgia State University (2010–2014). Während der folgenden vier Jahre an der University of Groningen konzentrierten sich ihre Postdoc‐Studien auf die Biokatalyse und das Engineering von Enzymen. Seit 2019 ist sie Postdoc in der Biokatalyse‐Gruppe bei AstraZeneca (Schweden)*.

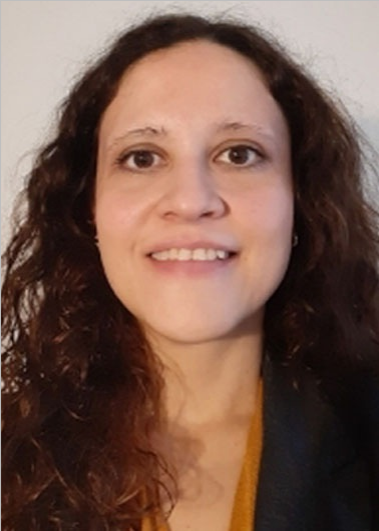

## Biographical Information

*Bethan S. Jones erhielt ihren MSci in Chemie mit Biomedizin vom King's College London im Jahr 2018. Zurzeit ist die Doktorandin an der University of Manchester unter der Anleitung von Prof. Sabine L. Flitsch. Ihre Forschungsarbeiten beschäftigen sich mit der Oxyfunktionalisierung durch P450‐Enzyme. Insbesondere untersucht sie Anwendungen in der frühen und späten Funktionalisierung in biokatalytischen De‐novo‐Kaskaden*.

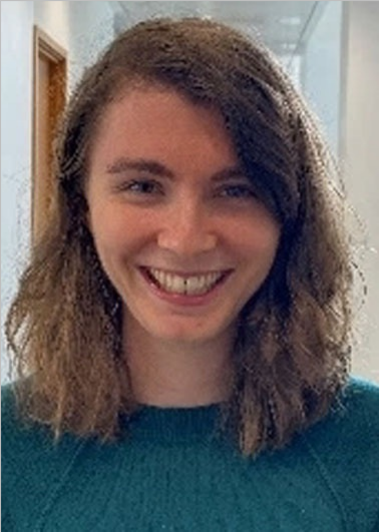

## Biographical Information

*Bethany N. Hogg schloss 2019 ihren MChem in Chemie mit medizinischer Chemie an der University of Manchester ab. Seit 2019 verfolgt sie ihr Ph.D.‐Studium unter der Betreuung von Prof. Nicholas J. Turner. Ihr Projekt befasst sich mit der Synthese niedermolekularer Verbindungen durch Enzymkaskaden; vor allem fokussiert sie sich auf die Öffnung substituierter Epoxide für die Herstellung von 1,2‐bisfunktionalisierten Intermediaten*.

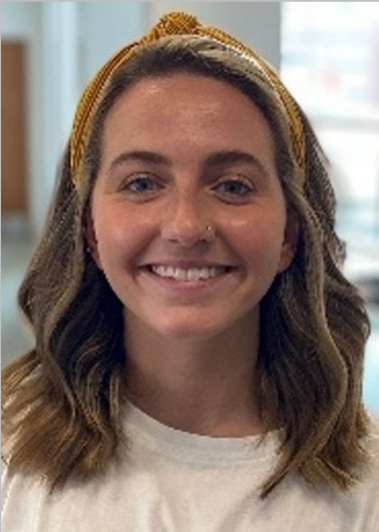

## Biographical Information

*Arnau Rué Casamajo erhielt seinen M.Sc. in molekularer Biotechnologie von der University of Barcelona im Jahr 2019. Für sein Promotionsstudium schloss er sich der Forschungsgruppe von Prof. Nicholas J. Turner an. Der Schwerpunkt seines Projektes ist die biokatalytische Reduktion von Iminen und reduktive Aminierung, wobei er regio‐ und enantioselektive Strategien für die Synthese pharmazeutisch relevanter Substanzen untersucht*.

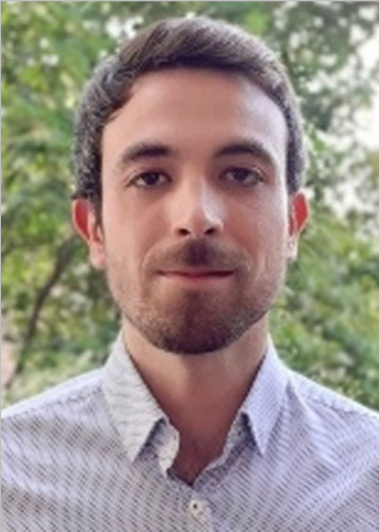

## Biographical Information

*Martin A. Hayes leitet die Abteilung Biokatalyse im iLAB, Discovery Sciences, bei AstraZeneca, Göteborg. Er schloss seinen Ph.D. 1991 bei Prof. T. J. Simpson FRS an der University of Bristol ab. Nach seinem Postdoktorat an der University of Toron mit J. Bryan Jones begann er seine Karriere in der Industrie. Er hat zu der Entwicklung vieler niedermolekularer Verbindungen als Therapeutika beigetragen, z. B. Brilinta und der FLAP‐Inhibitor AZD5718, der sich momentan in Phase 2 der klinischen Studien befindet. Seine Forschungsinteressen umfassen Biokatalyse, Wirkstoffdesign und Hochdurchsatz‐Experimente*.

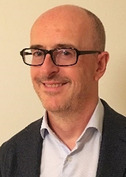

## Biographical Information

*Sabine L. Flitsch ist Professorin an der School of Chemistry der University of Manchester. Sie studierte in Deutschland und erhielt ihr Diplom in Chemie von der WWU Münster. Ihren DPhil schloss sie unter Anleitung von Sir Jack Baldwin an der University of Oxford (Großbritannien) ab. Nach einem dreijährigen Aufenthalt am MIT (USA) bei Prof. H. G. Khorana zog sie zurück nach Großbritannien und bekleidete akademische Positionen an den Universitäten von Oxford und Edinburgh. 2005 wurde sie auf den Lehrstuhl für Chemische Biologie an die University of Manchester berufen*.

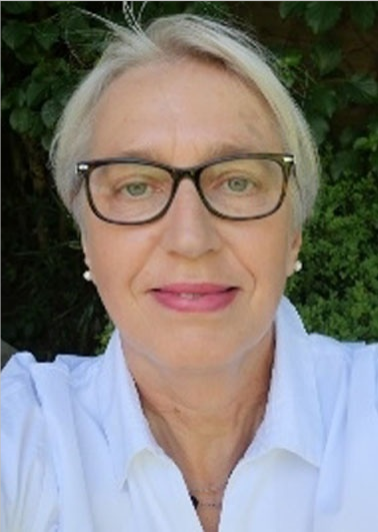

## Biographical Information

*Nicholas J. Turner ist Professor für chemische Biologie am Manchester Institute of Biotechnology (MIB: www.mib.ac.uk) der University of Manchester. Er ist außerdem Direktor des Centre of Excellence in Biocatalysis (CoEBio3, www.coebio3.org). Seine Forschungsinteressen liegen im Bereich der Biokatalyse mit einem besonderen Schwerpunkt auf der Entdeckung und Entwicklung neuer enzymkatalysierter Reaktionen für Anwendungen in der organischen Synthese. Seine Gruppe interessiert sich zudem für Anwendungen der gerichteten Evolution, um Biokatalysatoren mit maßgeschneiderten Funktionen zu entwickeln*.

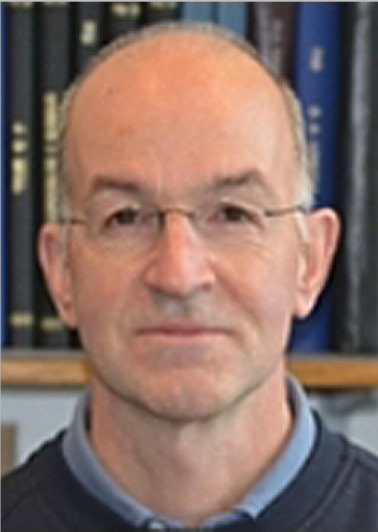

## Biographical Information

*Christian Schnepel studierte Biochemie mit dem Schwerpunkt chemische Biologe an der Universität Bielefeld. 2019 schloss er seine Promotionsarbeit über die biokatalytische Halogenierung bei Prof. Norbert Sewald ab und wurde mit dem Max‐Bergmann‐Nachwuchspreis ausgezeichnet. Momentan ist er Postdoc in der Gruppe von Prof. Nicholas J. Turner am Manchester Institute of Biotechnology (Großbritannien). Seine Forschungsinteressen liegen in der enzymkatalysierten Synthese von Wirkstoffen, insbesondere Methoden zum Aufbau von Amiden und C‐H‐Funktionalisierung*.

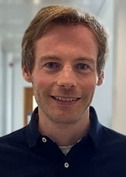

## Supporting information

As a service to our authors and readers, this journal provides supporting information supplied by the authors. Such materials are peer reviewed and may be re‐organized for online delivery, but are not copy‐edited or typeset. Technical support issues arising from supporting information (other than missing files) should be addressed to the authors.

Supplementary
