# Supplementary material for: Enzymkatalysierte späte Modifizierungen: Besser spät als nie
Source: Angew Chem Weinheim Bergstr Ger. 2021 Mar 8;133(31):16962–93. doi: 10.1002/ange.202014931 (PMC10946893; doi:10.1002/ange.202014931)
Supplement: Supplementary file 1 — Supplementary [file ANGE-133-16962-s001.pdf]

Angewandte Chemie
Eine Zeitschrift der Gesellschaft Deutscher Chemiker

Supporting Information

Enzymatic Late-Stage Modifications: Better Late Than Never

Elvira Romero, Bethan S. Jones, Bethany N. Hogg, Arnau Rué Casamajo, Martin A. Hayes, Sabine L. Flitsch, Nicholas J. Turner, and Christian Schnepel**

ange_202014931_sm_miscellaneous_information.pdf

Supporting Content

Supporting Section 1. Strategies towards biocatalyst diversity

1.1. Biocatalyst identification

Diverse enzyme libraries considerably increase the chance of success in the development of target reactions. Enzymes are sourced in many different ways from nature. Traditionally, microorganisms, mainly bacteria and fungi, but also plants and animals served as the source of novel enzymes, often inspired from a known metabolic transformation or a previously identified natural product. Nowadays, microbial strain collections provide laboratories with a large number of different microorganisms. Although this has been a standard strategy applied for several decades, the diversity is rather restricted because of issues such as strain discovery and often unknown cultivation conditions (*e.g.* media, temperature etc.). These approaches are crucial to successfully isolate the gene of interest, enabling its transfer into a heterologous expression host. Today, this 'classical' approach has been overtaken by the huge progress of genomics, especially in DNA sequencing technologies, along with the emergence of bioinformatics. The availability of sequence data in common databases (NCBI and UniProt) provides a vast number of hitherto uncharacterised genes. By making use of *in silico* tools such as alignment, annotation etc., sequence data can be rapidly scanned for characteristic motifs, which permits the prediction of putative functions of the encoded protein. Upon further sequence optimisation, commercial gene synthesis allows access to any sequence of interest that can be subcloned into suitable expression vectors to produce the desired enzyme. Large libraries of homologous genes can be built up to create panels of enzymes, for example in a microtiter plate format. These collections can be screened for a certain transformation of interest that allows the selection of the promising candidate(s), *e.g.,* in terms of activity and selectivity.[1]

Metagenome mining emerged in the past decade providing a vast increase in available sequence space. In general, a metagenome is defined as the collection of all genetic material found in a specific environment or biotope. Without previous cultivation of the individual organisms in the sample taken, the DNA is directly extracted and amplified to determine the sequence. With the aid of bioinformatic tools the resulting metagenomic data are processed and gathered containing a multitude of sequences attributed to a particular environment. Apparently, the composition of metagenomes strongly depends on where the DNA was originally isolated, thus introducing immense variety. Intriguingly, this opens access to a seemingly endless number of new biocatalysts at the same time, so that individual cultivation of the original hosts becomes unnecessary. As screening sequences *in silico* becomes more attractive, genes of interest can be filtered from the metagenome and then optimised for expression leading to rapid enzyme identification.[2] Gene synthesis and cloning give simple access to panels of unprecedented enzymes from metagenomic sources. Hence, metagenomics is regarded as one of the modern key technologies towards increased access to sequence space for biocatalysis that will enhance the number of catalysts amenable for synthetic purposes.

1.2. Enzyme engineering

Enzyme engineering allows for the optimisation of a biocatalyst towards a reaction of interest: Expanding the substrate scope, switching selectivity, higher efficiency, as well as improving the thermo- and solvent stability are typical optimisation goals.[3] Nowadays various engineering methods are available to address these objectives. The choice of a suitable engineering strategy to tailor an enzyme is most crucial to succeed and was discussed in depth by Reetz and co-authors.[4] Among the variety of approaches applied today, directed evolution, as pioneered by Frances Arnold and others, is a very powerful technique.[5] In short, the gene of interest is randomly mutated using error-prone PCR (epPCR), DNA shuffling or mutator strains.[6] Individual colonies (mutants) are arrayed in multi-well plates, usually in a 96- or 384- well format and cultivated. The libraries are then screened for the improved feature of interest, for example conversion of a non-native substrate. Positive hits are finally identified by DNA sequencing to reveal the mutations present. Combination of positive mutations and the use of improved variants as a parent in successive generations finally leads to a highly optimised catalyst. One strength of directed evolution is that mutations can be found, which would otherwise have been difficult to predict. Directed evolution is widely applied in enzyme optimisation in academia and industry with great success. A successful campaign requires persistent screening of $>10^3$ colonies per generation which demands sophisticated screening facilities.

Iterative saturation mutagenesis, also known as a semi-random approach, is particularly useful, and combines insightful structural and mechanistic knowledge with localised random evolution. Selected residues are targeted with a degenerate set of primers so that multiple amino acids are randomly incorporated in a position of interest allowing the best residue to be identified from screening these focused libraries. Combinatorial active site saturation test (CAST) was introduced by Reetz and co-workers and has proven useful in engineering of many enzymes, *e.g*. to improve enantioselectivity.[7] Generation of smart libraries with a limited set of codons reduces screening efforts and cooperative effects can be taken into account.[8]

In addition, the incorporation of non-canonical amino acids (ncAAs) enables expanding the set of native functionalities. Consequently, insertion of ncAAs can have a fundamental impact on the catalyst properties and novel functions can arise. Currently, this area is restricted to model examples, whilst a broader use in synthesis still needs to be demonstrated.[9,10]

In contrast to random-based engineering, rational design relies on computational approaches; especially molecular docking and molecular dynamics (MD) simulations support the prediction of site-directed mutations that help to tailor an enzyme. With the rise of computational tools and capabilities targeted engineering has become more useful recently, due to the reduction of required screening effort. Notably, rational design requires a thorough understanding of the catalytic mechanism, especially in terms of substrate binding and reaction trajectory. Great success has been made in *de novo* protein design where a protein scaffold and its sequence are designed computationally to catalyse a new reaction. The catalytic entity is a totally artificial protein that can be of great

utility when low promiscuity towards a reaction cannot be found in native enzymes. A rather sophisticated process has to be followed including several iterative rounds of design *in silico* as reviewed by Vaissier-Welborn and Head-Gordon.[11] In many cases this starts from active site modelling that is based on the reaction trajectory of interest to generate a so-called 'theozyme'. Subsequently, this theoretical active site has to be placed into a protein scaffold. Usually existing structures serve as templates that are further refined towards the desired transformation using the powerful RosettaDesign algorithm.[12] The groups of Baker and Hilvert, among others, have made outstanding contributions to this field. The design of a retroaldolase was one of the earliest examples of enzyme generation from scratch albeit resulting in modest activity.[13] Work on a *de-novo*-Kemp eliminase that gave rise to a highly optimised catalyst after multiple rounds of evolution illustrates the joint approach of enzyme design and engineering.[14,15] In a similar fashion, an aldolase initially designed *in silico* was considerable improved by means of combined mutagenesis strategies along with microfluidic-based screening, indicating the power of fully rational approaches with ultra-high-throughput engineering.[16–18]

Ancestral sequence reconstruction (ASR) has recently come to the foreground as an innovative method to engineer enzymes. This process goes backwards in natural evolution and ancestral genes are resurrected as artificially designed constructs using *in silico* tools.[19] More robust biocatalysts can arise, *e.g.* equipped with increased temperature and solvent tolerance or a broader substrate scope. Artificial sequences are generated from phylogenetic analyses of extant sequences that lead to artificial ancestral sequences at phylogenetic branch points (nodes). Resurrected sequences introduce new diversity to deduce relationships between sequence and function. Additionally, more robust ancestors often provide useful starting points for subsequent evolution campaigns. Using ASR, Gillam *et al.* engineered vertebrate cytochrome P450 monooxygenase (P450) enzymes and a ketol-acid reductoisomerase (KARI) towards higher stability. The corresponding ancestors exhibited ~30 °C higher thermostability than the extant P450 and KARI.[20] Likewise, ancestral transaminases and carboxylic acid reductases (CARs) were reported recently, thus indicating the great potential of this approach.[21,22]

High-throughput screening tools are necessary for biocatalyst development, so that enzyme panels can be assayed towards the selection target, *e.g.* the transformation of interest or crucial process parameters. Hence, it is not surprising that both throughput and specificity of the enzyme assay used must be taken into account. Numerous assays have evolved that span a wide area of detection techniques. Undoubtedly, colorimetric or fluorogenic screenings are the most relevant ones with respect to throughput and reliability.[23] Moreover, chromatographic methods like HPLC, UPLC or GC, often coupled with UV and/or MS detection provide universal methods. Continuous development of powerful instruments and a high degree of modularity enable the frequent use of these standard methods in many laboratories, especially in industry, for library screening. Simple and reliable screening of mutant libraries is, for example, feasible using solid-phase assays. Enzyme activity can be directly monitored in bacterial colonies on agar plates or a nylon membrane, where the detection is often based on a peroxidase-coupled assay.[24,25] Recently, MS-based techniques evolved that facilitate ultra-high throughput screening and sensitivity due to the inherent ability of detecting trace quantities by MS. DESI-MS screening allows label free detection of enzyme activity directly in the bacterial cell.[26] Great promise is also expected from microfluidics that set the basis to miniaturised screening platforms while offering an immense throughput. Hollfelder *et al.* demonstrated compartmentalisation of hydrolases along with necessary reagents and substrates in oil droplets on a single cell level that was utilised in the directed evolution of sulfatases achieving a throughput of up to 10^7 variants.[27–29] A recent methodology called 'µscale' occurs on single cells spread on a chip where a CCD camera detects a fluorescence signal on a picolitre scale.[30] However, further improvement and simplification of these methods are required to become more frequently applied techniques in enzyme evolution.

Supporting Section 2. C-H Bond activation

Heterolytic proton cleavage formally leads to C^- and H^+ species, while the tendency of deprotonation strongly depends on the stability of the resulting carbanion. Aldol[31] and *umpolung*[32] reactions are well-known examples in chemistry and biocatalysis where a deprotonation event triggers activation to finally end up with a C-C bond. In the opposite way, hydride removal (H^-) is also possible; a reaction typically observed for dehydrogenation reactions in biocatalysis, with the recent development of self-sufficient hydrogen transfer reaction as a redox-neutral alternative.[33] Homolytic hydrogen abstraction is a common principle capable of addressing aliphatic bonds with the bond dissociation energy as the crucial metric indicating the tendency to form a $C^{\bullet}$ radical. For instance, in P450 enzymes this is achieved by the hypervalent iron(IV)-oxo species.[34]

Table S1. Bond dissociation energies of typical C-H bonds. The hydrogen atom of interest is marked in bold. Values were adopted from Xue *et al*.[35] Abbreviations: Alk: alkyl; Cyhex: cyclohexyl. The examples mentioned here depict representatives of typical hydrocarbons. BDE: Bond dissociation energy.

Type of hydrocarbon	Examples	BDE / kcal mol^{-1}
Methyl	H_3C−**H**	105
Alkane (primary)	Alk−CH_2-**H**	95-100
Alkane (secondary)	Alk−CH**H**-Alk	98
Alkane (tertiary)	Alk$_3$C−**H**	96
Cycloalkane	Cyhex−**H**	99
Alkene	RC=CH−**H**	111-114
Aromatic	Ph−**H**	112
Allene	H_2C=C=CH−**H**	111
Alkyne	R−C≡C−**H**	133

Supporting Section 3. Background on biocatalytic oxyfunctionalisation

3.1. P450s

P450s are ubiquitous haem-thiolate proteins involved in natural product biosynthesis and xenobiotic metabolism. P450s catalyse numerous reactions: hydroxylation, epoxidation, decarboxylation, oxidation of heteroatoms, dealkylation, C-C-bond cleavage, nitration, phenolic coupling and Baeyer-Villiger oxidation.[36] Remarkably, even non-natural chemistries (*e.g.* alkylation, fluoroalkylation and amination) can be achieved using engineered P450 variants, as shown in a recent review article.[37] There is a long and expanding list of P450 substrates which includes fatty acids, alkaloids, terpenoids, polyenes and macrolides which are attractive for C-H functionalisation.[38]

Given the enormous functional diversity found within the P450 superfamily, it is not surprising that these enzymes are of great interest in synthetic chemistry, especially in API synthesis. This extraordinary ability to regio- and stereoselectively oxyfunctionalise unactivated C-H bonds in complex molecules is especially attractive for LSF of drug scaffolds. Importantly, the increasing development of thermostable P450s has been driven initially *via* directed evolution[39] and more recently by ASR.[20]

Additionally, the more recent identification of P450s from thermophilic organisms (*e.g.* CYP505A30 from *Thermothelomyces thermophila*[40,41] and CYP116B46 from *Tepidiphilus thermophilus*)[42] provide a broader panel of industrially viable enzymes due to their stability and self-sufficiency. These self-sufficient enzymes (*i.e.* Class VII and VIII P450s) contain both the haem and reductase domains (Figure S1). Thus they do not need additional redox partners to enable the two single electron transfers to the haem domain during the catalytic cycle.[43] The best-studied natural self-sufficient, Class VIII, P450 is from *Bacillus megaterium* (CYP102A1, P450-BM3).[44]

Moreover, artificial fusion proteins containing both reductase and haem domains have been successfully constructed and implemented.[45] Notably, enzyme-based and alternative approaches for NAD(P)H regeneration in the P450 reactions are already well established.[36] These unique qualities of P450s make them ideal catalysts for LSF.

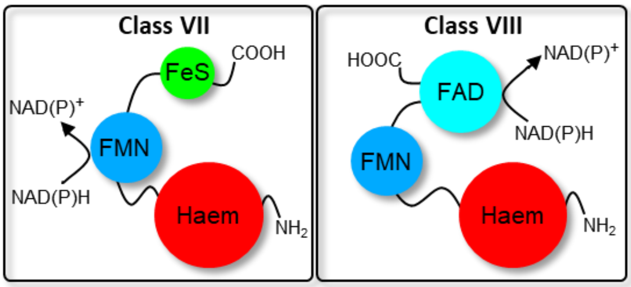

Figure S1. Schematics of self-sufficient Class VII and VIII P450s.

3.2. Peroxygenases

Unspecific peroxygenases (UPOs) perform highly selective C-H oxyfunctionalisations using hydrogen peroxide as both oxygen donor and final electron acceptor.[46] These fungal extracellular glycoproteins belong to the haem-thiolate protein superfamily, which also includes cytochrome P450s. Many studies have demonstrated the ability of UPOs to catalyse a broad array of reactions including hydroxylation, epoxidation, halogenation, sulfoxidation, *N*-oxidation and dealkylation.[47] Importantly, UPOs exhibit remarkable stability at relatively high temperature and also in the presence of co-solvents. These facts have motivated numerous efforts to implement native and engineered UPOs in different industrial processes. Indeed, UPOs have been described as “dream catalysts” of the future in a review article published in 2017.[47] At the end of 2019, an online database called the ‘Unspecific Peroxygenase Database’ (UPObase) was created which includes approximately 2000 UPO sequences.[48] ‘Hydrogen peroxide driven biocatalysis’ was recently reviewed in 2019, by Burek *et al.* highlighting the significance of UPOs for oxyfunctionalisation.[49] Thus the breadth of new UPOs is ever expanding for the identification of viable LSF biocatalysts.

3.3. Flavin-dependent monooxygenases

Unlike P450s and UPOs, flavin-dependent monooxygenases (FMOs) are non-haem thiolate enzymes, and instead require either flavin adenine dinucleotide (FAD) or flavin mononucleotide (FMN) for catalysis.[50] This vital active site cofactor can either be covalently or non-covalently bound (Figure 2b). Its reduction enables the activation of dioxygen, utilising NAD(P)H as the electron donor.[51] Recently, it has been shown to function in combination with a nicotinamide coenzyme biomimetic, leading to lower levels of uncoupling.[52]

Prominently FMOs catalyse aromatic hydroxylations, epoxidations and Bayer-Villiger oxidations, yet are also capable of oxidative decarboxylation, oxidative denitration, halogenation, desulfurisation, sulfoxidation, and *N*-hydroxylation.[53–55] Late-stage FMO catalysed regio- and stereoselective aromatic hydroxylation is prominent within many natural product pathways, thus making FMOs an essential biocatalyst for late-stage enzymatic oxyfunctionalisation.[56–58]

3.4. Iron/α-ketoglutarate-dependent hydroxylases

Iron/α-ketoglutarate-dependent hydroxylases (Fe/αKGs), are non-haem dependent hydroxylases that utilise dioxygen and α-ketoglutarate (αKG), *via* radical-based C-H abstraction in the active site (Figure 2c).[59] These cofactors are more abundant and cheaper than NAD(P)H; additionally no further reductase partners are required for catalysis. This fact and the ability to catalyse a plethora of reactions, *i.e.* hydroxylation, epoxidation, ring formation, desaturation, epimerisation, and dealkylation,[60,61] render Fe/αKG-dependent hydroxylases advantageous over P450s.

Importantly, Fe/αKGs are also key enzymes within many different natural product biosynthetic pathways mainly hydroxylating amino acid building blocks. These enzymes were reviewed by Renata *et al.* in the beginning of 2020,[59] highlighting their diversity and viability as biocatalyst within the natural world for oxyfunctionalisation.

3.5. Non-haem diiron monooxygenases

Non-haem diiron monooxygenases are an alternative family of enzymes for C-H bond oxidation. In the active site (Figure 2d) two iron atoms are coordinated to His and Glu residues containing bridging oxygen atoms.[62] This unique subset of monooxygenases is mainly capable of catalysing hydroxylation reactions, but has also been shown to perform aromatic hydroxylations, desaturation reactions and the oxidation of aminoarenes to nitroarenes,[62,63] as part of a diverse class of monooxygenases involved in oxyfunctionalisation in natural product biosynthesis.[63]

3.6. Rieske non-haem iron-dependent oxygenases

Rieske non-haem iron-dependent oxygenases (Rieske oxygenases) are another distinctive class of multicomponent enzymes. The active site (Figure 2e) consists of a non-haem iron centre subunit coordinated to His and Asp residues, which are bridged *via* a conserved Asp residue to a signature Rieske iron-sulfur cluster, vital for the transfer of electrons.[58,64] These unique Rieske clusters can vary across enzymes from [Fe-S], to [2Fe-2S] or [4Fe-4S] moieties.[65] Principally, Rieske oxygenases perform aromatic C-H dihydroxylations, yet are also capable of monohydroxylation, demethylation, amine oxidation and oxidative cyclisation.[64] They facilitate a vital role in the oxidative degradation of aromatic compound pathways and natural product biosyntheses.[58,64–66]

Supporting Section 4. Biocatalytic halogenation mechanisms

Haloperoxidases and Fl-Hals form hypohalous acid as the halogenating agent. Haloperoxidases release HOX into the medium which leads to non-selective halogenation. On the contrary, Fl-Hals proceed *via* a more selective mechanism, where HOX is shuttled within the enzyme passing through a 10 Å tunnel and enabling a regioselective reaction to take place within the protein that was extensively examined for tryptophan (Trp) halogenases.[67] A Lys residue (Lys79 in Trp halogenases) facing the substrate was shown to be crucial for activity, potentially by the intermediary formation of a covalent Lys *N*ε-chloroamine.[68–70] Fe/αKG-Hals resemble their corresponding hydroxylases. Driven by the oxidative decarboxylation of α-ketoglutarate (α-KG), a ferryl-oxo species initiates a radical-based halogen transfer through formation of a substrate radical located proximally to the metal complex.[71,72] This is followed by recombination with a halogen, thus resulting in the halogenated product. Mitchell *et al.* postulated a re-positioning of the oxo-ligand of the haloferryl complex into the plane as the only plausible way to explain the enzyme's regioselectivity.[73,74]

Only a few fluorinases are currently known and have a rather specialised substrate scope.[75,76] In contrast to other halogenases, fluorinations proceed in a nucleophilic fashion according to an S_N2 type mechanism, since an oxidative mechanism is utterly excluded due to the low oxidation potential of fluoride (Figure 13d). Moreover, desolvation of fluoride is essential for it to act as a nucleophile so that the loss of hydrogen bonding interactions must be compensated by the enzyme. *S*-adenosyl-L-methionine (AdoMet) serves as a fluoride acceptor that is converted into 5-fluoro-5-deoxyadenosine (5′-FDA) and L-methionine (L-Met).

The high diversity of halogenases is also related to pronounced differences in terms of substrate scope. Haloperoxidases and Fl-Hals predominantly act on electron-rich compounds, *e.g.* aromatics and heteroaromatics, such as indoles, pyrroles, and phenols. Instead, Fe/αKG-Hals offer the possibility to access less activated substrates, *e.g.* aliphatic moieties.

Supporting Section 5. Enzymatic methylation

Supporting Section 6. Amide bond formation using hydrolases

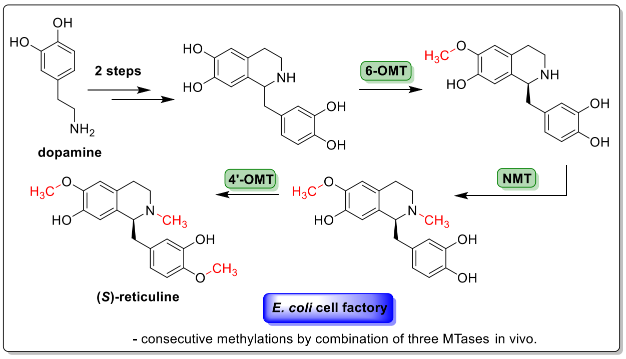

Scheme S1. Stepwise methylation affords (S)-reticuline in vivo. By performing three consecutive methylation steps in the E. coli cell, AdoMet regeneration merely relies on the cell metabolism, thus circumventing the common bottleneck of cofactor supply.

6.1. Lipases

In Nature, lipases catalyse the hydrolysis of fatty acid esters. Decades ago researchers found that these hydrolases are also capable of aminolysis in the absence of water to afford amides (Scheme 36). Usually lipase-catalysed reactions are carried out in non-aqueous, apolar solvents resembling the native environment of the lipase. In principle, water must be excluded from the active site, so that the acyl-enzyme intermediate can be attacked by the amine instead. Certainly, the *Candida antarctica* lipases (CAL-A, CAL-B) are the best examined members and were widely used for this purpose, for example, in kinetic resolutions.[77] On the other hand, direct acylation of amines using carboxylic acids rather than more activated esters is more challenging and reported in fewer studies. However, this more direct approach is desirable, since yield-limiting and less atom economic esterifications are circumvented.

Sheldon and co-workers were among the first reporting on an entirely enzyme-catalysed amidation using a lipase *via* intermediate ester formation.[78] Several reports followed afterwards; in 2018, Manova *et al.* established a process towards the use of immobilised CAL-B for direct amidation performed in 1,4-dioxane at elevated temperatures. Among the wide substrate scope reported, lipoic acid was coupled to different amine nucleophiles.[79] Recently, Testera *et al.* contributed to biopolymer derivatisation with the aid of a lipase: CAL-B was used to modify glutamic acid side chains of artificial

elastin-like molecules by coupling different amines to the free carboxylate forming novel conjugates.[80]

A notable step forward in direct amidation using lipases was achieved by Zeng *et al.* The researchers identified an intracellular lipase, SpL, from a *Sphingomonas* strain.[81]

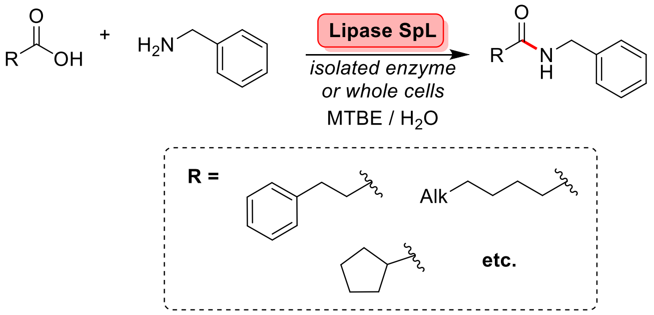

Scheme S2. Direct amidation of carboxylic acids by the lipase SpL. A diverse scope of acids can be coupled, even in biphasic mixtures of MTBE and water.

Using the whole-cell catalyst in a mixture of methyl *tert*-butyl ether and water, carboxylic acids could be directly transformed into amides, indicating that anhydrous conditions were not essential (Scheme S2). Although a much broader substrate scope was demonstrated for the aminolysis of methyl esters, the findings indicate a more rapid amidation when using the carboxylic acid. Potentially, SpL can serve as an initial model catalyst that facilitates the identification of related homologues. Furthermore, structural elucidation of the mechanism will also aid in expanding the substrate range towards lipase-catalysed, cofactor-free amidation.

6.2. Penicillin acylases

Penicillin acylases (PAs) are predominantly applied to access semi-synthetic penicillins and cephalosporins in the pharmaceutical industry. In the amidation direction, PAs catalyse the condensation of 6-aminopenicillanic acid and phenylacetic acid yielding penicillin G.[82] Penicillin synthesis is a pivotal process in medicinal chemistry for obtaining novel antibiotics by altering the side chain of the β-lactam scaffold. Successful multi-ton production of antibiotics is the result of extensive process optimisations, *e.g.* making use of immobilisation.[83] Hence it is not surprising that PA has been a target for protein engineering to boost its application in industrial processes.[84] PAs are endowed with broad promiscuity, thus allowing for variation of the acyl donor moiety. Substituted phenylacetic acids and few aliphatic acids are tolerated by PAs whilst the affinity to the native substrate prevails. Already early studies indicated a reasonably broad amine scope. It is particularly attractive for synthetic means that even amino acid ester or smaller peptides, *e.g.* carrying an N-terminal glycine residue, could adopt the role of 6-aminopenicillanic acid.[85] This property was exploited for peptide synthesis applying PA for the enzymatic cleavage of the phenylacetyl and benzyloxycarbonyl groups that serve as N-terminal protecting groups.[86,87] Furthermore, synthesis of dipeptides using PA was feasible in water; coupling of phenylglycine to a range of representative amino acids was demonstrated yielding the corresponding diketopiperazines upon cyclisation (Scheme S3).[88]

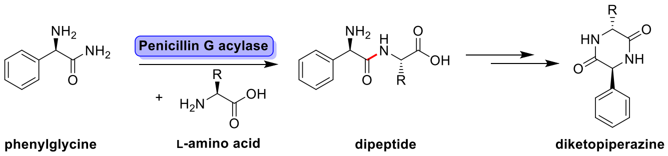

Scheme S3. Penicillin G-acylase enables coupling of L-amino acids (**210**) resulting in different dipeptides (**211**). Further chemical steps can give access to diketopiperazines (**212**).

Although PAs are all-important in the pharmaceutical industry their use for late-stage modification is still in its infancy most likely due to the narrow acid scope accepted.

Supporting Section 7. Reduction of double bonds

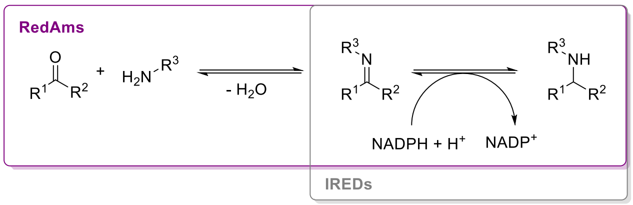

Scheme S4: Reductive aminases (RedAms) and imine reductases (IREDs) reaction scheme.

Supporting Section 8. Photobiocatalysis

The oxidation of water by a light-driven titanium dioxide (TiO_2)-based photocatalyst was applied to the selective conjugated C=C-double bond reduction of ketoisophorone using the OYE homologue from *Thermus scotoductus* SA-01 (*Ts*OYE).[89] FMN delivered electrons from the photocatalyst to the enzyme to saturate the substrate C=C double bond yielding (*R*)-levodione. This transformation was achieved with 66% conversion and 86% enantioselectivity (Scheme S4).

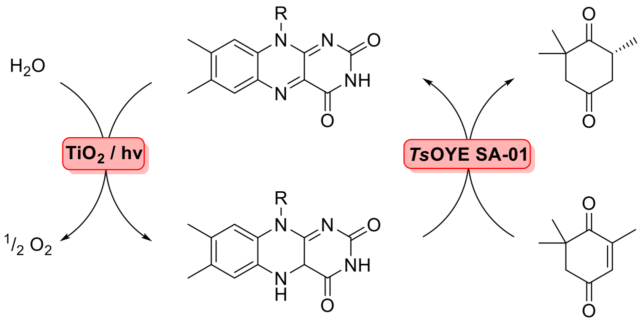

Scheme S4. Photo mediated hydrogenation of ketoisophorone to *R*-levodione by *Ts*OYE.

Formate oxidation catalysed by formate dehydrogenase provides NADH, which can be used by various visible light-driven photocatalysts (*e.g.* phenosafranine, methylene blue or FMN) to generate hydrogen peroxide for peroxygenase-catalyzed hydroxylation of ethylbenzene to (*R*)-1-phenylethanol.[90]

References

[1] A. Zaparucha, V. De Berardinis, C. Vaxelaire-Vergne, in *RSC Catal. Ser.*, Royal Society Of Chemistry, **2018**, pp. 3–27.

[2] D. Baud, J. W. E. Jeffries, T. S. Moody, J. M. Ward, H. C. Hailes, *Green Chem.* **2017**, *19*, 1134–1143.

[3] U. Bornscheuer, *Synlett* **2012**, *24*, 150–156.

[4] G. Qu, A. Li, C. G. Acevedo-Rocha, Z. Sun, M. T. Reetz, *Angew. Chem. Int. Ed.* **2020**, *59*, 13204–13231.

[5] F. H. Arnold, *Angew. Chem. Int. Ed.* **2019**, *58*, 14420–14426.

[6] N. J. Turner, *Nat. Chem. Biol.* **2009**, *5*, 567–573.

[7] M. T. Reetz, L. W. Wang, M. Bocola, *Angew. Chem. Int. Ed.* **2006**, *45*, 1236–1241.

[8] M. T. Reetz, S. Prasad, J. D. Carballeira, Y. Gumulya, M. Bocola, *J. Am. Chem. Soc.* **2010**, *132*, 9144–9152.

[9] I. Drienovská, G. Roelfes, *Nat. Catal.* **2020**, *3*, 193–202.

[10] J. Zhao, A. J. Burke, A. P. Green, *Curr. Opin. Chem. Biol.* **2020**, *2020*, 136–144.

[11] V. Vaissier Welborn, T. Head-Gordon, *Chem. Rev.* **2019**, *119*, 6613–6630.

[12] F. Richter, A. Leaver-Fay, S. D. Khare, S. Bjelic, D. Baker, *PLoS One* **2011**, *6*, e19230.

[13] L. Jiang, E. A. Althoff, F. R. Clemente, L. Doyle, D. Röthlisberger, A. Zanghellini, J. L. Gallaher, J. L. Betker, F. Tanaka, C. F. Barbas, D. Hilvert, K. N. Houk, B. L. Stoddard, D. Baker, *Science (80-.).* **2008**, *319*, 1387–1391.

[14] H. K. Privett, G. Kiss, T. M. Lee, R. Blomberg, R. A. Chica, L. M. Thomas, D. Hilvert, K. N. Houk, S. L. Mayo, *Proc. Natl. Acad. Sci.* **2012**, *109*, 3790–3795.

[15] R. Blomberg, H. Kries, D. M. Pinkas, P. R. E. Mittl, M. G. Grütter, H. K. Privett, S. L. Mayo, D. Hilvert, *Nature* **2013**, *503*, 418–421.

[16] X. Garrabou, T. Beck, D. Hilvert, *Angew. Chem. Int. Ed.* **2015**, *54*, 5609–5612.

[17] L. Giger, S. Caner, R. Obexer, P. Kast, D. Baker, N. Ban, D. Hilvert, *Nat. Chem. Biol.* **2013**, *9*, 494–498.

[18] R. Obexer, A. Godina, X. Garrabou, P. R. E. Mittl, D. Baker, A. D. Griffiths, D. Hilvert, *Nat. Chem.* **2017**, *9*, 50–56.

[19] Y. Gumulya, E. M. J. Gillam, *Biochem. J.* **2017**, *474*, 1–19.

[20] Y. Gumulya, J. M. Baek, S. J. Wun, R. E. S. Thomson, K. L. Harris, D. J. B. Hunter, J. B. Y. H. Behrendorff, J. Kulig, S. Zheng, X. Wu, B. Wu, J. E. Stok, J. J. De Voss, G. Schenk, U. Jurva, S. Andersson, E. M. Isin, M. Bodén, L. Guddat, E. M. J. Gillam, *Nat. Catal.* **2018**, *1*, 878–888.

[21] M. Wilding, T. S. Peat, S. Kalyaanamoorthy, J. Newman, C. Scott, L. S. Jermiin, *Green Chem.* **2017**, *19*, 5375–5380.

[22] A. Thomas, R. Cutlan, W. Finnigan, M. van der Giezen, N. Harmer, *Commun. Biol.* **2019**, *2*, 1–12.

[23] H. Xiao, Z. Bao, H. Zhao, *Ind. Eng. Chem. Res.* **2015**, *54*, 4011–4020.

[24] M. S. Weiß, I. V Pavlidis, C. Vickers, M. Höhne, U. T. Bornscheuer, *Anal. Chem.* **2014**, *86*, 11847–11853.

[25] F. Parmeggiani, S. L. Lovelock, N. J. Weise, S. T. Ahmed, N. J. Turner, *Angew. Chem. Int. Ed.* **2015**, *54*, 4608–4611.

[26] C. Yan, F. Parmeggiani, E. A. Jones, E. Claude, S. A. Hussain, N. J. Turner, S. L. Flitsch, P. E. Barran, *J. Am. Chem. Soc.* **2017**, *139*, 1408–1411.

[27] M. Kaltenbach, F. Hollfelder, *Methods Mol. Biol.* **2012**, *805*, 101–111.

[28] B. Kintses, C. Hein, M. F. Mohamed, M. Fischlechner, F. Courtois, C. Lainé, F. Hollfelder, *Chem. Biol.* **2012**, *19*, 1001–1009.

[29] P. Y. Colin, B. Kintses, F. Gielen, C. M. Miton, G. Fischer, M. F. Mohamed, M. Hyvönen, D. P. Morgavi, D. B. Janssen, F. Hollfelder, *Nat. Commun.* **2015**, *6*, 10008.

[30] B. Chen, S. Lim, A. Kannan, S. C. Alford, F. Sunden, D. Herschlag, I. K. Dimov, T. M. Baer, J. R. Cochran, *Nat. Chem. Biol.* **2016**, *12*, 76–81.

[31] N. G. Schmidt, E. Eger, W. Kroutil, *ACS Catal.* **2016**, *6*, 4286–4311.

[32] C. Dresen, M. Richter, M. Pohl, S. Lüdeke, M. Müller, *Angew. Chem. Int. Ed.* **2010**, *49*, 6600–6603.

[33] E. Tassano, M. Hall, *Chem. Soc. Rev.* **2019**, *48*, 5596–5615.

[34] F. Hollmann, D. J. Opperman, C. E. Paul, *Angew. Chem. Int. Ed.* **2020**, DOI 10.1002/anie.202001876.

[35] X. S. Xue, P. Ji, B. Zhou, J. P. Cheng, *Chem. Rev.* **2017**, *117*, 8622–8648.

[36] V. B. Urlacher, M. Girhard, *Trends Biotechnol.* **2019**, *37*, 882–897.

[37] K. Chen, F. H. Arnold, *Nat. Catal.* **2020**, *3*, 203–213.

[38] L. M. Podust, D. H. Sherman, *Nat. Prod. Rep.* **2012**, *29*, 1251–1266.

[39] O. Salazar, P. C. Cirino, F. H. Arnold, *ChemBioChem* **2003**, *4*, 891–893.

[40] M. J. L. J. Fürst, B. Kerschbaumer, C. Rinnofner, A. K. Migglautsch, M. Winkler, M. W. Fraaije, *Adv. Synth. Catal.* **2019**, *361*, 2487–2496.

[41] G. J. Baker, H. M. Girvan, S. Matthews, K. J. McLean, M. Golovanova, T. N. Waltham, S. E. J. Rigby, D. R. Nelson, R. T. Blankley, A. W. Munro, *ACS Omega* **2017**, *2*, 4705–4724.

[42] M. Tavanti, J. L. Porter, S. Sabatini, N. J. Turner, S. L. Flitsch, *ChemCatChem* **2018**, *10*, 1042–1051.

[43] Z. Li, Y. Jiang, X. F. P. Guengerich, L. Ma, S. Li, W. Zhang, *J. Biol. Chem.* **2020**, *295*, 833–849.

[44] C. J. C. Whitehouse, S. G. Bell, L.-L. L. Wong, *Chem. Soc. Rev.* **2012**, *41*, 1218–1260.

[45] S. J. Sadeghi, G. Gilardi, *Biotechnol. Appl. Biochem.* **2013**, *60*, 102–110.

[46] M. Hofrichter, R. Ullrich, *Curr. Opin. Chem. Biol.* **2014**, *19*, 116–125.

[47] Y. Wang, D. Lan, R. Durrani, F. Hollmann, *Curr. Opin. Chem. Biol.* **2017**, *37*, 1–9.

[48] M. Faiza, D. Lan, S. Huang, Y. Wang, *Database (Oxford).* **2019**, *2019*, 1–15.

[49] B. O. Burek, S. Bormann, F. Hollmann, J. Z. Bloh, D. Holtmann, *Green Chem.* **2019**, *21*, 3232–3249.

[50] M. M. E. Huijbers, S. Montersino, A. H. Westphal, D. Tischler, W. J. H. Van Berkel, *Arch. Biochem. Biophys.* **2014**, *544*, 2–17.

[51] J. Sucharitakul, R. Tinikul, P. Chaiyen, *Arch. Biochem. Biophys.* **2014**, *555–556*, 33–46.

[52] A. Guarneri, A. H. Westphal, J. Leertouwer, J. Lunsonga, M. C. R. Franssen, D. J. Opperman, F. Hollmann, W. J. H. van Berkel, C. E. Paul, *ChemCatChem* **2020**, *12*, 1368–1375.

[53] J. Rebehmed, V. Alphand, V. De Berardinis, A. G. De Brevern, *Biochimie* **2013**, *95*, 1394–1402.

[54] M. L. Mascotti, M. Juri Ayub, N. Furnham, J. M. Thornton, R. A. Laskowski, *J. Mol. Biol.* **2016**, *428*, 3131–3146.

[55] P. Chenprakhon, T. Wongnate, P. Chaiyen, *Protein Sci.* **2019**, *28*, 8–29.

[56] C. Hertweck, A. Luzhetskyy, Y. Rebets, A. Bechthold, *Nat. Prod. Rep.* **2007**, *24*, 162–190.
[57] Y. Matsuda, I. Abe, *Nat. Prod. Rep.* **2016**, *33*, 26–53.
[58] E. King-Smith, C. R. Zwick, H. Renata, *Biochemistry* **2018**, *57*, 403–412.
[59] C. R. Zwick, H. Renata, *Nat. Prod. Rep.* **2020**, *37*, 1065–1079.
[60] S. Martinez, R. P. Hausinger, *J. Biol. Chem.* **2015**, *290*, 20702–20711.
[61] C. Peters, R. M. Buller, *Catalysts* **2019**, *9*, DOI 10.3390/catal9030221.
[62] A. J. Jasniewski, L. Que, *Chem. Rev.* **2018**, *118*, 2554–2592.
[63] A. J. Komor, A. J. Jasniewski, L. Que, J. D. Lipscomb, *Nat. Prod. Rep.* **2018**, *35*, 646–659.
[64] S. M. Barry, G. L. Challis, *ACS Catal.* **2013**, *3*, 1–7.
[65] C. Perry, E. L. C. De Los Santos, L. M. Alkhalaf, G. L. Challis, *Nat. Prod. Rep.* **2018**, *35*, 622–632.
[66] M. C. Tang, Y. Zou, K. Watanabe, C. T. Walsh, Y. Tang, *Chem. Rev.* **2017**, *117*, 5226–5333.
[67] C. Dong, S. Flecks, S. Unversucht, C. Haupt, K. H. Van Pée, J. H. Naismith, *Science (80-.).* **2005**, *309*, 2216–2219.
[68] E. Yeh, L. C. Blasiak, A. Koglin, C. L. Drennan, C. T. Walsh, *Biochemistry* **2007**, *46*, 1284–1292.
[69] S. Flecks, E. P. Patallo, X. Zhu, A. J. Ernyei, G. Seifert, A. Schneider, C. Dong, J. H. Naismith, K.-H. van Pée, *Angew. Chem. Int. Ed.* **2008**, *47*, 9533–9536.
[70] T. G. Karabencheva-Christova, J. Torras, A. J. Mulholland, A. Lodola, C. Z. Christov, *Sci. Rep.* **2017**, *7*, 17395.
[71] F. H. Vaillancourt, J. Yin, C. T. Walsh, *Proc. Natl. Acad. Sci. U. S. A.* **2005**, *102*, 10111–10116.
[72] L. C. Blasiak, F. H. Vaillancourt, C. T. Walsh, C. L. Drennan, *Nature* **2006**, *440*, 368–371.
[73] M. L. Hillwig, X. Liu, *Nat. Chem. Biol.* **2014**, *10*, 921–923.
[74] A. J. Mitchell, Q. Zhu, A. O. Maggiolo, N. R. Ananth, M. L. Hillwig, X. Liu, A. K. Boal, *Nat. Chem. Biol.* **2016**, *12*, 636–640.
[75] D. O'Hagan, H. Deng, *Chem. Rev.* **2015**, *115*, 634–649.
[76] L. Wu, F. Maglangit, H. Deng, *Curr. Opin. Chem. Biol.* **2020**, *55*, 119–126.
[77] R. N. Lima, C. S. dos Anjos, E. V. M. Orozco, A. L. M. Porto, *Mol. Catal.* **2019**, *466*, 75–105.
[78] M. C. De Zoete, A. C. K. Van Dalen, F. Van Rantwijk, R. A. Sheldon, *J. Chem. Soc. Chem. Commun.* **1993**, 1831–1832.
[79] D. Manova, F. Gallier, L. Tak-Tak, L. Yotava, N. Lubin-Germain, *Tetrahedron Lett.* **2018**, *59*, 2086–2090.
[80] A. M. Testera, M. Santos, A. Girotti, F. J. Arias, J. M. Báñez, M. Alonso, J. C. Rodríguez-Cabello, *Int. J. Biol. Macromol.* **2019**, *121*, 752–759.
[81] S. Zeng, J. Liu, S. Anankanbil, M. Chen, Z. Guo, J. P. Adams, R. Snajdrova, Z. Li, *ACS Catal.* **2018**, *8*, 8856–8865.
[82] A. Brugging, E. C. Roos, E. De Vroom, *Org. Process Res. Dev.* **1998**, *2*, 128–133.
[83] A. I. Kallenberg, F. van Rantwijk, R. A. Sheldon, *Adv. Synth. Catal.* **2005**, *347*, 905–926.
[84] V. I. Tishkov, S. S. Savin, A. S. Yasnaya, *Acta Naturae* **2010**, *2*, 47–61.
[85] A. Pessina, P. Lüthi, P. L. Luisi, J. Prenosil, Y. -S Zhang, *Helv. Chim. Acta* **1988**, *71*, 631–641.
[86] H. Waldmann, *Tetrahedron Lett.* **1988**, *29*, 1131–1134.
[87] G. Alvaro, J. A. Feliu, G. Caminal, J. Lopez-Santin, P. Clapes, *Biocatal. Biotransformation* **2000**, *18*, 253–258.
[88] A. Y. Khimiuk, A. V. Korennykh, L. M. Van Langen, F. Van Rantwijk, R. A. Sheldon, V. K. Švedas, *Tetrahedron Asymmetry* **2003**, *14*, 3123–3128.
[89] M. Mifsud, S. Gargiulo, S. Iborra, I. W. C. E. Arends, F. Hollmann, A. Corma, *Nat. Commun.* **2014**, *5*, 3145.
[90] S. J. P. Willot, E. Fernández-Fueyo, F. Tieves, M. Pesic, M. Alcalde, I. W. C. E. Arends, C. B. Park, F. Hollmann, *ACS Catal.* **2019**, *9*, 890–894.
